# Photochemical Synthesis of Transition Metal-Stabilized Uranium(VI) Nitride Complexes

**DOI:** 10.1038/s41467-022-31582-z

**Published:** 2022-07-01

**Authors:** Xiaoqing Xin, Iskander Douair, Thayalan Rajeshkumar, Yue Zhao, Shuao Wang, Laurent Maron, Congqing Zhu

**Affiliations:** 1 State Key Laboratory of Coordination Chemistry, Jiangsu Key Laboratory of Advanced Organic Materials, School of Chemistry and Chemical Engineering, Nanjing University, Nanjing, 210023 China; 2 LPCNO, CNRS & INSA, Université Paul Sabatier, 135 Avenue de Rangueil, 31077 Toulouse, France; 3 State Key Laboratory of Radiation Medicine and Protection, School for Radiological and interdisciplinary Sciences (RAD-X) and Collaborative Innovation Center of Radiation Medicine of Jiangsu Higher Education Institutions, Soochow University, Suzhou, 215123 China

**Keywords:** Organometallic chemistry, Organometallic chemistry

## Abstract

Uranium nitrides play important roles in dinitrogen activation and functionalization and in chemistry for nuclear fuels, but the synthesis and isolation of the highly reactive uranium(VI) nitrides remains challenging. Here, we report an example of transition metal (TM) stabilized U(VI) nitride complexes, which are generated by the photolysis of azide-bridged U(IV)-TM (TM = Rh, Ir) precursors. The U(V) nitride intermediates with bridged azide ligands are isolated successfully by careful control of the irradiation time, suggesting that the photolysis of azide-bridged U(IV)-TM precursors is a stepwise process. The presence of two U(VI) nitrides stabilized by three TMs is clearly demonstrated by an X-ray crystallographic study. These TM stabilized U(V) nitride intermediates and U(VI) nitride products exhibit excellent stability both in the solid-state and in THF solution under ambient light. Density functional theory calculations show that the photolysis necessary to break the N-N bond of the azide ligands implies excitation from uranium f-orbital to the lowest unoccupied molecular orbital (LUMO), as suggested by the strong antibonding N-(N_2_) character present in the latter.

## Abstract (web summary)

The synthesis and isolation of uranium(VI) nitride complexes remains challenging. Here, the authors report an example of transition metal (TM) stabilized U(VI) nitride complexes, generated via photolysis of azide-bridged U(IV)-TM precursors.

## Introduction

Metal nitride complexes are proposed as key intermediates in N_2_ activation and functionalization^1,2^. Uranium nitride complexes have attracted significant attention due to their potential applications in N_2_ fixation^3–5^, small molecule activation^6–12^, and next-generation nuclear fuels^13–16^. Since uranium nitride materials have been considered to be effective catalysts for the Haber–Bosch synthesis of NH_3_ from N_2_^17^, a series of molecular uranium nitride complexes have been synthesized by N_2_ cleavage or azide reduction, which generally leads to uranium nitride complexes containing U(IV) centers and a few examples with U(III) and U(V) centers^18–32^. However, the synthesis of U(VI) nitrides lags far behind, largely due to the limited synthetic strategy and high chemical reactivity of U(VI) nitrides^33–39^.

**Fig. 1:**
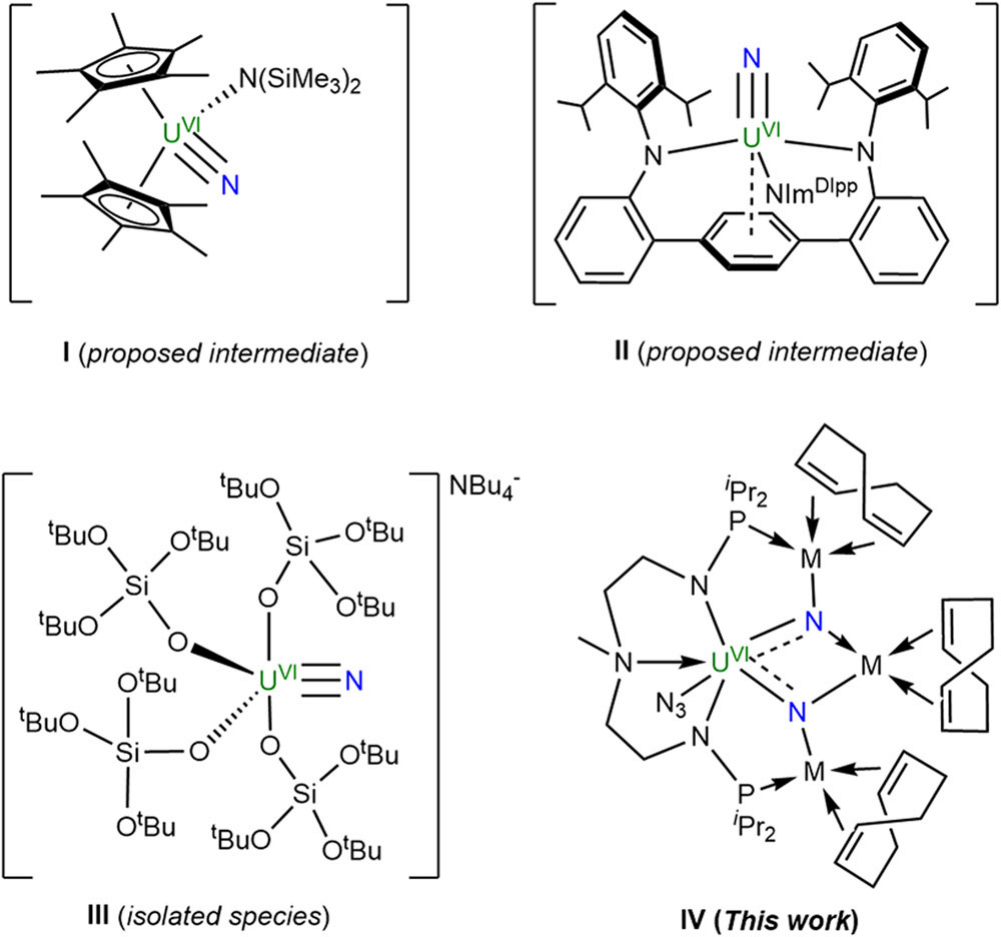
U(VI) nitrides generated by the photolysis of uranium azides. Both **I** and **II** are proposed U(VI) nitride intermediates. Complex **III** is an isolated example of U(VI) nitride species. Complex **IV** is a U(VI) nitride stabilized by transition metals.

Photolysis of metal azides is a typical method in transition metal chemistry to prepare metal nitrides^40–46^. However, despite continuous efforts, this method has been less successful in the synthesis of actinide nitride complexes. For example, Liddle and co-workers found that the U(VI) nitride species is unstable and easily decomposes to U(IV) amide through C–H activation under photolytic conditions, which is consistent with the direct photolysis of U(IV) azide species^33^. Kiplinger, Batista, and co-workers attempted to isolate a U(VI) nitride complex (**I** in Fig. 1) by the photolysis of (C_5_Me_5_)_2_U[N(SiMe_3_)_2_](N_3_), but could only isolate the intramolecular C–H activated product (C_5_Me_5_)(C_5_Me_4_CH_2_NH)U[N(SiMe_3_)_2_^34^. Fortier and co-workers proposed that a U(VI) nitride (**II** in Fig. 1) was formed as a transient intermediate by the photolysis of the corresponding U(IV) azide complex, but the U(VI) nitride also could not be isolated^38^. Recently, Mazzanti and co-workers reported a photochemically robust U(VI) nitride [NBu_4_][U(OSi(O^t^Bu)_3_)_4_(N)] (**III** in Fig. 1) by the photolysis of an anionic U(IV) azide analog^39^. This remains the limited example of the successful isolation of a U(VI) nitride complex via a photolysis route. These results demonstrate that U(VI) nitrides are accessible under photochemical conditions but require an appropriate ligand framework and specific conditions to stabilize this transient U(VI) nitride intermediate.

The introduction of the main group element or another uranium center is an effective method to stabilize the U(VI) nitrides^47–49^. For instance, the Cummins group reported a borane-capped U(VI) nitride complex by chemical oxidation of the U(V) nitridoborate complex^47^. The groups of Hayton and Mazzanti demonstrated that bridged uranium(VI) nitrides with another uranium center are also feasible^48,49^. Recently, our group successfully isolated a U(IV) nitride supported by a multidentate N-P ligand (N(CH_3_)(CH_2_CH_2_NHP^*i*^Pr_2_)_2_) via U-Rh synergistic N_2_ cleavage^28^. Thus we attempted to access U(VI) nitrides with the aid of transition metals by this platform. Herein, we report the synthesis of transition metal-stabilized U(VI) nitrides (**IV** in Fig. 1) by photolysis of U(IV)-azide precursors. The U(V) nitride intermediates are also successfully isolated by careful control of the irradiation time.

## Results

### Synthesis and structural characterization

**Fig. 2:**
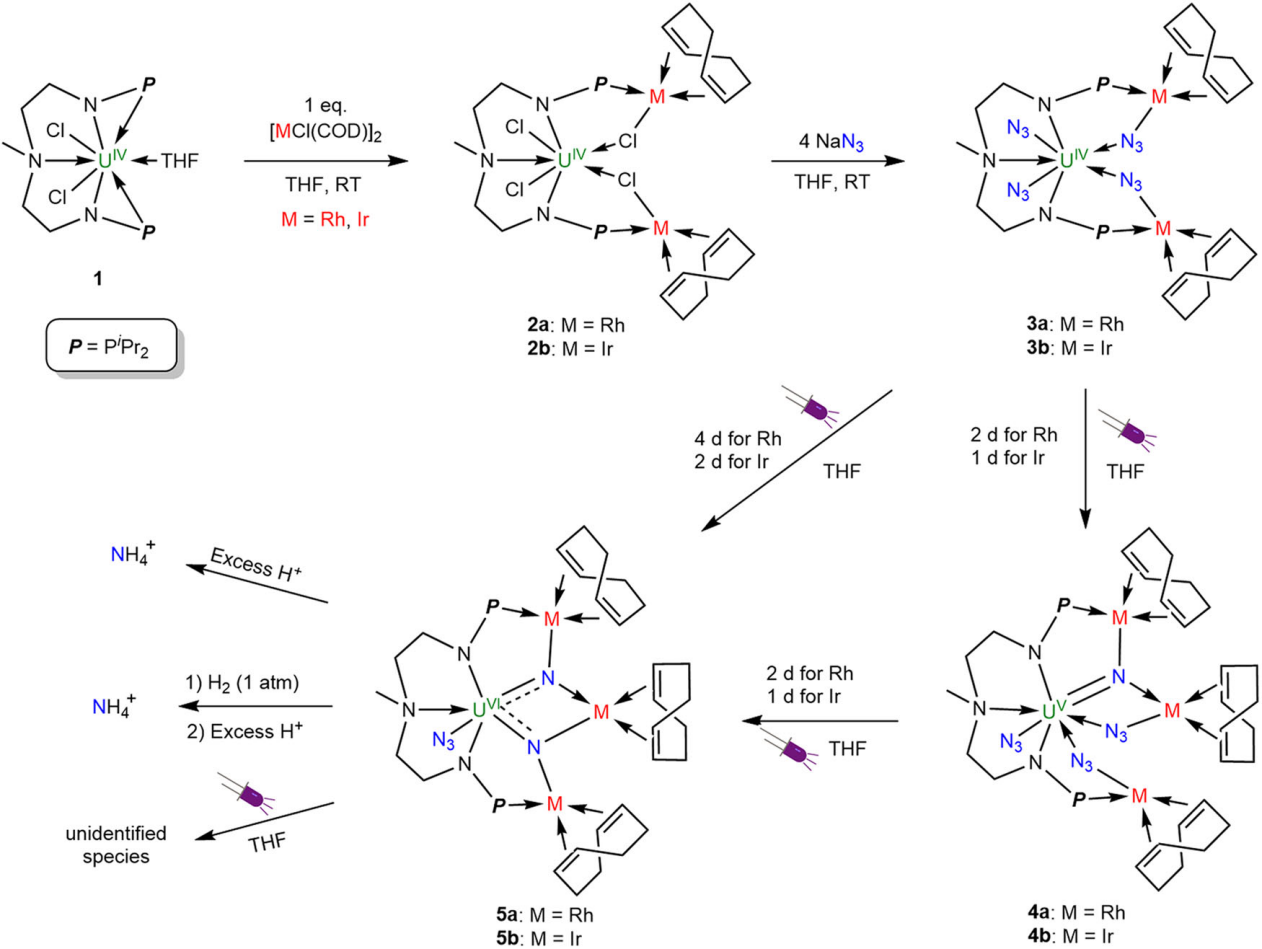
Synthesis and reactivity of complexes 2, 3, 4, and 5. Complexes **3a** and **3b** with four azides were prepared by the reaction of complexes **2** with NaN_3_. By careful control of the irradiation time of complexes **3**, the U(VI) nitrides **5** were formed via the U(V) nitrides intermediates **4**. The nitride groups in complexes **5** react with acid or H_2_/acid to form NH_4_^+^ in good yield. The N atoms originating from N_3_^−^ are shown in blue and transition metals (M) are shown in red.

Upon treatment of complex [{U[N(CH_3_)(CH_2_CH_2_NP^*i*^Pr_2_)_2_](Cl)_2_(THF)}] (**1**)^28^ (Fig. 2) with 1 equiv. of [RhCl(COD)]_2_ (COD = cyclooctadiene) in tetrahydrofuran (THF) at room temperature (RT) overnight, the complex [{U{N(CH_3_)(CH_2_CH_2_NP^*i*^Pr_2_)_2_}(Cl)_2_ [(*μ*-Cl)Rh(COD)]_2_}] (**2a**) was formed and was isolated as green crystals in 81% yield after recrystallization from toluene at −30 °C (Fig. 2). Its iridium analog, [{U{N(CH_3_)(CH_2_CH_2_NP^*i*^Pr_2_)_2_}(Cl)_2_[(*μ*-Cl)Ir(COD)]_2_}] (**2b**), could also be prepared by the reaction of **1** with 1 equiv. of [IrCl(COD)]_2_ following the same procedure, and was isolated as orange crystals in 74% yield. The formation of complexes **2a** and **2b** is analogous to the frustrated Lewis pair reactivity observed previously in rare-earth organometallic chemistry^50–52^. Both **2a** and **2b** were characterized by nuclear magnetic resonance (NMR) spectroscopy (Supplementary Figs. 1 and 2), elemental analysis, and single-crystal X-ray diffraction.

Treatment of **2a** with 4 equiv. of NaN_3_ in THF at RT overnight led to the formation of a uranium(IV) azide complex [{U{N(CH_3_)(CH_2_CH_2_NP^*i*^Pr_2_)_2_}(N_3_)_2_[(*μ-*N_3_)Rh(COD)]_2_}] (**3a**) as brown crystals in 70% yield by generating crystals from the concentrated reaction mixture (Fig. 2). The complex **2b** also reacts with 4 equiv. of NaN_3_ affording the corresponding complex [{U{N(CH_3_)(CH_2_CH_2_NP^*i*^Pr_2_)_2_}(N_3_)_2_[(*μ-*N_3_)Ir(COD)]_2_}] (**3b**) as red crystals in 65% yield. The ^1^H NMR spectra of complexes **3a** and **3b** exhibit a broad range of peaks from +67.46 to −50.01 ppm and +70.12 to −52.63 ppm, respectively, consistent with the paramagnetic U(IV) species (Supplementary Figs. 3 and 4).

**Fig. 3:**
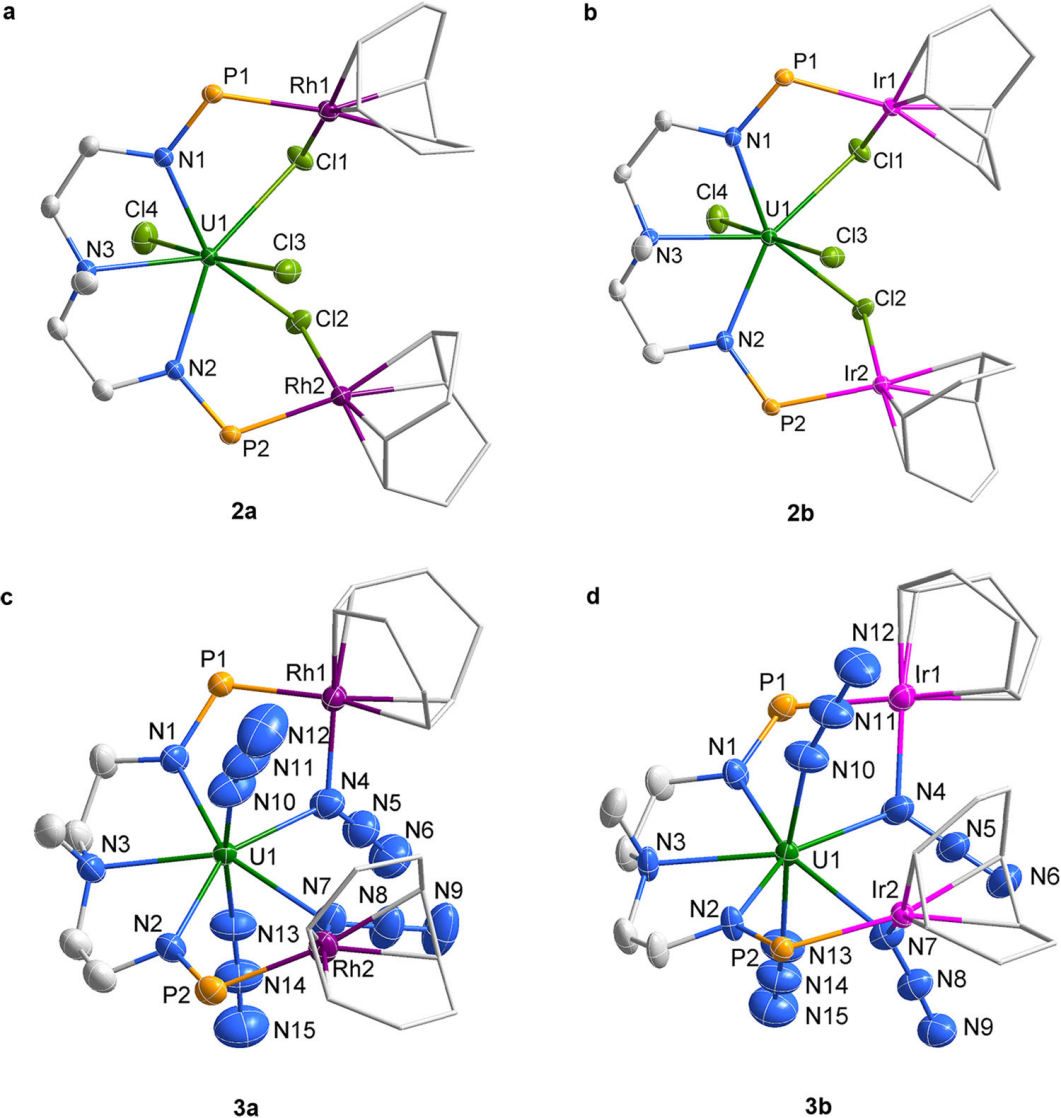
Molecular structures of 2 and 3. **a**–**d** Solid-state structures of **2a** (**a**), **2b** (**b**), **3a** (**c**) and **3b** (**d**) by X-ray crystallography with 50% probability ellipsoids. Solvent molecules, hydrogen atoms, and the isopropyl moieties in P^*i*^Pr_2_ are omitted for clarity. Uranium, green; rhodium, violet; Iridium, pink; phosphorus, orange; nitrogen, blue; chlorine, yellow-green; and carbon, grey.

The asymmetric structures of **2a** and **2b** feature one U atom and two Rh or Ir atoms, which are bridged by two Cl ligands (Fig. 3). The U–N_amido_ and U–N_amine_ bond lengths are comparable to those observed in the precursor **1**. The bridged U–Cl distances are approximately 0.2 Å longer than the terminal U–Cl bonds, revealing a weak interaction between the U center and the bridged Cl atoms.

The molecular structures of complexes **3a** and **3b** were also confirmed by X-ray crystallography (Fig. 3). The salient features of complexes **3a** and **3b** are the two terminal uranium azide ligands and two sideways-bound azide units bridging Rh or Ir atoms. The distances of two-terminal U–N_azide_ bonds in **3a** (2.307(5) and 2.304(5) Å) and **3b** (2.295(8) and 2.311(8) Å) are significantly shorter than the bridged U–N_azide_ bond lengths (2.515(5) and 2.453(5) Å for **3a**, 2.533(8) and 2.475(7) Å for **3b**), but these all fall in the range of previously reported U(IV) azide complexes (2.142(5)−2.564(12) Å)^23,24,34,36,38,53,54^. The bond lengths of Rh–N_azide_ in **3a** (2.091(5) and 2.103(5) Å) and Ir–N_azide_ in **3b** (2.086(8) and 2.072(7) Å) are consistent with the values (2.076(10)−2.28(9) Å) previously reported in complexes with side-on bridged azide ligands^55,56^. The angles of bridged U-N-N (128.7(4)° and 125.2(4)° for **3a**; 128.3(6)° and 124.6(6)° for **3b**) are more distant from the terminal U-N-N (168.6(6)° and 160.5(5)° for **3a**; 165.6(9)° and 161.4(8)° for **3b**).

The reduction, photolysis, and thermolysis of uranium azide complexes are known to be effective methods for the synthesis of uranium nitrides with the release of N_2_^8,21,31,34,36,38,39^. Accordingly, we attempted to synthesize uranium nitride complexes by these routes. Only unidentified products were formed by the reduction of **3** with KC_8_ or thermolysis of **3** at 70 °C for 2 days as suggested by the in-situ ^1^H NMR spectra (Supplementary Figs. 5–8). Photolysis of complex **3a** in THF under UV light irradiation for 2 days resulted in the formation of [{U{N(CH_3_)(CH_2_CH_2_NP^*i*^Pr_2_)_2_}(N_3_)[(μ-N_3_)Rh(COD)]_2_[(*μ*-N)Rh(COD)]}] (**4a**), which was isolated as brown crystals in 54% yield from toluene at RT (Fig. 2). In comparison with **3a**, the ^1^H NMR spectrum of **4a** exhibits a narrow spectral range of +23.37 ppm to −12.46 pm, which is consistent with a previously reported U(V) complex [{U(Tren^DMBS^)}_2_(*μ*-N)] (from +24.86 to −13.31 ppm) and reflects the 5f^1^ nature of these species (Supplementary Fig. 9)^21^. Exposure of complex **3b** to UV light for 1 day resulted in the disappearance of **3b**, and the formation of complex [{U{N(CH_3_)(CH_2_CH_2_NP^*i*^Pr_2_)_2_}(N_3_)[(*μ*-N_3_)Rh(COD)]_2_[(*μ*-N)Rh(COD)]}] (**4b**) as brown crystals in 52% yield after recrystallization (Fig. 2). An in-situ ^1^H NMR study (Supplementary Fig. 10) showed that no paramagnetic uranium by-products were observed in this photolysis although this process involves the dissociation and rearrangement of complexes **3**. Attempts to isolate the uranium by-products were unsuccessful.

After further photolysis of complex **4a** under UV light for 2 days, a diamagnetic U(VI) species [{U{N(CH_3_)(CH_2_CH_2_NP^*i*^Pr_2_)_2_}(N_3_){[(*μ*-N)Rh(COD)]_2_[Rh(COD)]}}] (**5a**) was isolated in 65% yield after recrystallization (Fig. 2). Similar to the Rh analogs, complex **4b** could be further photolysed under UV light for 1 day, leading to the formation of the complex [{U{N(CH_3_)(CH_2_CH_2_NP^*i*^Pr_2_)_2_}(N_3_){[(*μ*-N)Ir(COD)]_2_[Ir(COD)]}}] (**5b**) as brown crystals in 58% yield. The U(V) centers in complexes **4a** and **4b** were oxidized to U(VI) in complexes **5a** and **5b** in this photolysis process. Complexes **4** and **5** are heterobimetallic bridged nitrides featuring uranium and transition metals. Both **5a** and **5b** could be obtained by the photolysis of **3a** and **3b** under UV light for 4 and 2 days, respectively. These results suggest that the photolysis of **3** is controllable and that complexes **4a** and **4b** can be viewed as intermediates in these processes.

**Fig. 4:**
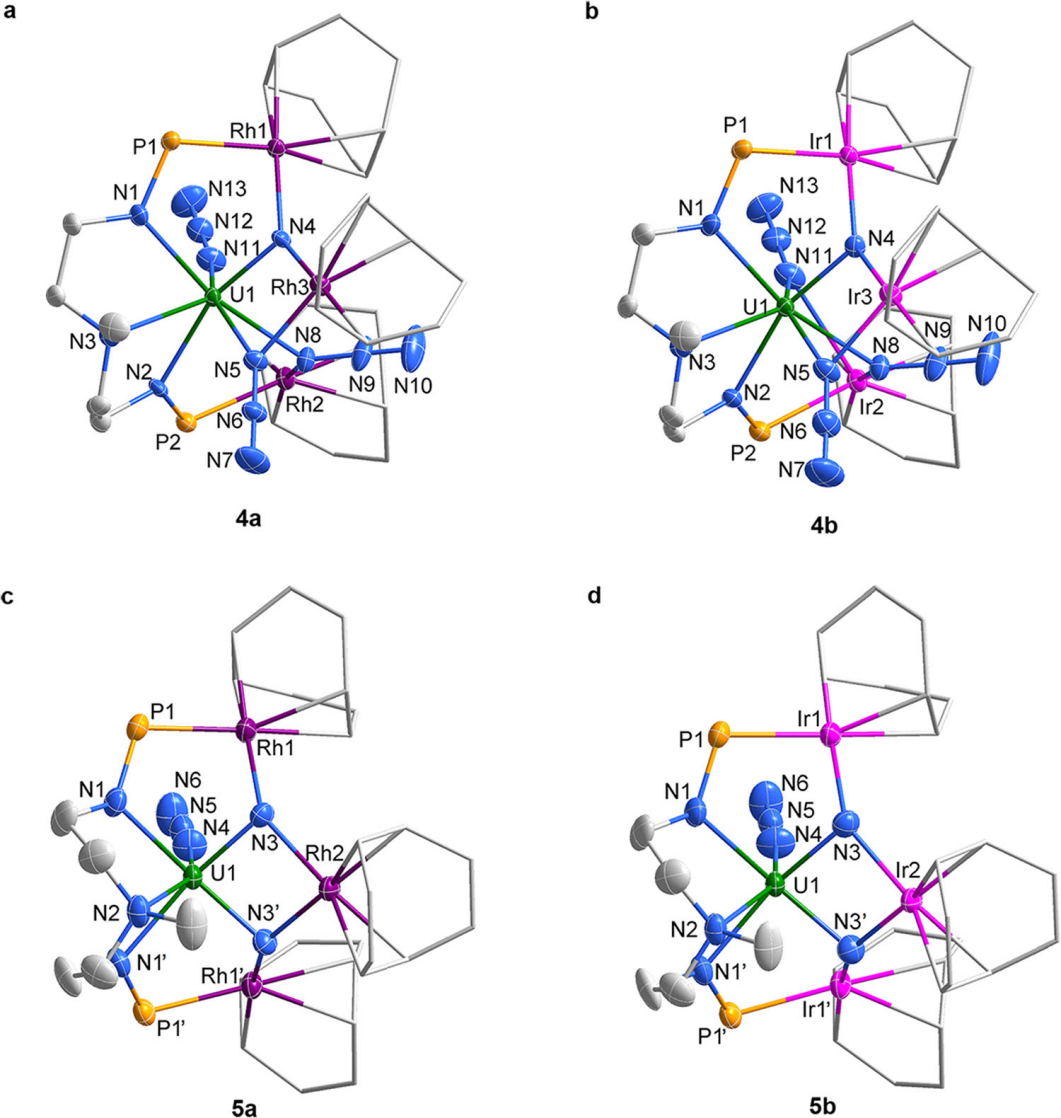
Molecular structures of 4 and 5. **a**–**d** Solid-state structures of **4a** (**a**), **4b** (**b**), **5a** (**c**), and **5b** (**d**) by X-ray crystallography with 50% probability ellipsoids. Solvent molecules, hydrogen atoms, and the isopropyl moieties in P^*i*^Pr_2_ are omitted for clarity. Uranium, green; rhodium, violet; Iridium, pink; phosphorus, orange; nitrogen, blue; and carbon, grey.

The solid-state structures of **4** and **5** were determined by single-crystal X-ray diffraction. The U centers in **4a** and **4b** are coordinated with a tridentate [N(CH_3_)(CH_2_CH_2_NP^*i*^Pr_2_)_2_]^2−^ ligand, three azide ligands, and a nitride ligand and have a distorted pentagonal bipyramidal geometry (Fig. 4). The nitride atom (N4) is bonded to one U atom and two Rh atoms. The U1-N4 distances are 1.963(5) Å in **4a** and 2.011(4) Å in **4b**, which are shorter than the U-N multiple bond lengths in a uranium-stabilized U(V) nitride complex with a U(V)=N=U(V) unit (2.0470(3) and 2.0511(3) Å)^22^. For further comparison, the U-N_nitride_ bond distances in the sodium- or borane-capped U(V) nitrides are 1.883(4) Å and 1.916(4) Å, respectively^30,47^. The Rh/Ir–N4 bond lengths in **4a** and **4b** are close to the Rh/Ir–N_azide_ bond lengths and are comparable to those observed in **3a** and **3b**, respectively.

The centrosymmetric structures of **5** feature two bridged nitride atoms, which are capped by three Rh or Ir atoms (Fig. 4). The bond lengths of U1–N3 in **5a** (1.975(5) Å) and **5b** (2.005(6) Å) are comparable with the U(V)-N_nitride_ bond lengths found in complexes **4a** and **4b**, respectively. However, the U(VI)-N_nitride_ bond lengths in complexes **5** are longer than those in the U(VI)-N_imido_ bond in a *trans*-imido uranium complex (1.840(4)−1.866(2) Å)^57^ or in the U(VI)-N_nitride_ bond in a uranyl analog [OUN]^+^ (1.818(9) Å)^48^. Two resonance structures (Supplementary Fig. 33) are mainly responsible for the longer U(VI)-N_nitride_ bond lengths in complexes **5**. The bond lengths of Rh-N3 (2.082(4) Å and 2.067(4) Å) and Ir–N3 (2.059(6) Å and 2.047(6) Å) in **5a** and **5b** are comparable to the Rh/Ir-N4 bond distances observed in **4a** and **4b**, respectively. Complexes **5** represent an example of a transition metal-stabilized U(VI) nitride species, which is generated by the photolysis of a uranium azide species.

### Stability and reactivity of uranium-nitride complexes

The combination of late transition metal (Rh and Ir) in U(V) nitrides **4** and U(VI) nitrides **5** are different from the main group borane-capped U(VI) nitride^47^ or another uranium-stabilized U(VI) nitrides^48,49^. Consequently, the stabilities of the newly synthesized uranium nitrides **4** and **5** were investigated. Both **4** and **5** exhibits good stability in the solid-state or in THF solution for at least 2 weeks under a N_2_ atmosphere, probably because the active nitrides are stabilized by transition metals. Irradiation of U(V)-nitride-azide complexes **4a** and **4b** with UV light for 2 and 1 days resulted in the formation of U(VI)-nitride complexes **5a** and **5b**, respectively (Fig. 2). However, longer irradiation times than those used in the formation of **5** decompose the product to compounds which could not be isolated or identified.

The protonation of metal nitrides to produce NH_3_ is a critical step in N_2_ reduction and conversion. Thus, we examined the reactivity of **4** and **5** toward acid and H_2_ (Fig. 2). We found that substantial NH_4_Cl (in 68-82% yields) was formed by the reactions of uranium nitride complexes **4** and **5** with excess PyHCl in THF solution (Supplementary Figs. 14–17). The addition of 1 atm H_2_ to the crystalline solids or to the degassed THF solution of **4** and **5**, led to a gentle color change from brown to red. Unfortunately, attempts to isolate the single crystals of the resulting products were unsuccessful. However, the addition of excess PyHCl to the reaction mixtures of **4** and **5** with H_2_ in THF solution led to the formation of NH_4_Cl in 41–67% yields, respectively (Supplementary Figs. 18–21). In comparison, no NH_4_^+^ was observed from the reactions of uranium azide complexes **3** with excess PyHCl (Supplementary Fig. 22). These results suggest that the nitride units in complexes **4** and **5** are the sources of the NH_4_^+^.

### Magnetic studies and electronic absorption spectra

**Fig. 5:**
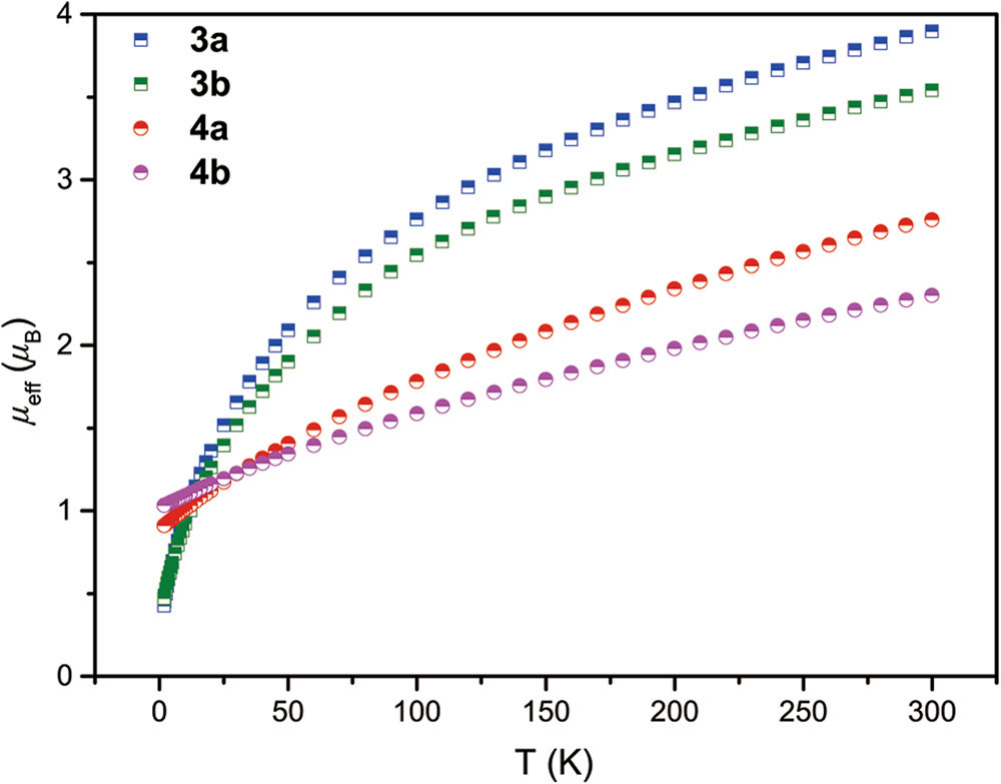
Magnetic data. Variable-temperature effective magnetic moment data of **3a** (blue), **3b** (green), **4a** (red) and **4b** (pink).

The variable-temperature magnetic data of complexes **3** and **4** in the solid-state were measured by a superconducting quantum interference device (SQUID) magnetometry (Fig. 5). The magnetic moments of **3a** and **3b** at 300 K are 3.90 μ_B_ and 3.54 μ_B_, respectively, which are close to the theoretical value (3.58 *μ*_B_) for the 5*f*^2^ uranium ion in the ^3^H_4_ ground state. With decreasing temperatures, the magnetic moments of **3a** and **3b** decrease persistently to 0.43 μ_B_ and 0.47 μ_B_ at 1.8 K, respectively, and with a trend to zero, which is characteristic of U(IV) complexes^58,59^. The effective magnetic moments of **4a** and **4b** at 300 K are 2.76 μ_B_ and 2.30 μ_B_, respectively, which are very close to the theoretical value of 2.54 μ_B_ for one U(V) ion. The magnetic moments of **4a** and **4b** decrease smoothly to 0.91 μ_B_ and 1.03 μ_B_ at 1.8 K, respectively, with decreasing temperature. The temperature dependence and the magnitude of *μ*_eff_ values of complexes **4a** and **4b** are consistent with reported U(V) complexes^30,59^. These results suggest that the oxidation states of uranium centers in complexes **3** and **4** are U(IV) and U(V), respectively.

The ultraviolet/visible/near-infrared (UV/Vis/NIR) electronic absorption spectra of complexes **3**-**5** were recorded in THF at RT (Supplementary Figs. 27–32). These complexes exhibit similar absorption profiles with gradually decreasing molar absorption at high energy in the UV–Vis region but show different fingerprint features in the NIR region. Complex **3a** has a moderately intense absorption peak at 413 nm (ε = 4265 M^−1^ cm^−1^), while complex **3b** exhibits three moderate intense peaks at 330, 382, and 449 nm (ε = 3060–6518 M^−1^ cm^−1^). These absorptions can be assigned to ligand-to-metal charge-transfer transition. Complex **4a** exhibits a moderately intense charge-transfer band at 413 nm (ε = 3731 M^−1^ cm^−1^) while **4b** displays three peaks at 333, 378, and 446 nm (ε = 3159–7154 M^−1^ cm^−1^). Consistently, the charge-transfer transitions in **5a** and **5b** are observed at 451 nm (ε = 3384 M^−1^ cm^−1^) and 333, 382, and 451 nm (ε = 3081–5870 M^−1^ cm^−1^), which are similar to the spectra of **3a** and **3b**, respectively. In the NIR region, broad, but very weak absorption bands from 975 to 1200 nm (ε < 70 M^−1^ cm^−1^) were observed for **3a** and **3b**, which were assigned to f–f transitions and were consistent with the U(IV) complexes with 5f^2^ electronic configuration^60–62^. Complexes **4a** and **4b** exhibit similar weak absorption bands at 1030, 1310, and 1630 nm (ε < 90 M^−1^ cm^−1^) in the NIR region, which could be attributed to *f-f* transitions of U(V) complexes with 5f^1^ electronic configuration^63,64^. As expected for a U(VI) species with a 5f^0^ electronic configuration, the spectra of **5a** and **5b** show no characteristic absorption peak in the NIR region (900–1700 nm), consistent with previously reported U(VI) complexes [U(N)(Tren^TIPS^)]^33^ and [NBu_4_][U(OSi(O^t^Bu)_3_)_4_(N)]^39^.

### Theoretical studies

DFT calculations (B3PW91) were used to describe the bonding properties of these complexes, in particular complexes **4** and **5**. To probe the validity of the computational approach, the geometry of each of the different complexes was compared with the experimental geometry. The two-terminal U–N_azide_ bonds are well reproduced (2.27/2.30 Å in **3a** and 2.27/2.30 Å in **3b**). The same is true for the two bridged U–N_azide_ bonds (2.49/2.52 Å in **3a** and 2.51/2.53 Å in **3b**). In the same way, the Rh-N_azide_ bonds are correctly described (2.11 Å) indicating that the computational method is appropriate to describe such complexes. The same results were obtained for complexes **4a**/**4b** and **5a**/**5b** with the same accuracy and a maximum deviation of 0.05 Å (see Supplementary Tables 11, 14, 17, and 20). It is interesting to note that the LUMOs of complexes **3a** and **3b** (Supplementary Figs. 43 and 50) imply mainly two natural localized molecular orbitals (NLMOs), one being an antibonding π interaction between N8 and N9 and one with a bonding interaction between N7 and U1 as found in the canonical molecular orbital (CMO) analysis. Moreover, the bonding of the azide ligand with both U and Rh leads to a non-symmetrical contribution of the two N-N bonds to the N-N-N hyperbond. Indeed, for a free azide, each N-N bond contributes to 50% to the hyperbond N-N-N while in complex **3a** for instance the N7-N8 only contributes to 37% to the hyperbond. This clearly means that the coordination of the azide ligand to both U and Rh leads to a relocalization of the density between N8 and N9 which would ultimately lead to the N_2_ formation and loss from the azide. It is interesting to notice that according to these orbital energies, the calculated SOMO-LUMO gap is 78.0 kcal mol^−1^ explaining that photolysis is required to make complexes **3a**/**3b** evolve and to form complexes **4a**/**4b**. Moreover, it is interesting to note that the formation of the two latter complexes from **3a**/**3b** is thermodynamically favorable (by 17.6 kcal mol^−1^ for **4a** and 18.2 kcal mol^−1^ for **4b**). The bonding situation was thus analyzed for complexes **4a** and **4b** and the bond lengths were found to be well reproduced.

**Fig. 6:**
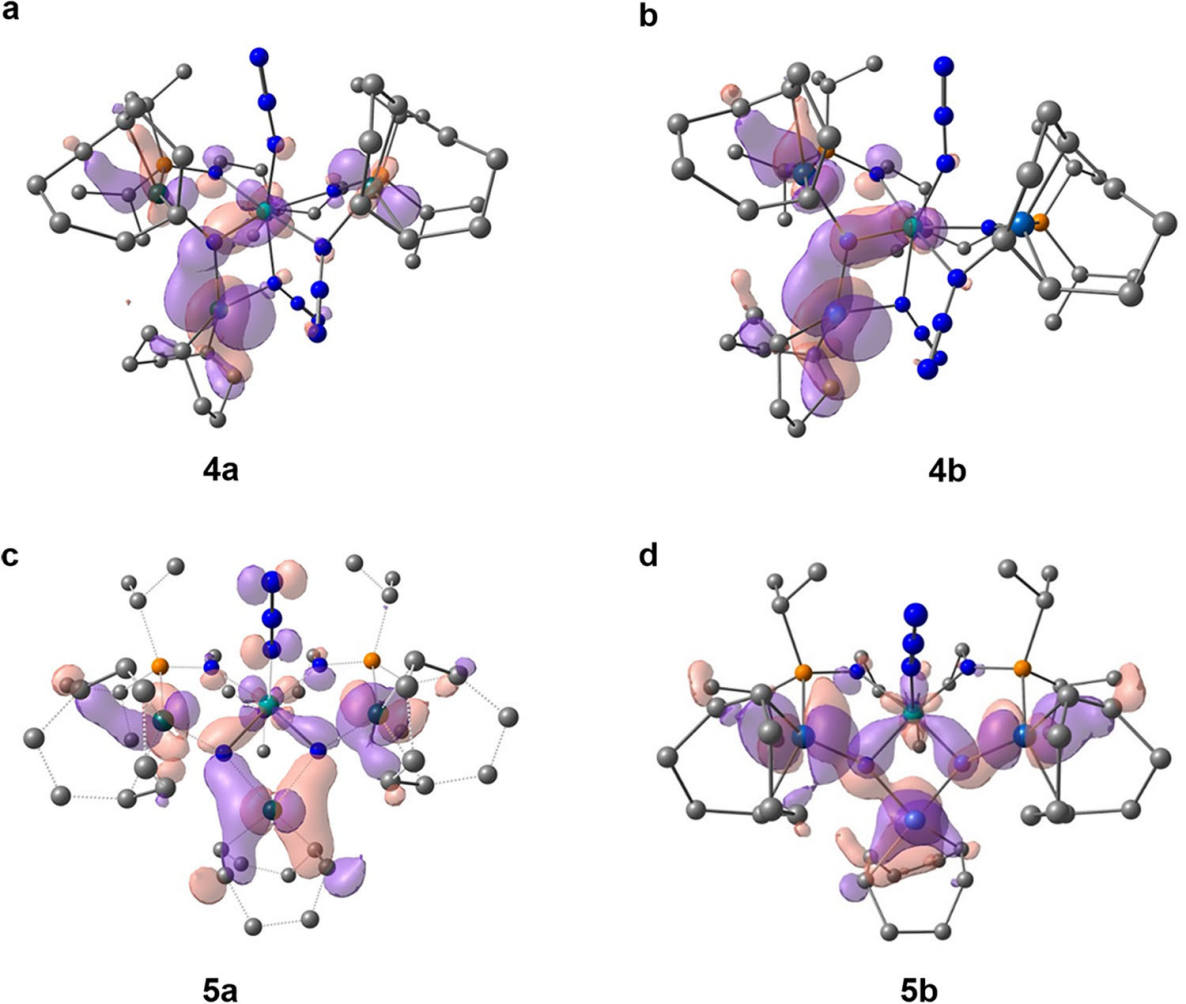
Molecular orbitals. U-N-Rh(Ir) bonding molecular orbitals of complexes **4a** (**a**), **4b** (**b**), **5a** (**c**) and **5b** (**d**).

Analysis of the unpaired spin densities (1.23 in **4a** and 1.24 in **4b**), clearly indicates the presence of a U(V) metal center in these two complexes. At the NBO level, the U1-N4 bond is found to be a double bond with polarized bonds toward N (68% for σ and 72% for π in **4a** and 72/75% in **4b**, Supplementary Table 10). These bonds involve *fd* hybrid orbitals on uranium (78% 5f-20% 6d for σ and 59% 5f-39% 6d for π in **4a** but 59% 5f-33% 6d and 57% 5f-33% 6d in **4b**). Although polarized, these bonds appear to be relatively covalent as demonstrated by the U1-N4 Wiberg Bond Index (WBI) of 1.96 in **4a** (1.83 in **4b**). The associated Rh1-N4 bond was only observed at the second-order donor-acceptor level but U-N-Rh interactions are found in the NLMO as a 3-centers bond, the associated WBI being 0.45. On the other hand, the Ir1-N4 bonds in **4b** are similar to the Rh-N ones in **4a** with a WBI of 0.49. The NBO decomposition of the NLMOs associated with the U-N4 bonding interactions (Supplementary Table 13) shows some delocalization toward the Rh centers (resp. Ir, Supplementary Table 19), in line with some 3c-2e bonds. This is similar to what was found by scrutinizing the molecular orbitals (MO, Fig. 6), the U1-N4-Rh(Ir) bonds in **4a** and **4b** are 3c-2e bonds. Similar to complexes **3a** and **3b**, the LUMOs of complexes **4a** and **4b** (Supplementary Figs. 45 and 52) also display some antibonding interactions in the azide ligands. According to the orbital energy, the SOMO-LUMO energy gap is 80.0 kcal mol^−1^ consistent with the need of photolysis to make complexes **4a/4b** to evolve. For the transformation of **4a**/**4b** into complexes **5a**/**5b**, the reaction is thermodynamically favored by 43.5 kcal mol^−1^ (**5a**) and 49.6 kcal mol^−1^ (**5b**).

Finally, the bonding in complexes **5a**/**5b** was analyzed. The optimized geometries compare well with the experimentally observed geometries. In both complexes, the uranium centers are at the oxidation state +VI and have no residual spin densities. The two double U-N bonds are similar to those found in complexes **4** with a WBI of 1.91 for **5a** (1.80 for **5b**), and the NLMO, as well as the MO, clearly indicate 3c-2e U-N-Rh(Ir) bonds (Fig. 6). The slight decrease observed of the WBI and therefore of the covalency while going from complexes **4** to **5** might be explained by the strength of the U-Rh interaction in the two systems. Indeed, in **4** where the U-M distance is around the sum of the van der walls radii, the U…Rh WBI is 0.10 whereas in **5** where the distance is shorter, and the WBI is larger (0.22). The stronger U-Rh interaction would thus decrease the U-N_nitrido_ interaction. At the NBO level, the U1-N3 and U1-N3’ bonds are found to be U-N double bonds polarized toward N and these bonds also involve *fd* hybrid orbitals on uranium (Supplementary Table 10).

## Discussion

In summary, transition metal stabilized U(VI) nitride complexes **5a** and **5b** were generated by photolysis of U(IV) azide precursors, **3a** and **3b**, respectively. The U(V) nitride intermediates **4a** and **4b** were successfully isolated by controlling the duration of the irradiation. They were sufficiently stable to be fully characterized and could be further photolyzed to the U(VI) nitrides **5** under UV irradiation conditions. Therefore, the photolysis of the azide units in complexes **3a** and **3b** is controllable and is a stepwise process. The excellent stabilities of both the U(V) nitride intermediates **4** and the U(VI) nitride products **5** are probably due to the active nitrides being stabilized by transition metals. A DFT study allowed us to analyze the bonding of these complexes, indicating the presence of U-N multiple bonds and U-N-Rh(Ir) 3c-2e interactions. Moreover, DFT calculations showed that the photolysis, which is necessary to break the N-N bond of the azide ligands, suggests excitation from an f-electron of the metal to the LUMOs of complexes **3** and **4**, and this primarily displays an antibonding N-(N_2_) interaction. This study implies that a multiple N-P ligand is an effective platform with which to capture unstable U(VI) nitrides with the aid of TMs^65^.

## Methods

### General considerations

Experiments were performed under argon or nitrogen atmosphere using standard Schlenk-line and glove-box techniques. Solvents were dried and degassed with a solvent purification system before use. See the supplementary information for detailed experimental procedures, crystallographic analyses (Supplementary Figs. 34–41 and Supplementary Tables 1–9), and computational details (Supplementary Dataset, Supplementary Figs. 42–56, and Supplementary Tables 10–22).

### Synthesis of 2a and 2b

A solution of complex **1** (72 mg, 0.1 mmol, 1 equiv.) in THF was added dropwise to a THF solution of [RhCl(COD)]_2_ (49 mg, 0.1 mmol, 1 equiv.) under an atmosphere of nitrogen. The mixture was stirred overnight at RT, and then the solvents were removed under reduced pressure and the residues were extracted with toluene. The filtrate was concentrated to 1 mL and placed at −30 °C for 24 h. Green crystals of **2a** (93 mg, 81%) suitable for X-ray diffraction were obtained. Orange crystals of **2b** (98 mg, 74%) were obtained following the same procedure in which complex **1** (72 mg, 0.1 mmol, 1 equiv.) was treated with 1 equiv. [IrCl(COD)]_2_ (67 mg, 0.1 mmol, 1 equiv.). **2a**: ^1^H NMR (THF-d_8_, 400 MHz, ppm) δ 25.93 (s, 2H), 20.98 (s, 6H), 17.66 (s, 2H), 16.78 (s, 2H), 14.16 (s, 2H), 13.35 (s, 2H), 11.06 (s, 2H), 10.35 (s, 12H), 8.71 (s, 6H), 5.15 (s, 6H), 2.80 (s, 12H), −1.19 (s, 2H), −3.12 (s, 2H), −4.04 (s, 2H), −39.61 (s, 1H), −42.76 (s, 1H), −46.13 (s, 1H). ^31^P{^1^H} NMR (THF-d_8_, 162 MHz, ppm) δ 78.94 (d, ^1^*J*_P-Rh_ = 392 Hz), −87.20 (br). Anal. Calcd. for C_33_H_63_Cl_4_Rh_2_N_3_P_2_U^.^toluene: C, 38.69; H, 5.76; N, 3.38. Found: C, 38.12; H, 5.88; N, 3.36. **2b**: ^1^H NMR (THF-d_8_, 400 MHz, ppm) δ 22.79 (s, 2H), 19.36 (s, 6H), 16.40 (s, 3H), 15.35 (s, 3H), 11.84 (s, 6H), 9.55 (s, 2H), 8.85 (s, 7H), 8.29 (s, 3H), 7.50 (s, 6H), 5.87 (s, 2H), 4.82 (s, 1H), 3.65 (s, 1H), 3.24 (s, 1H), −0.47 (s, 4H), −1.73 (s, 4H), −3.29 (s, 4H), −10.87 (s, 1H), −31.83 (s, 1H), −39.26 (s, 2H), −40.94 (s, 2H), −49.69 (s, 2H). ^31^P{^1^H} NMR (THF-d_8_, 162 MHz, ppm) δ 63.59, −150.90. Anal. Calcd. for C_33_H_63_Cl_4_Ir_2_N_3_P_2_U^.^toluene: C, 33.83; H, 5.04; N, 2.96. Found: C, 33.67; H, 5.20; N, 2.92.

### Synthesis of 3a and 3b

A solution of complex **2a** (57 mg, 0.05 mmol, 1 equiv.) in THF was added to a mixture of NaN_3_ (13 mg, 0.2 mmol, 4 equiv.) in THF under an atmosphere of nitrogen. The mixture was stirred overnight at RT and then the mixture was filtered. The filtrate was concentrated to 1 mL and placed at −30 °C for 24 h, producing brown crystals of **3a** suitable for X-ray diffraction (41 mg, 70%). Red crystals of **3b** (44 mg, 65%) were obtained following the same procedure by treatment of **2b** (66 mg, 0.05 mmol, 1 equiv.) with 4 equiv. NaN_3_ (13 mg, 0.2 mmol, 4 equiv.). **3a**: ^1^H NMR (THF-d_8_, 400 MHz, ppm) δ 67.46 (s, 1H), 59.45 (s, 1H), 19.80 (s, 6H), 7.00 (s, 12H), −3.17 (s, 12H), −8.97 (s, 6H), −10.60 (s, 6H), −12.85 (s, 6H), −15.62 (s, 1H), −22.34 (s, 2H), −25.67 (s, 1H), −31.81 (s, 2H), −42.28 (s, 2H), −44.68 (s, 3H), −49.98 (s, 1H), −50.01 (s, 1H). ^31^P{^1^H} NMR (THF-d_8_, 162 MHz, ppm) δ 76.79 (d, ^1^*J*_P-Rh_ = 392 Hz), 77.58 (d, ^1^*J*_P-Rh_ = 396 Hz). Anal. Calcd. for C_33_H_63_N_15_P_2_Rh_2_U^.^THF: C, 35.61; H, 5.74; N, 16.84. Found: C, 35.80; H, 5.73; N, 16.44. **3b**: ^1^H NMR (THF-d_8_, 400 MHz, ppm) δ 70.12 (s, 1H), 61.77 (s, 1H), 21.97 (s, 6H), 5.05 (s, 12H), −2.46 (s, 12H), −9.88 (s, 6H), −11.40 (s, 6H), −13.63 (s, 6H), −15.90 (s, 2H), −23.74 (s, 2H), 33.15 (s, 1H), −33.19 (s, 1H), −42.70 (s, 2H), −46.20 (s, 3H), −52.59 (s, 1H), −52.63 (s, 1H). ^31^P{^1^H} NMR (THF-d_8_, 162 MHz, ppm) δ 64.60, 64.41. Anal. Calcd. for C_33_H_63_Ir_2_N_15_P_2_U^.^THF: C, 31.15; H, 5.02; N, 14.73. Found: C, 31.17; H, 4.98; N, 13.99.

### Synthesis of 4a and 4b

A solution of complex **3a** (59 mg, 0.05 mmol, 1 equiv.) in THF (5 mL) was transferred into a Schlenk tube under an atmosphere of nitrogen and then irradiated with a 40 W UV lamp for 2 days. The solvents were removed under reduced pressure and the residues were extracted with toluene. The filtrate was concentrated to 0.5 mL and placed at RT for 48 h, giving brown crystals of **4a** suitable for X-ray diffraction (24 mg, 54%). Brown crystals of **4b** were obtained following the same procedure by exposing a THF solution of complex **3b** (68 mg, 0.05 mmol, 1 equiv.) to UV light for 1 days (28 mg, 52%). **4a**: ^1^H NMR (THF-d_8_, 400 MHz, ppm) δ 23.37 (s, 1H), 15.28 (s, 2H), 12.58 (s, 1H), 9.79 (s, 1H), 9.49 (s, 1H), 9.08 (s, 3H), 8.33 (s, 3H), 8.18 (s, 1H), 6.89 (s, 1H), 6.69 (s, 1H), 6.23 (s, 2H), 5.80 (s, 2H), 5.62 (s, 2H), 5.53 (s, 1H), 5.26 (s, 2H), 5.04 (s, 2H), 4.88 (s, 3H), 4.82 (s, 1H), 4.47 (s, 1H), 4.40 (s, 6H), 3.77 (s, 6H), 3.68 (s, 1H), 2.95 (s, 2H), 1.28 (s, 6H), 0.99 (s, 2H), 0.66 (s, 2H), 0.46 (s, 3H), 0.02 (s, 2H), −0.39 (s, 2H), −0.77 (s, 2H), −2.89 (s, 1H), −4.05 (s, 1H), −5.45 (s, 1H), −6.08 (s, 1H), −8.05 (s, 4H), −10.79 (s, 1H), −12.46 (s, 1H). ^31^P{^1^H} NMR (THF-d_8_, 162 MHz, ppm) δ 94.65 (br), 76.79 (d, ^1^*J*_P-Rh_ = 392 Hz). Anal. Calcd. for C_41_H_75_N_13_P_2_Rh_3_U: C, 36.24; H, 5.56; N, 13.40. Found: C, 36.59; H, 5.63; N, 13.35. **4b**: ^1^H NMR (THF-d_8_, 400 MHz, ppm) δ 24.73 (s, 1H), 16.26 (s, 1H), 15.08 (s, 1H), 12.34 (s, 1H), 11.32 (s, 1H), 10.70 (s, 1H), 10.42 (s, 1H), 9.53 (s, 3H), 8.26 (s, 1H), 7.58 (s, 1H), 6.66 (s, 2H), 6.47 (s, 3H), 6.17 (s, 2H), 5.96 (s, 2H), 5.33 (s, 4H), 5.15 (s, 2H), 5.09 (s, 3H), 4.85 (s, 2H), 4.73 (s, 6H), 4.55 (s, 6H), 4.46 (s, 1H), 4.35 (s, 2H), 4.19 (s, 2H), 1.56 (s, 3H), 0.96 (s, 6H), 0.90 (s, 2H), −0.40 (s, 2H), −0.40 (s, 2H), −0.48 (s, 2H), −3.20 (s, 1H), −5.36 (s, 1H), −6.87 (s, 1H), −9.53 (s, 4H), −10.95 (s, 1H), −12.17 (s, 2H), −12.48 (s, 1H). ^31^P{^1^H} NMR (THF-d_8_, 162 MHz, ppm) δ 73.08, 68.63. Anal. Calcd. for C_41_H_75_N_13_P_2_Ir_3_U^.^1.5toluene: C, 35.05; H, 4.97; N, 10.32. Found: C, 35.14; H, 4.99; N, 10.44.

### Synthesis of 5a and 5b

**Method A:** A solution of complex **4a** (34 mg, 0.025 mmol, 1 equiv.) in THF (5 mL) was transferred into a Schlenk tube under an atmosphere of nitrogen and then irradiated with a 40 W UV lamp for 2 days. The mixture was filtered and the filtrate was concentrated to 0.5 mL and placed at RT for 48 h, brown crystals of **5a** suitable for X-ray diffraction were obtained (21 mg, 65%). Brown crystals of **4b** were obtained following the same procedure by exposing a THF solution of complex **5b** (41 mg, 0.025 mmol, 1 equiv.) to UV light for 1 days (23 mg, 58%). **Method B:** A solution of complex **3a** (59 mg, 0.05 mmol, 1 equiv.) in THF (5 mL) was transferred into a Schlenk tube under an atmosphere of nitrogen and then irradiated with a 40 W UV lamp for 4 days. The mixture was filtered and the filtrate was concentrated to 0.5 mL and placed at RT for 48 h, brown crystals of **5a** suitable for X-ray diffraction were obtained (16 mg, 38%). Brown crystals of **5b** were obtained using the same procedure by exposing a THF solution of complex **3b** (68 mg, 0.05 mmol, 1 equiv.) to UV light for 2 days (17 mg, 32%). **5a**: ^1^H NMR (THF-d_8_, 400 MHz, ppm) δ 6.36 (s, 2H), 5.88 (s, 2H), 4.91-4.95 (m, 5H), 3.34-3.42 (m, 6H), 2.84–2.96 (m, 6H), 2.35–2.42 (m, 12H), 2.15–2.20 (m, 6H), 1.54–1.57 (m, 12H), 1.10–1.19 (m, 24H). ^31^P{^1^H} NMR (THF-d_8_, 162 MHz, ppm) δ 41.48 (d, ^1^*J*_P-Rh_ = 372 Hz). Anal. Calcd. for C_41_H_75_N_8_P_2_Rh_3_U: C, 38.21; H, 5.87; N, 8.69. Found: C, 38.24; H, 5.97; N, 8.50. **5b**: ^1^H NMR (THF-d_8_, 400 MHz, ppm) δ 6.36 (s, 2H), 5.88 (s, 2H), 4.56–4.57 (m, 5H), 3.36–3.43 (m, 6H), 2.88–2.94 (m, 6H), 2.40–2.48 (m, 12H), 2.18–2.22 (m, 6H), 1.51–1.58 (m, 12H), 1.23–1.27 (m, 24H). ^31^P{^1^H} NMR (THF-d_8_, 162 MHz, ppm) δ 29.30. Anal. Calcd. for C_41_H_75_N_8_P_2_Ir_3_U: C, 31.63; H, 4.86; N, 7.20. Found: C, 32.63; H, 4.89; N, 7.36.

## Supplementary information

Supplementary Information

Description of Additional Supplementary Files

Supplementary Data 1

## Data Availability

The X-ray crystallographic coordinates for structures reported in this study have been deposited at the Cambridge Crystallographic Data Centre (CCDC), under deposition numbers CCDC-2104709 (**2a**), 2104710 (**2b**), 2104711 (**3a**), 2104712 (**3b**), 2104713 (**4a**), 2104714 (**4b**), 2104715 (**5a**), and 2104716 (**5b**). These data can be obtained free of charge from the CCDC via www.ccdc.cam.ac.uk/data_request/cif. The cartesian coordinates see the supplementary data file. The data that support the findings of this study are available from the corresponding author upon reasonable request.
