# Supplementary material for: Photochemical Synthesis of Transition Metal-Stabilized Uranium(VI) Nitride Complexes
Source: Nat Commun. 2022 Jul 1;13:3809. doi: 10.1038/s41467-022-31582-z (PMC9249861; doi:10.1038/s41467-022-31582-z)
Supplement: Supplementary file 1 — Supplementary Information [file 41467_2022_31582_MOESM1_ESM.pdf]

Supplementary Information

for

Photochemical Synthesis of Transition Metal-Stabilized Uranium(VI) Nitride Complexes

Xiaoqing Xin,[1] Iskander Douair,[2] Thayalan Rajeshkumar,[2] Yue Zhao,[1] Shuao Wang,[3] Laurent Maron,[2]* and Congqing Zhu[1]*

[1]State Key Laboratory of Coordination Chemistry, Jiangsu Key Laboratory of Advanced Organic Materials, School of Chemistry and Chemical Engineering, Nanjing University, Nanjing 210023, China

[2]LPCNO, CNRS & INSA, Université Paul Sabatier, 135 Avenue de Rangueil, 31077 Toulouse, France

[3]State Key Laboratory of Radiation Medicine and Protection, School for Radiological and interdisciplinary Sciences (RAD-X) and Collaborative Innovation Center of Radiation Medicine of Jiangsu Higher Education Institutions, Soochow University, Suzhou, China

*Correspondence and requests for materials should be addressed to L.M. (E-mail: laurent.maron@irsamc.ups-tlse.fr) or to C.Z. (E-mail: zcq@nju.edu.cn).

Supplementary Methods

1. Experimental Procedure: All manipulations were performed under an atmosphere of argon or nitrogen using standard Schlenk techniques or in a glovebox. Commercially available chemicals were used as received without further purification. The solvents were obtained by passing through a Solve Purer G5 (MIKROUNA) solvent purification system and further dried over 4 Å molecular sieves. THF-d_8 was dried over Na/K and stored under an Ar or N_2 atmosphere prior to use. Nuclear magnetic resonance spectroscopy was performed using a Bruker AVIII-400 (^{1}H 400 MHz; ^{13}C{^{1}H} 101 MHz; ^{31}P{^{1}H} 162 MHz) spectrometer at room temperature (RT). The ^{1}H and ^{13}C{^{1}H} NMR chemical shifts (δ) are relative to tetramethylsilane, and ^{31}P{^{1}H} NMR chemical shifts are relative to 85% H_3PO_4. Absolute values of the coupling constants are provided in Hertz (Hz). Multiplicities are abbreviated as singlet (s), doublet (d), triplet (t), multiplet (m), and broad (br). Magnetic susceptibility measurements on crystalline samples were performed using a Quantum Design SQUID VSM magnetometer from 300 to 1.8 K under an external magnetic field of 1000 Oe. The sample was added to a pre-weighed SQUID capsule in a glovebox. The capsule was then sealed, weighed, and transferred to the SQUID cavity for the magnetic measurement. All magnetic data were corrected for the diamagnetic contributions of the sample holder and of the core diamagnetism of the samples using Pascal's constant.[1] Elemental analyses (C, H, N) were performed on a Vario EL III elemental analyzer at the Shanghai Institute of Organic Chemistry, Chinese Academy of Sciences. The ligand [$CH_3N(CH_2CH_2NHP^iPr_2)_2$] and complex **1** were prepared according to previously reported procedure.[2]

Synthesis of ligand [$CH_3N(CH_2CH_2NHP^iPr_2)_2$]: A solution of $^iPr_2PCl$ (3.06 g, 20.0 mmol, 2 equiv.) in THF (20 mL) was added dropwise to a solution of $CH_3N(CH_2CH_2NH_2)_2$ (1.17 g, 10.0 mmol, 1

equiv.) and Et_3N (11 mL, 80.0 mmol, 8 equiv.) in THF (30 mL), resulting in the immediate formation of a white precipitate. The mixture was stirred overnight then dried *in vacuo*. The white solid was extracted with hexane and filtered through celite. The volatile components were removed under reduced pressure to give ligand [$CH_3N(CH_2CH_2NHP^iPr_2)_2$] as a colorless oil. Yield: 3.14 g (90 %). ^{1}H NMR (C_6D_6, 400 MHz, ppm) δ 2.92-2.98 (m, 4H, NCH_2CH_2), 2.25 (t, $^3J_{HH}$ = 6.4 Hz, 4H, NCH_2CH_2), 2.02 (s, 3H, NCH_3), 1.44-1.55 (m, 4H, $CH(CH_3)_2$), 1.31-1.33 (m, 2H, N*H*), 1.00-1.06 (m, 24H, $CH(CH_3)_2$). $^{13}C\{^1H\}$ NMR (C_6D_6, 101 MHz, ppm) δ 60.5 (NCH_2CH_2), 60.4 (NCH_2CH_2), 46.2 (NCH_2CH_2), 45.9 (NCH_2CH_2), 41.6 (CH_3N), 26.5 ($CH(CH_3)_2$), 26.4 ($CH(CH_3)_2$) 19.2 ($CH(CH_3)_2$), 19.0 ($CH(CH_3)_2$), 17.4 ($CH(CH_3)_2$), 17.3 ($CH(CH_3)_2$). $^{31}P\{^1H\}$ NMR (C_6D_6, 162 MHz, ppm) δ 63.6. HRMS (ESI) calcd for $C_{17}H_{41}N_3P_2$ $[M+H]^+$ 350.2848, found 350.2872.

Synthesis of complex 1: A 2.4 M solution of *n*-BuLi in hexane (1.68 mL, 4.0 mmol, 2 equiv.) was added dropwise to a solution of ligand [$CH_3N(CH_2CH_2NHP^iPr_2)_2$] (700 mg, 2.0 mmol, 1 equiv.) in THF (20 mL) at -30 °C. The mixture was allowed to warm to RT and stirred for a further 2 h, and was then added to the solution of UCl_4 (760 mg, 2.0 mmol, 1 equiv.) in THF (20 mL). The mixture was stirred overnight at RT and then the solvents were removed under reduced pressure and the residues were extracted with toluene. The filtrate was dried *in vacuo* to afford complex **1** as a pure brown solid (1.19 g, 82 %). Crystals of **1** suitable for X-ray diffraction were grown from a saturated solution in toluene stored at -30 °C. ^{1}H NMR (C_6D_6, 400 MHz, ppm) δ 88.49 (s, 2H, CH_2), 72.02 (s, 2H, CH_2), 69.15 (s, 2H, CH_2), 58.14 (s, 2H, CH_2), 32.55 (s, 6H, $CH(CH_3)_2$), 32.03 (s, 6H, $CH(CH_3)_2$), 29.00 (s, 6H, $CH(CH_3)_2$), 25.90 (s, 6H, $CH(CH_3)_2$), -21.25 (s, 4H, THF), -36.13 (s, 2H, $CH(CH_3)_2$), -52.90 (s, 4H, THF), -70.89 (s, 2H, $CH(CH_3)_2$), -81.53 (s, 3H, NCH_3). $^{13}C\{^1H\}$ NMR (C_6D_6, 101 MHz, ppm) δ 30.7, 29.0, 21.8, 13.1, 0.2. $^{31}P\{^1H\}$ NMR (C_6D_6, 162 MHz, ppm) not observed from

the range of +1000 to -1000 ppm. Anal. Calcd. for $C_{21}H_{47}Cl_2N_3OP_2U$: C, 34.62; H, 6.50; N, 5.77. Found: C, 34.42; H, 6.47; N, 5.83.

Protonation of complex 4a with acid: An excess of pyridine hydrochloride (10.7 mg, 0.093 mmol, 25 equiv.) was added to a brown solution of **4a** (5.0 mg, 0.0037 mmol, 1 equiv.) in THF (1 ml). The mixture was stirred at RT overnight, affording a pale-yellow solution and a white precipitate. The supernatant was removed and the solid was washed three times with 1.0 mL of THF. The solid was dried under vacuum. The amount of ammonia was evaluated by quantitative ^{1}H NMR with dibromomethane as an internal standard. The NH_4Cl was formed in 73% yield (Supplementary Fig. 14).

Protonation of complex 4b with acid: An excess of pyridine hydrochloride (9.0 mg, 0.078 mmol, 25 equiv.) was added to a brown solution of **4b** (5.0 mg, 0.0031 mmol, 1 equiv.) in THF (1 ml). The mixture was stirred at RT overnight, affording a pale-yellow solution with a white precipitate. The supernatant was removed and the solid was washed three times with 1.0 mL of THF. The solid was dried under vacuum. The amount of ammonia was evaluated by quantitative ^{1}H NMR with dibromomethane as an internal standard. The NH_4Cl was formed in 82% yield (Supplementary Fig. 15).

Protonation of complex 5a with acid: An excess of pyridine hydrochloride (22.5 mg, 0.13 mmol, 50 equiv.) was added to a brown solution of **5a** (5.0 mg, 0.0039 mmol, 1 equiv.) in THF (1 ml). The mixture was stirred at RT overnight, affording a pale-yellow solution with a white precipitate. The supernatant was removed and the solid was washed three times with 1.0 mL of THF. The solid was dried under vacuum. The amount of ammonia was measured by quantitative ^{1}H NMR with dibromomethane as an internal standard. The NH_4Cl was formed in 75% yield (Supplementary Fig.

16).

Protonation of complex 5b with acid: An excess of pyridine hydrochloride (18.6 mg, 0.13 mmol, 50 equiv.) was added to a brown solution of **5b** (5.0 mg, 0.0032 mmol, 1 equiv.) in THF (1 ml). The mixture was stirred at RT overnight, affording a pale-yellow solution with a white precipitate. The supernatant was removed and the solid was washed three times with 1.0 mL of THF. The solid was dried under vacuum. The amount of ammonia was measured by quantitative ^{1}H NMR with dibromomethane as an internal standard. The NH_4Cl was formed in 68% yield (Supplementary Fig. 17).

Reaction of complex 4a with H_2 and PyHCl: In an NMR tube, a brown solution of **4a** (5.0 mg, 0.0037 mmol, 1 equiv.) in 0.5 ml of THF-d_8 was frozen and degassed three times then exposed to 1 atm of H_2 at RT. The NMR tube was closed and left at RT for 24 h, then all the volatiles were vacuum transferred into a Schlenk flask containing a PyHCl (10.7 mg, 0.093 mmol, 25 equiv.) solution in DMSO-d_6. No NH_4Cl was detected. An excess of pyridine hydrochloride (10.7 mg, 0.093 mmol, 25 equiv.) in THF was added to the residue remaining in the NMR tube. The mixture was stirred at RT overnight, affording a pale-yellow solution with a white precipitate. The supernatant was removed and the solid was washed three times with 1.0 mL of THF. The solid was dried under vacuum. The amount of ammonia (58%) was measured by quantitative ^{1}H NMR spectrum with dibromomethane as an internal standard (Supplementary Fig. 18).

Reaction of complex 4b with H_2 and PyHCl: In an NMR tube, a brown solution of **4b** (5.0 mg, 0.0031 mmol, 1 equiv.) in 0.5 ml of THF-d_8 was frozen and degassed three times and exposed to 1 atm of H_2 at RT. The NMR tube was closed and left at RT for 24 h, then all the volatiles were vacuum transferred into a Schlenk flask containing a PyHCl (9.0 mg, 0.078 mmol, 25 equiv.) solution in

DMSO-d_6. No NH_4Cl was detected. An excess of pyridine hydrochloride (9.0 mg, 0.078 mmol, 25 equiv.) in THF was added to the residue left in the NMR tube. The mixture was stirred at RT overnight, affording a pale-yellow solution with a white precipitate. The supernatant was removed and the solid was washed three times with 1.0 mL of THF. The solid was dried under vacuum. The amount of ammonia (67%) was measured by quantitative ^1H NMR spectrum with dibromomethane as an internal standard (Supplementary Fig. 19).

Reaction of complex 5a with H_2 and PyHCl: In an NMR tube, a brown solution of **5a** (5.0 mg, 0.0039 mmol, 1 equiv.) in 0.5 ml of THF-d_8 was frozen and degassed three times and exposed to 1 atm of H_2 at RT. The NMR tube was closed and left at RT for 24 h, then all the volatiles were vacuum transferred into a Schlenk flask containing a PyHCl (22.5 mg, 0.13 mmol, 50 equiv.) solution in DMSO-d_6. No NH_4Cl was detected. An excess of pyridine hydrochloride (22.5 mg, 0.13 mmol, 50 equiv.) in THF was added to the residue left in the NMR tube. The mixture was stirred at RT overnight, affording a pale-yellow solution with a white precipitate. The supernatant was removed and the solid was washed three times with 1.0 mL of THF. The solid was dried under vacuum. The amount of ammonia (54%) was measured by quantitative ^1H NMR spectrum with dibromomethane as an internal standard (Supplementary Fig. 20).

Reaction of complex 5b with H_2 and PyHCl: In an NMR tube, a brown solution of **5b** (5.0 mg, 0.0032 mmol, 1 equiv.) in 0.5 ml of THF-d_8 was frozen and degassed three times and exposed to 1 atm of H_2 at RT. The NMR tube was closed and left at RT for 24 h, then all the volatiles were vacuum transferred into a Schlenk flask containing a PyHCl (18.6 mg, 0.13 mmol, 50 equiv.) solution in DMSO-d_6. No NH_4Cl was detected. An excess of pyridine hydrochloride (18.6 mg, 0.13 mmol, 50 equiv.) in THF was added to the residue left in the NMR tube. The mixture was stirred at RT

overnight, affording a pale-yellow solution with a white precipitate. The supernatant was removed and the solid was washed three times with 1.0 mL of THF. The solid was dried under vacuum. The amount of ammonia (41%) was measured by quantitative ^{1}H NMR spectrum with dibromomethane as an internal standard (Supplementary Fig. 21).

2. X-ray crystallographic analysis: The crystallographic data were collected using a Bruker APEX-II CCD area detector with a radiation source of Ga(Kα) (1.34139 Å) or Mo(Kα) (0.71073 Å). Multi-scan or empirical absorption corrections (SADABS) were applied. The structures were solved using Patterson methods, expanded using difference Fourier syntheses, and refined using full-matrix least squares fitting on F^2 using the Bruker SHELXTL-2014 program package.[3,4] All non-hydrogen atoms were refined anisotropically. Hydrogen atoms were introduced at their geometric positions and refined as riding atoms. In complexes **3a** and **3b**, the restraint (SIMU) was employed to refine the disordered N atoms. In complexes **4a** and **4b,** pseudo-isotropic (ISOR) and SIMU restraints were applied for the refinement of the carbon atoms of solvent molecules (toluene). In complexes **5a** and **5b**, the restraint (SIMU) was used to refine the carbon atoms of cyclooctadiene groups. These data can be obtained free of charge from the Cambridge Crystallographic Data Centre (www.ccdc.cam.ac.uk/data-request/cif). Details regarding the data collection and refinement for these complexes are given in Supplementary Table 1.

3. Theoretical Calculations: All calculations were performed using Gaussian09 suite of programs[5] using the Becke's 3-parameter hybrid functional[6] combined with the non-local correlation functional provided by Perdew/Wang.[7] The U, Ir, Rh, Cl and P atoms were represented with a small-core Stuttgart-Dresden relativistic effective core potential associated with their adapted basis set.[8-10] Additionally, the P and Cl basis set were augmented by a d-polarization function (α_P= 0.340 and α_C = 0.632)[11] to represent the valence orbitals. All the other atoms C, N and H were described with a 6-31G (d,p), double –ζ quality basis set.[12,13] The full geometry optimization of all complexes were carried out in the gas phase without any symmetry constraints. The enthalpy energy was computed at T = 298 K in the gas phase within the harmonic approximation. NBO analysis were carried out with three different version of the NBO program, namely NBO 3.1 (include in Gaussian), NBO 5.0 and NBO 6.0, to ensure the validity of the analysis.

Supplementary Figures and Tables

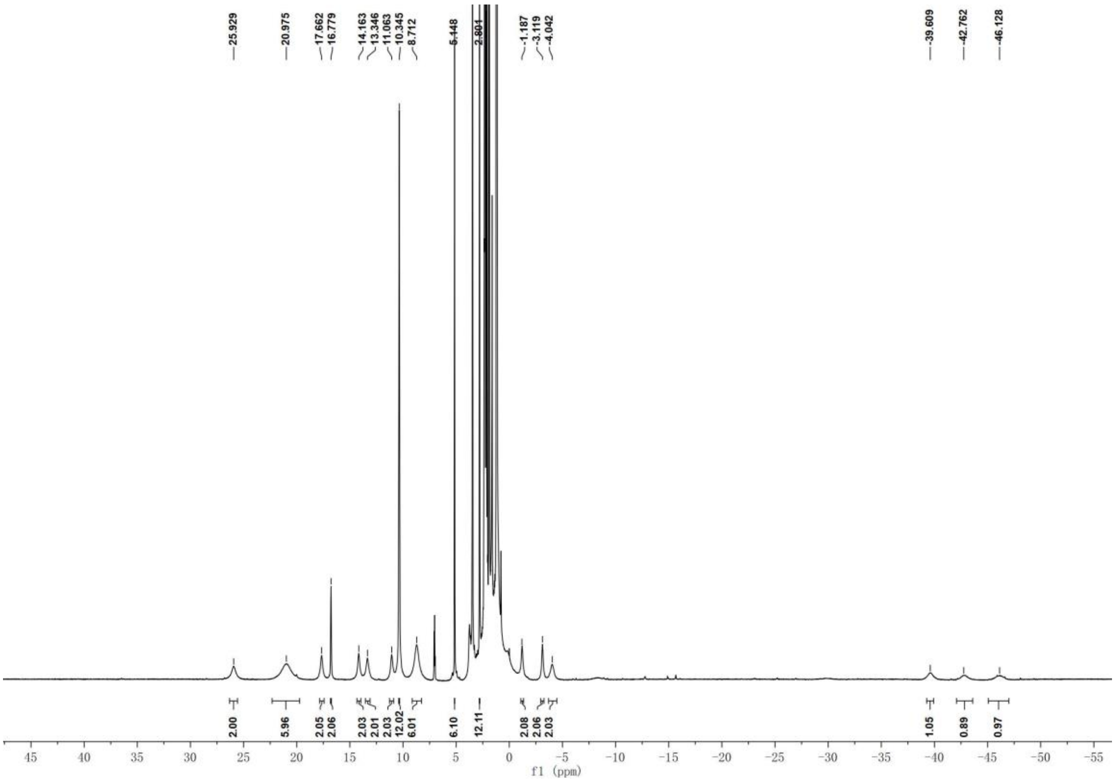

Supplementary Figure 1. The ^{1}H NMR (THF-d_8, 400 MHz) spectrum of complex **2a**.

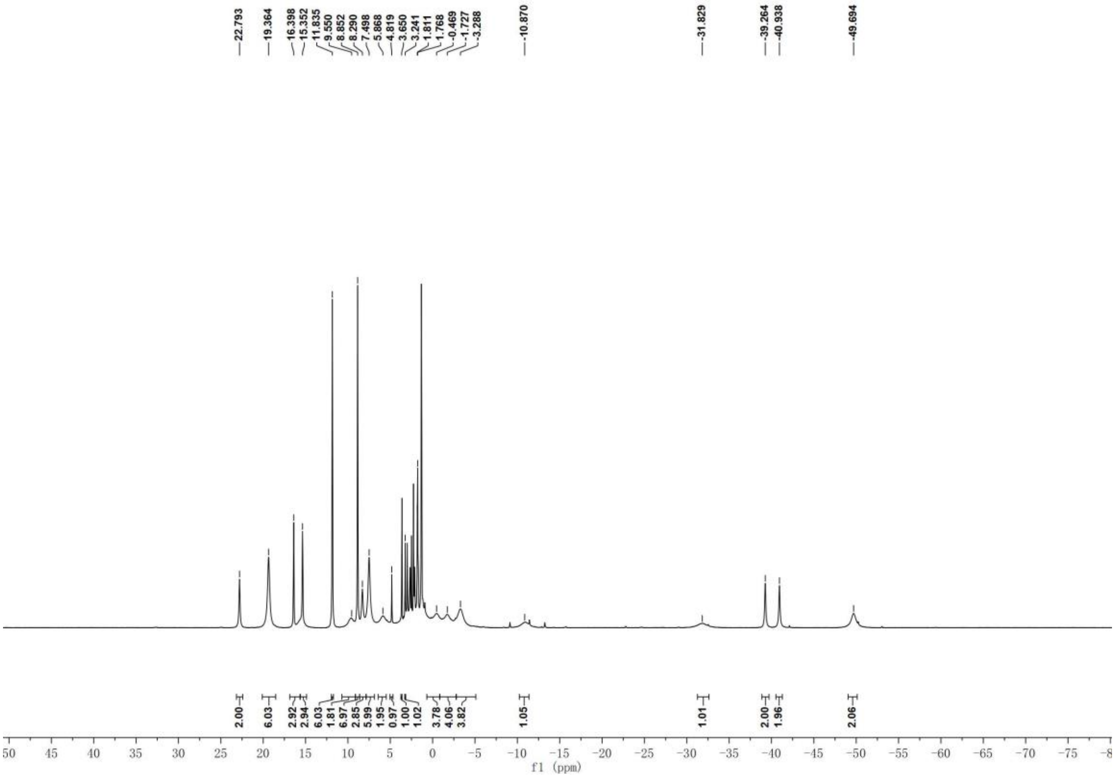

Supplementary Figure 2. The ^{1}H NMR (THF-d_8, 400 MHz) spectrum of complex **2b**.

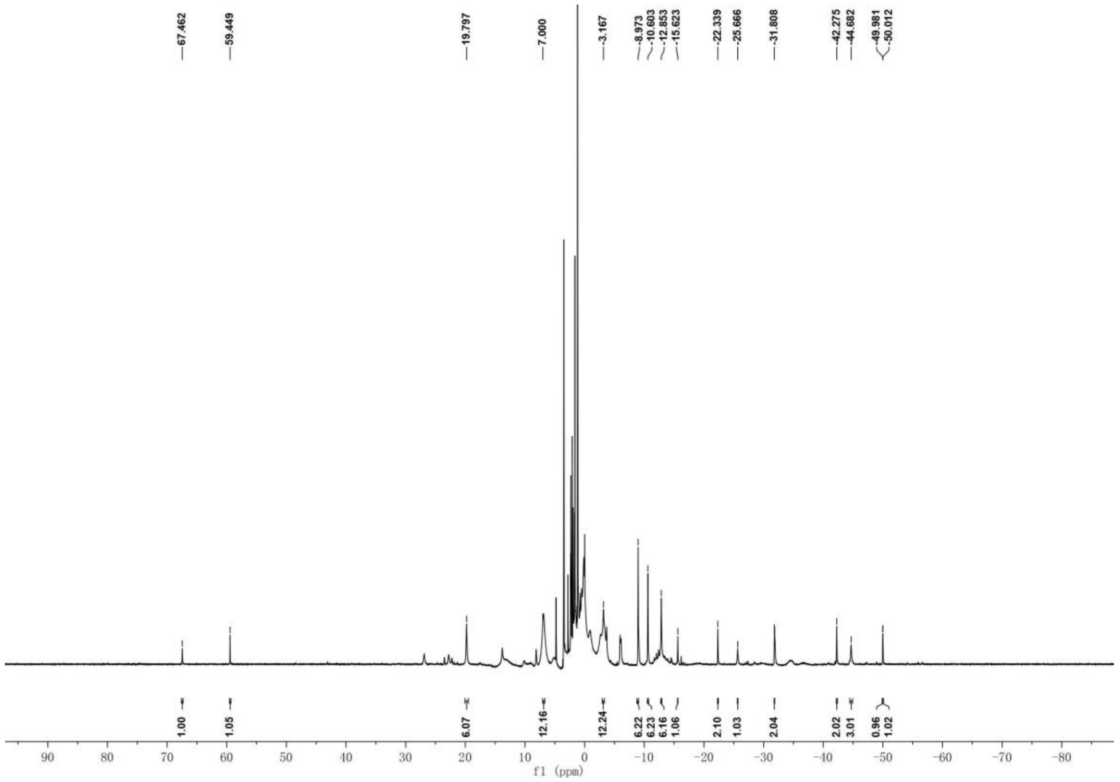

Supplementary Figure 3. The ^{1}H NMR (THF-d_8, 400 MHz) spectrum of complex **3a**.

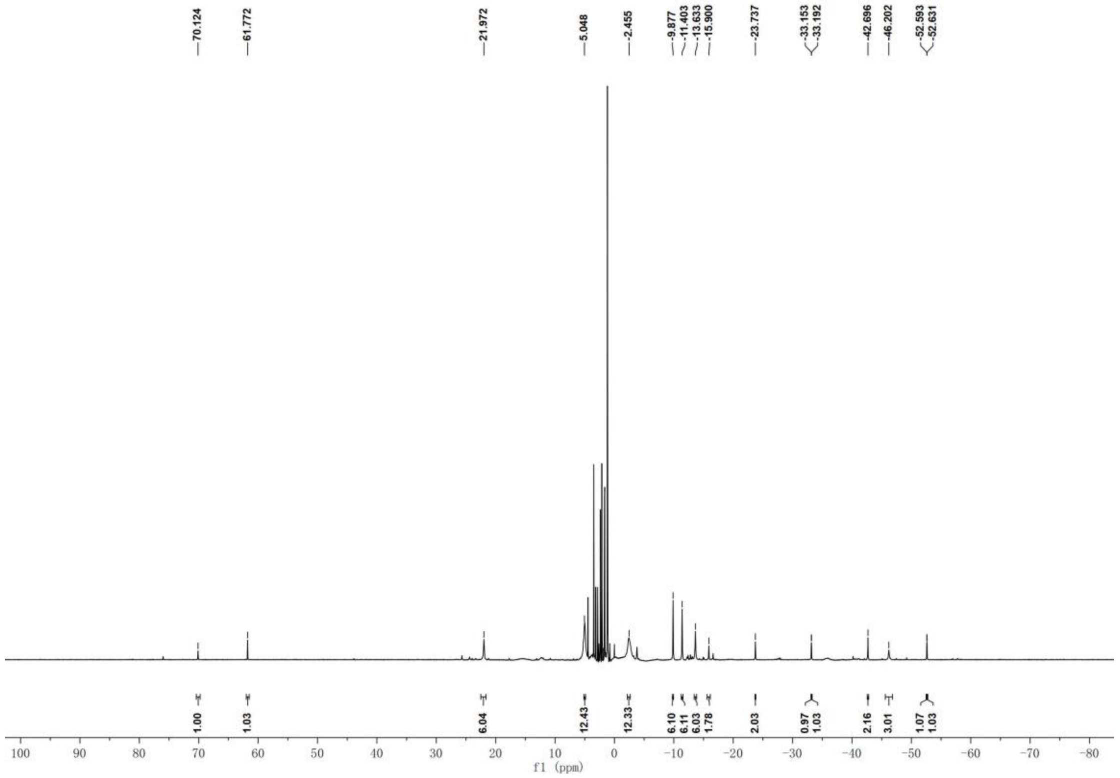

Supplementary Figure 4. The ^{1}H NMR (THF-d_8, 400 MHz) spectrum of complex **3b**.

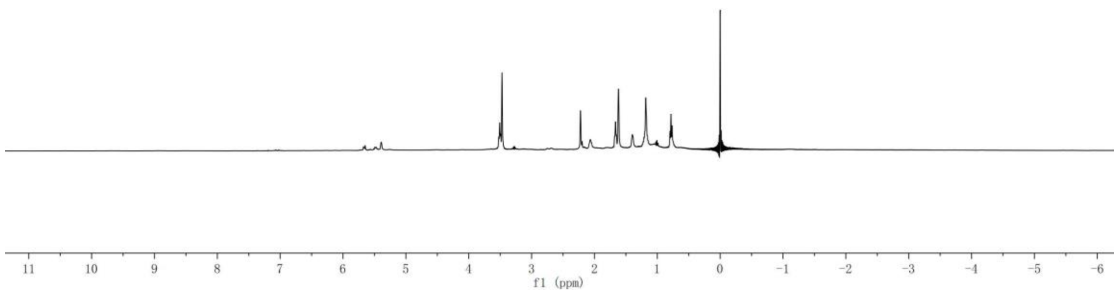

Supplementary Figure 5. The ¹H NMR (THF-d_8, 400 MHz) spectrum of the *in-situ* reaction of **3a** with 4 equiv. of KC_8.

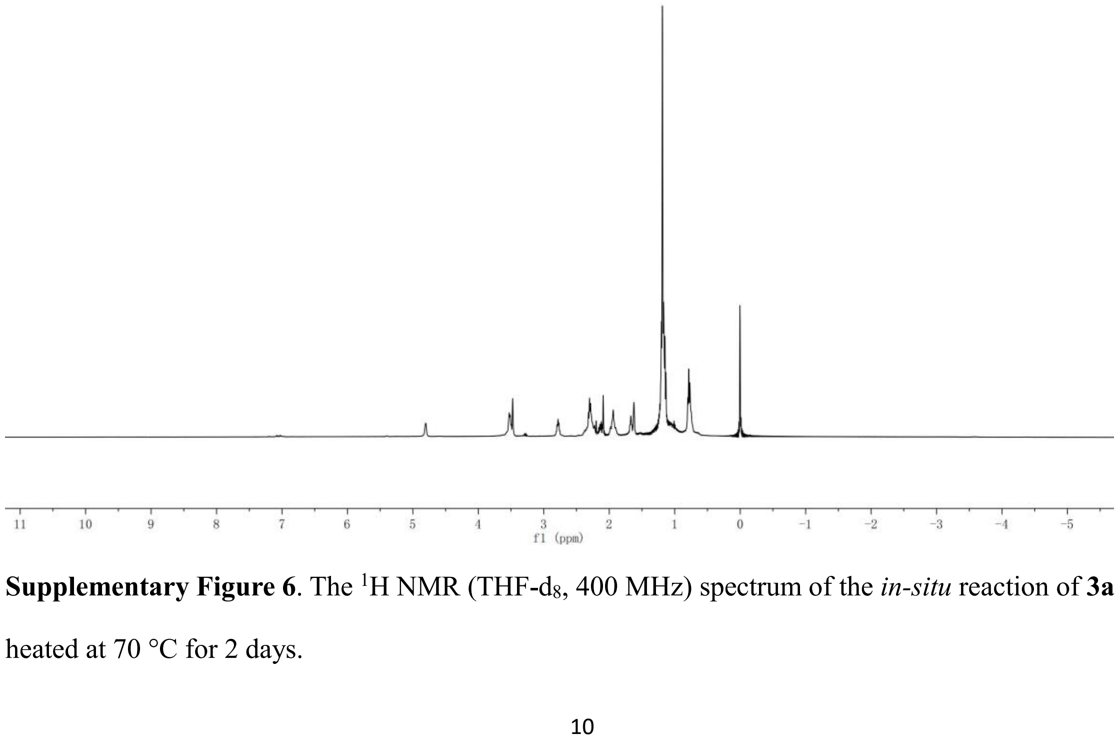

Supplementary Figure 6. The ¹H NMR (THF-d_8, 400 MHz) spectrum of the *in-situ* reaction of **3a** heated at 70 °C for 2 days.

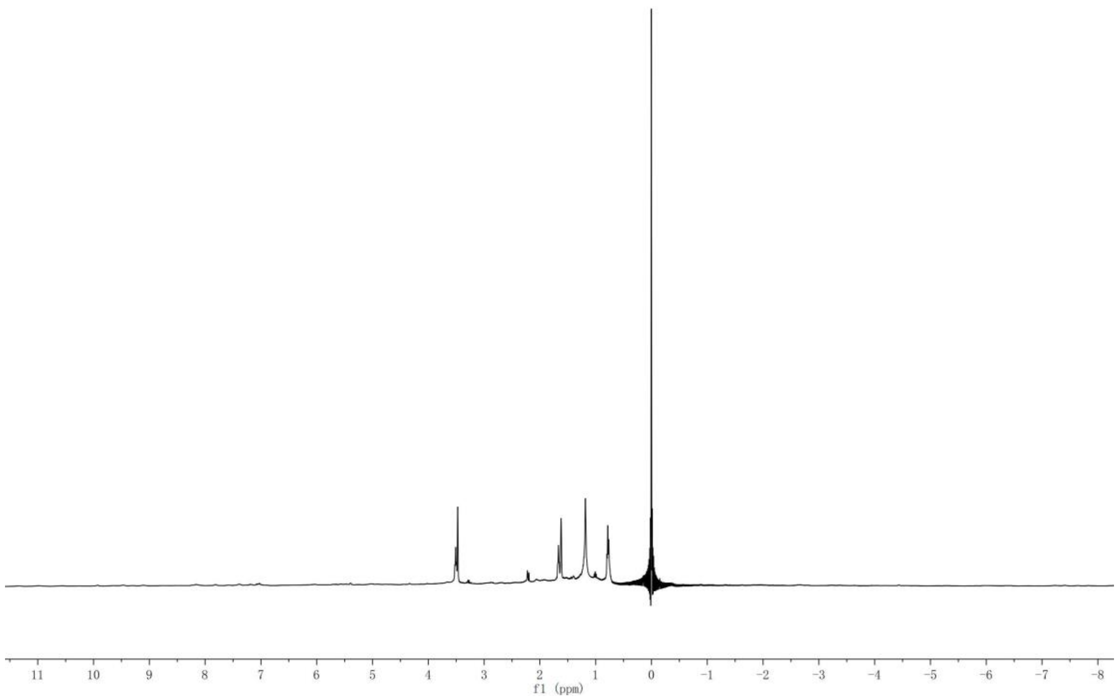

Supplementary Figure 7. The ^{1}H NMR (THF-d_8, 400 MHz) spectrum of the *in-situ* reaction of **3b** with 4 equiv. of KC_8.

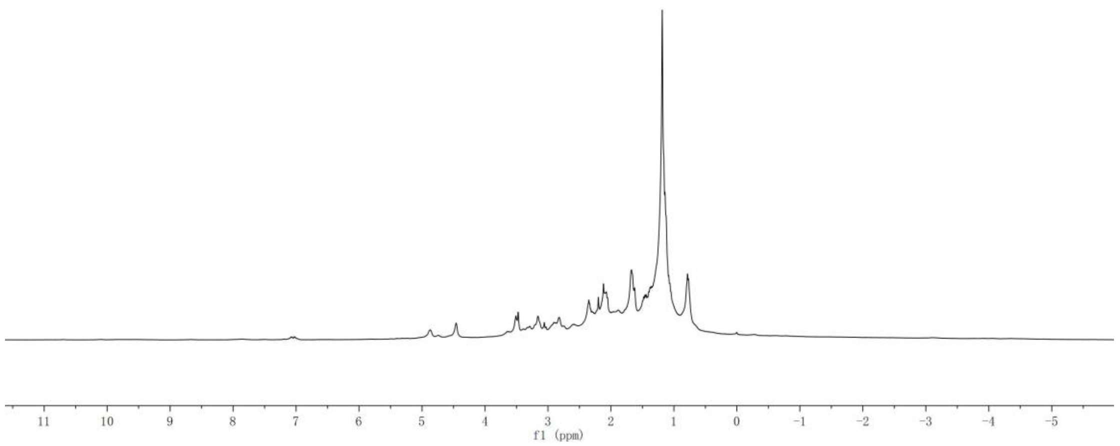

Supplementary Figure 8. The ^{1}H NMR (THF-d_8, 400 MHz) spectrum of the *in-situ* reaction of **3b** heated at 70 °C for 2 days.

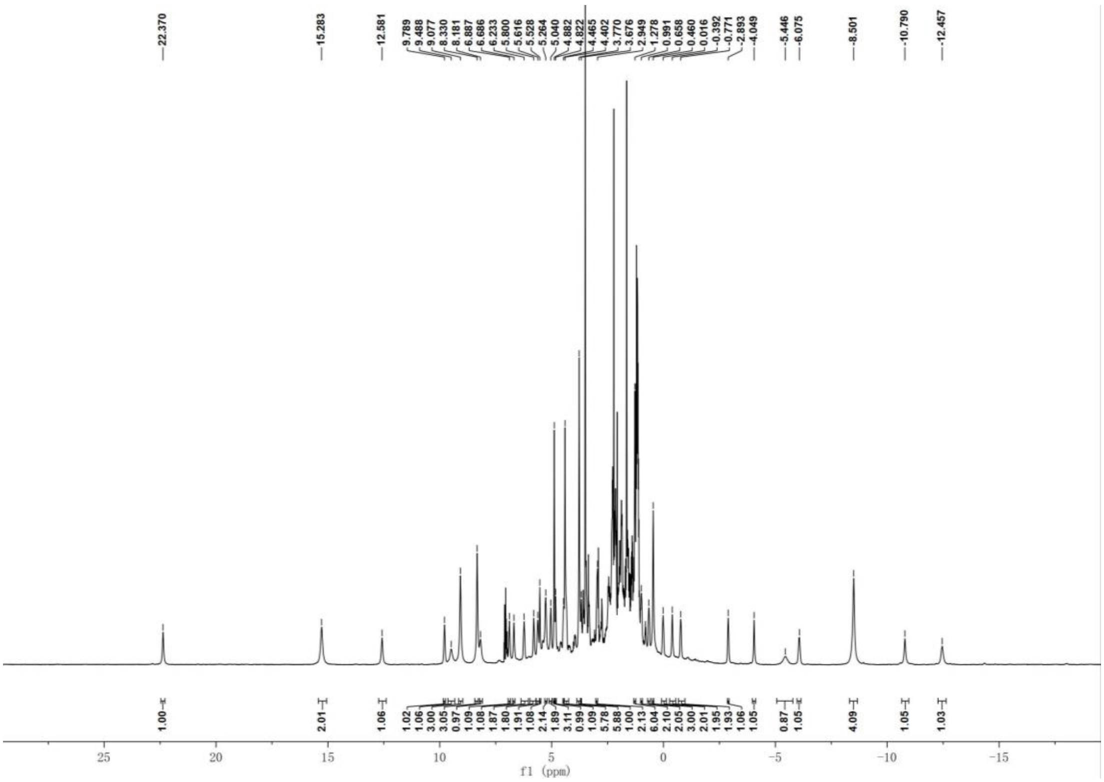

Supplementary Figure 9. The ^{1}H NMR (THF-d_8, 400 MHz) spectrum of complex **4a**.

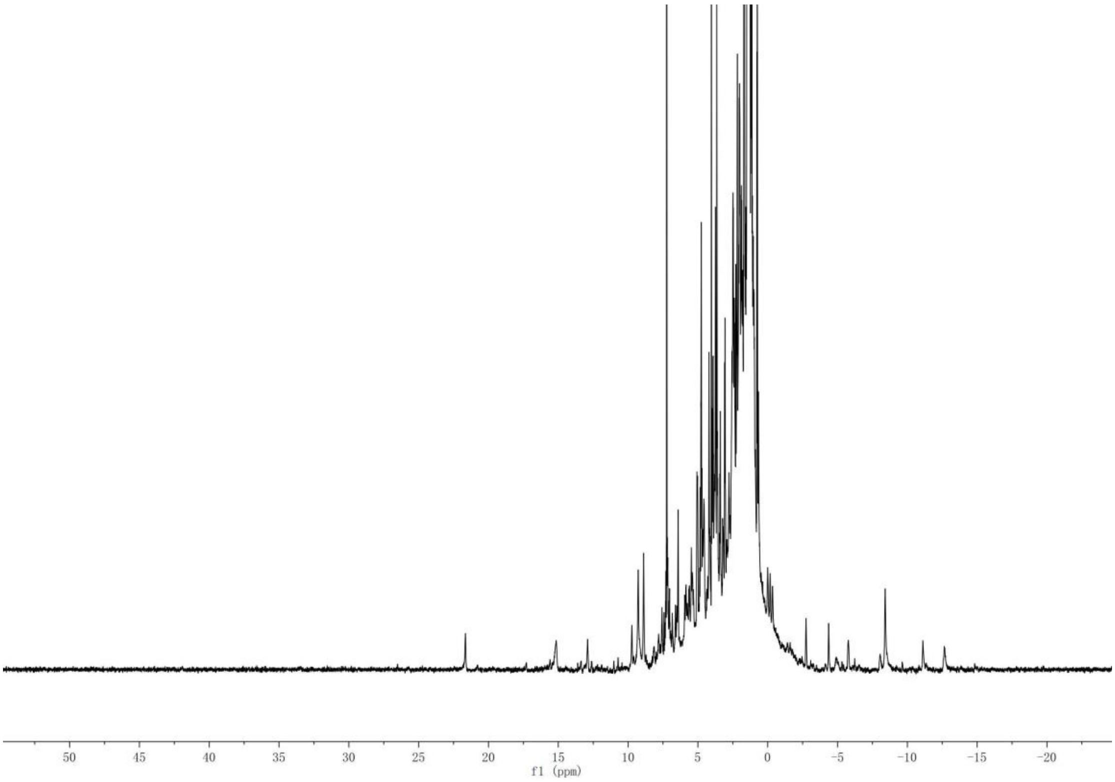

Supplementary Figure 10. The ^{1}H NMR (THF-d_8, 400 MHz) spectrum of the *in-situ* reaction of **3a** under UV light irradiation. No paramagnetic uranium complexes except **4a** were observed.

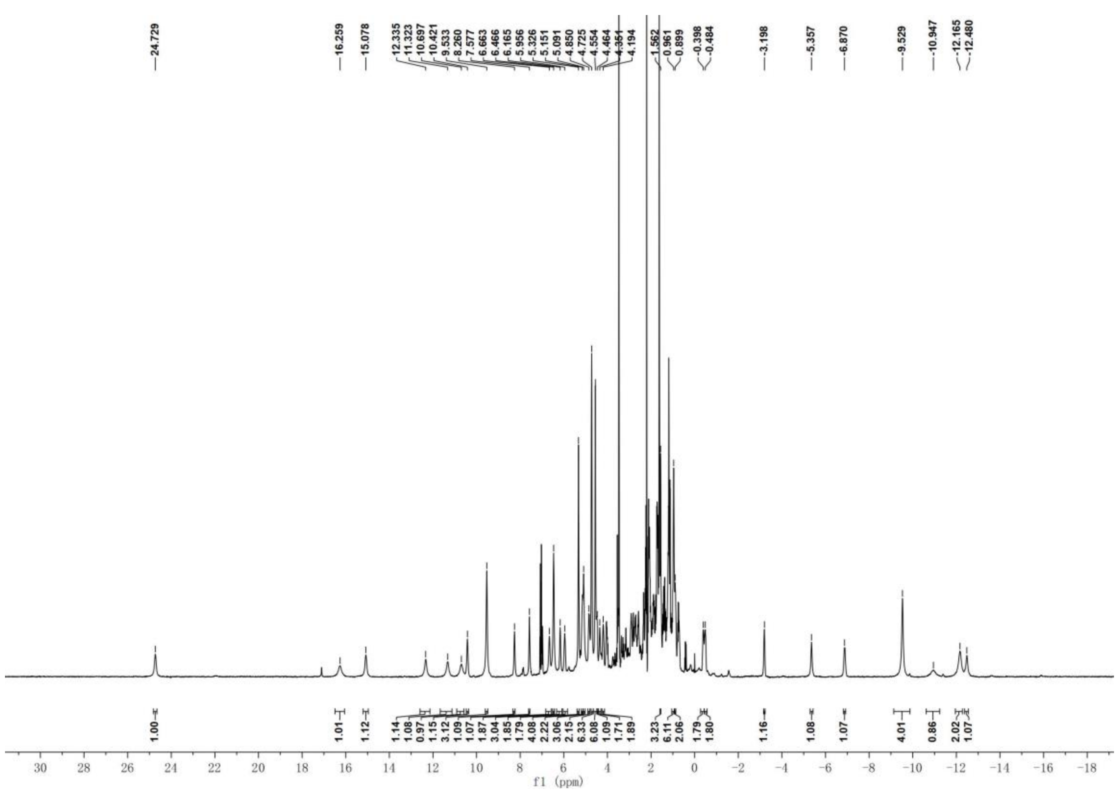

Supplementary Figure 11. The ^{1}H NMR (THF-d_8, 400 MHz) spectrum of complex **4b**.

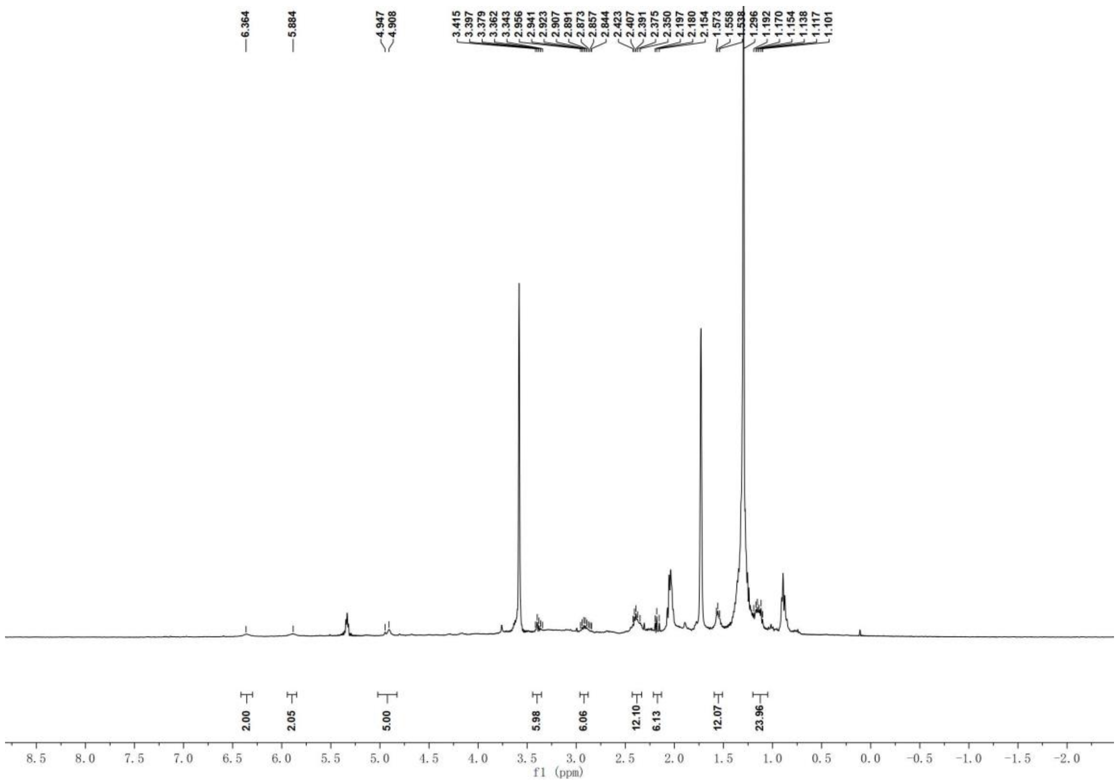

Supplementary Figure 12. The ^{1}H NMR (THF-d_8, 400 MHz) spectrum of complex **5a**. The low concentration of **5a** was due to its poor solubility after crystallized from THF solution.

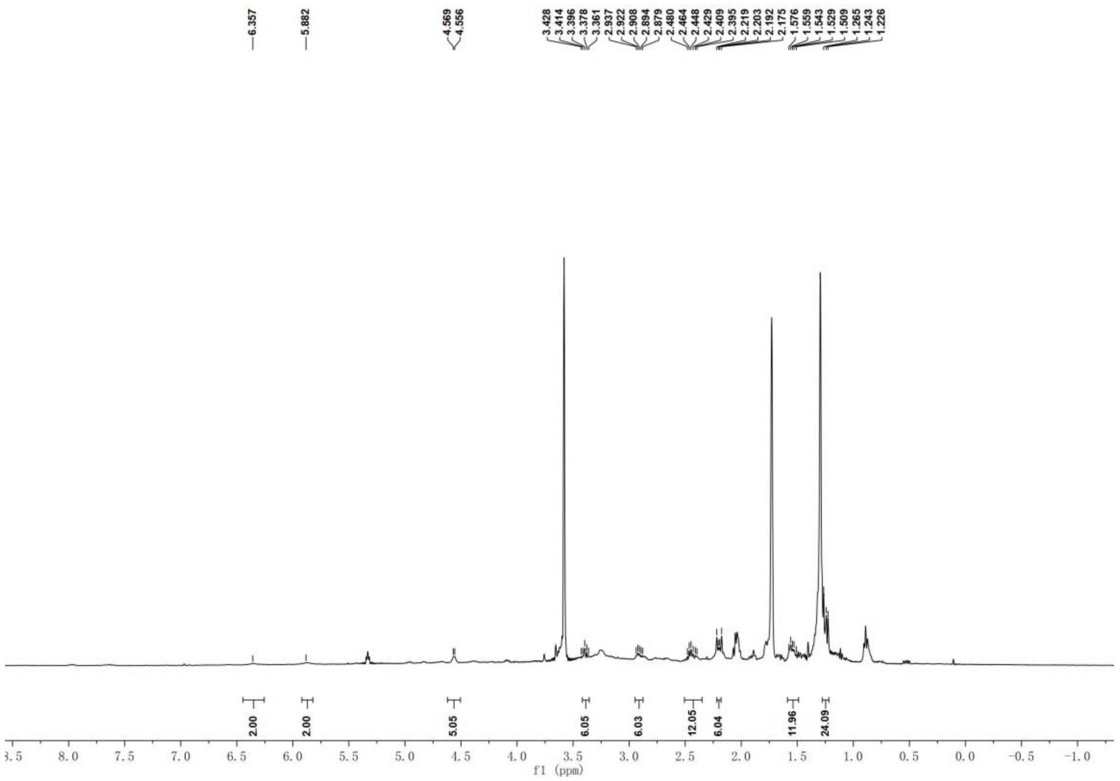

Supplementary Figure 13. The ^{1}H NMR (THF-d_8, 400 MHz) spectrum of complex **5b**. The low concentration of **5b** was due to its poor solubility after crystallized from THF solution.

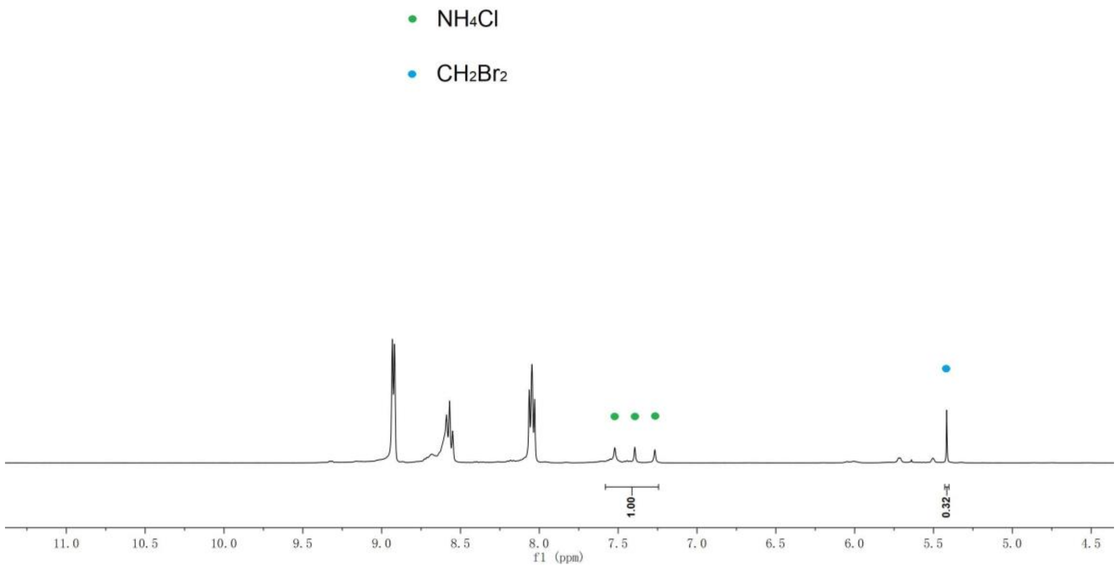

Supplementary Figure 14. The ^{1}H NMR (DMSO-d_6, 400 MHz) spectrum for the reaction of complex **4a** with PyHCl (73% yield of ammonia).

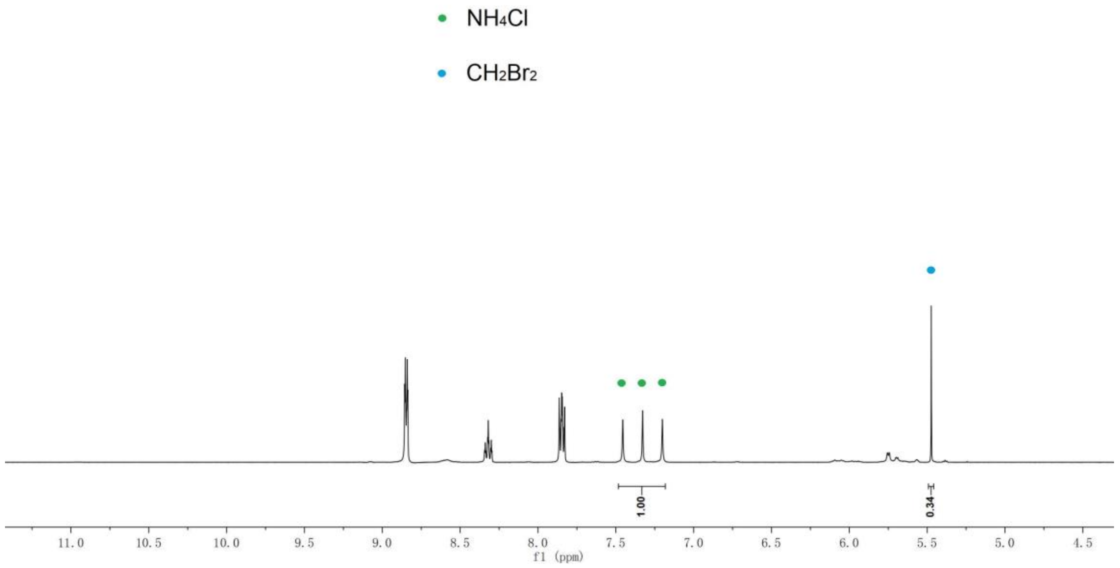

Supplementary Figure 15. The ^{1}H NMR (DMSO-d_6, 400 MHz) spectrum for the reaction of complex **4b** with PyHCl (82% yield of ammonia).

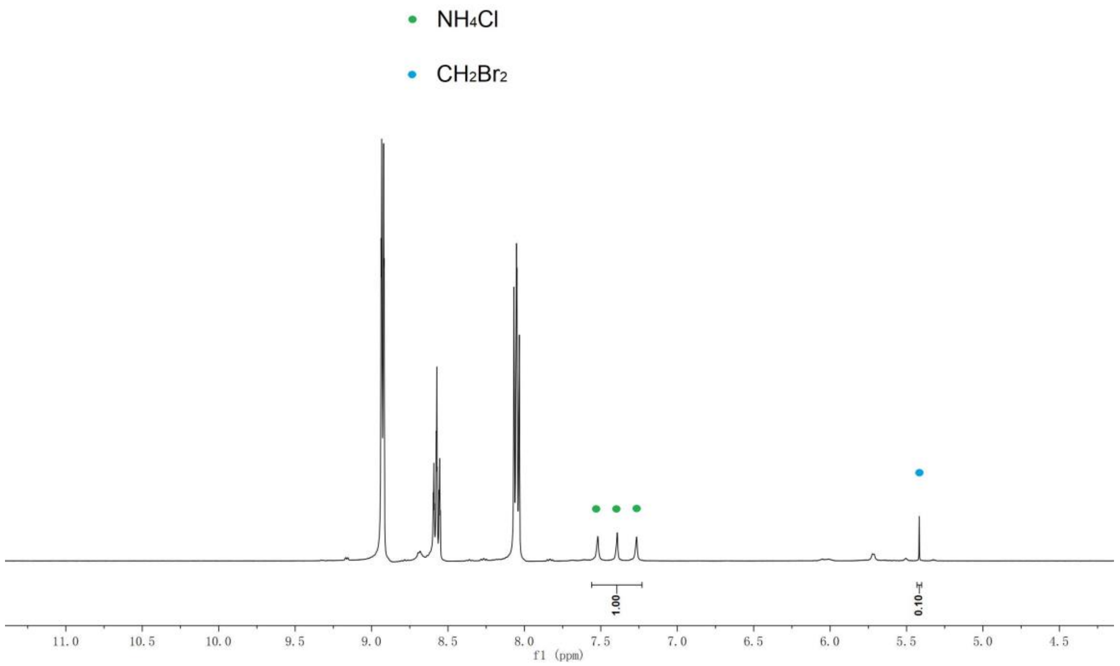

Supplementary Figure 16. The ^{1}H NMR (DMSO-d_6, 400 MHz) spectrum for the reaction of complex **5a** with PyHCl (75% yield of ammonia).

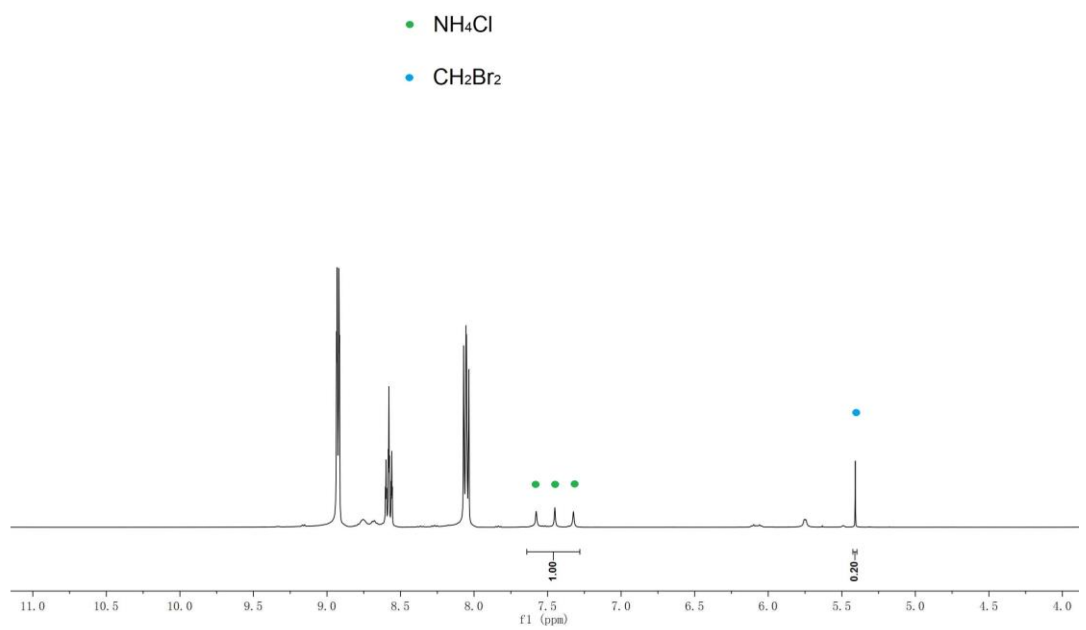

Supplementary Figure 17. The ^{1}H NMR (DMSO-d_6, 400 MHz) spectrum for the reaction of complex **5a** with PyHCl (68% yield of ammonia).

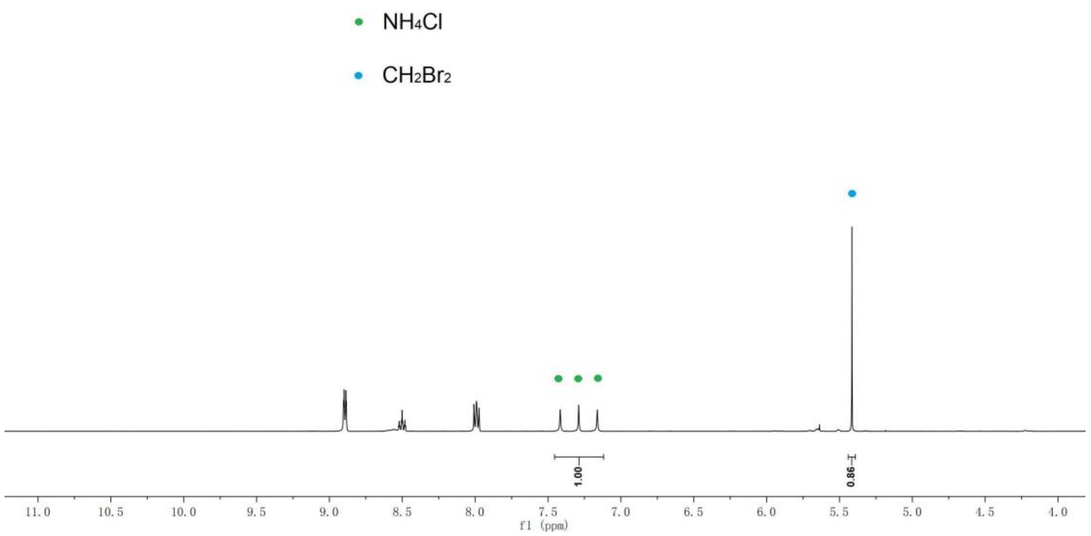

Supplementary Figure 18. The ^{1}H NMR (DMSO-d_6, 400 MHz) spectrum for the reaction of complex **4a** with H_2 after evaporation of the addition of excess PyHCl to the residue (58% yield of ammonia).

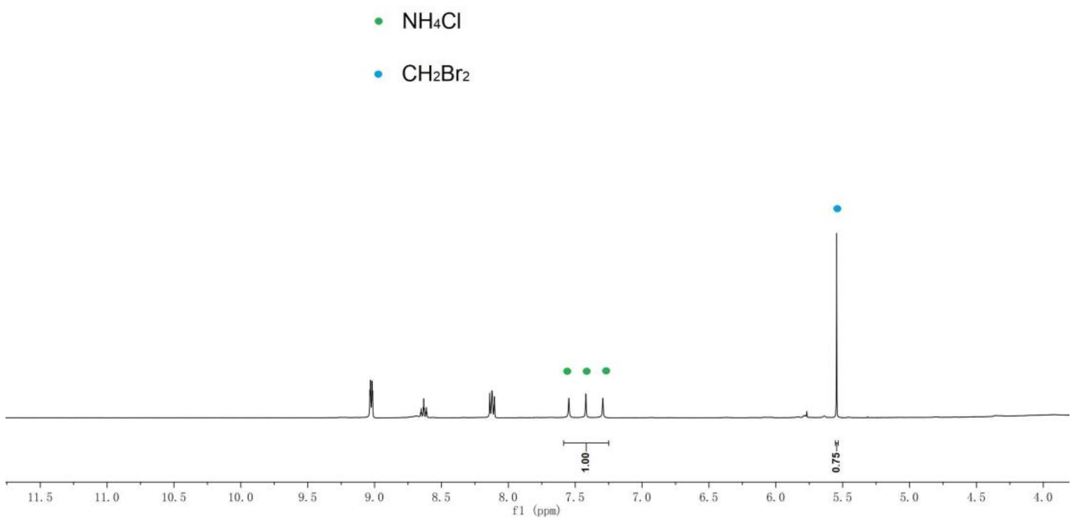

Supplementary Figure 19. The ^{1}H NMR (DMSO-d_6, 400 MHz) spectrum for the reaction of complex **4b** with H_2 after evaporation of the addition of excess PyHCl to the residue (67% yield of ammonia).

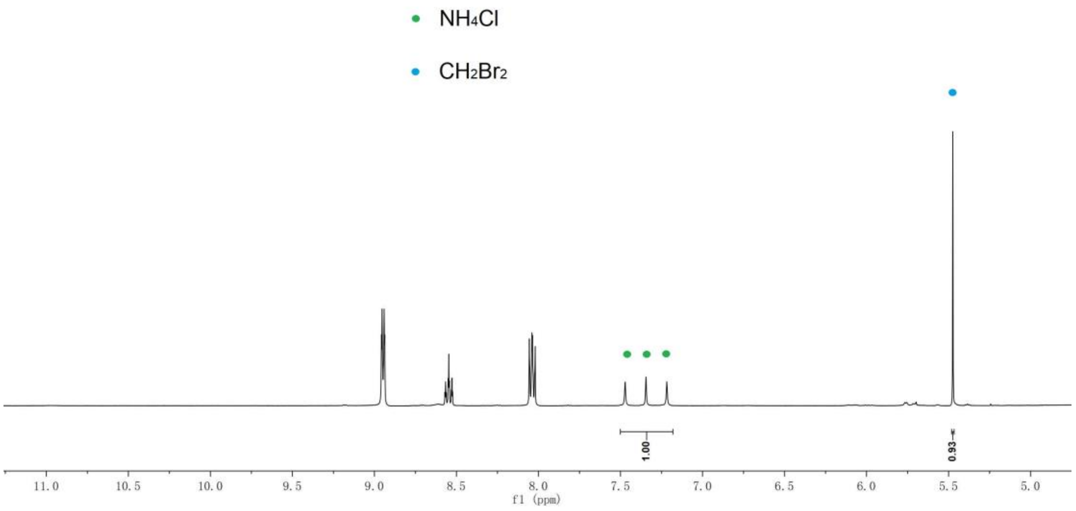

Supplementary Figure 20. The ^{1}H NMR (DMSO-d_6, 400 MHz) spectrum for the reaction of complex **5a** with H_2 after evaporation of the addition of excess PyHCl to the residue (54% yield of ammonia).

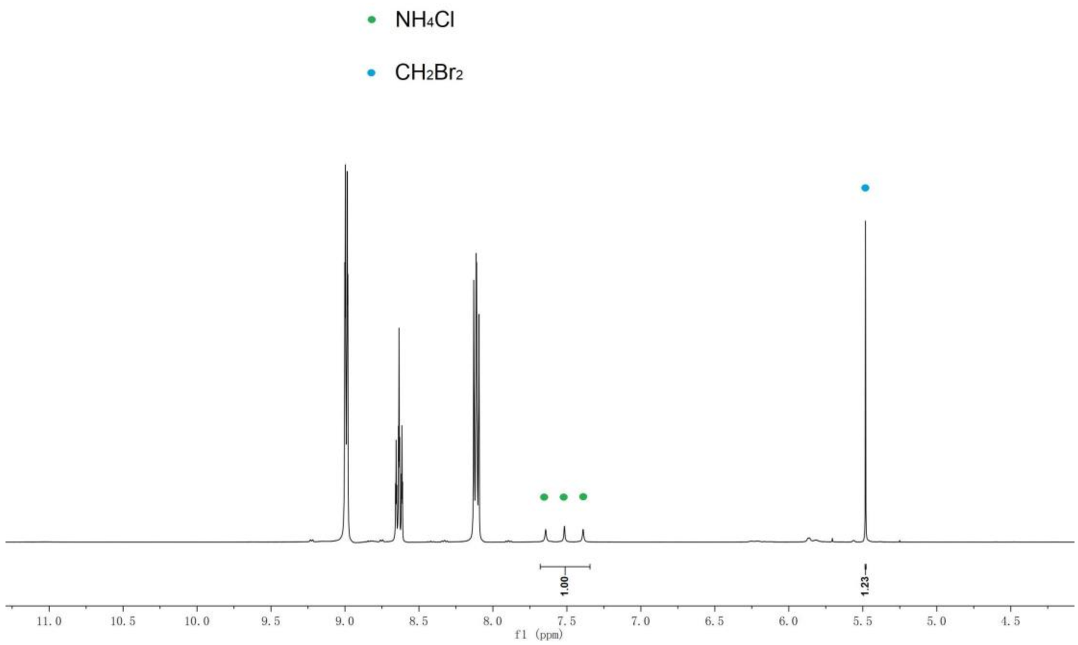

Supplementary Figure 21. The ^{1}H NMR (DMSO-d_6, 400 MHz) spectrum for the reaction of complex **5b** with H_2 after evaporation of the addition of excess PyHCl to the residue (41% yield of ammonia).

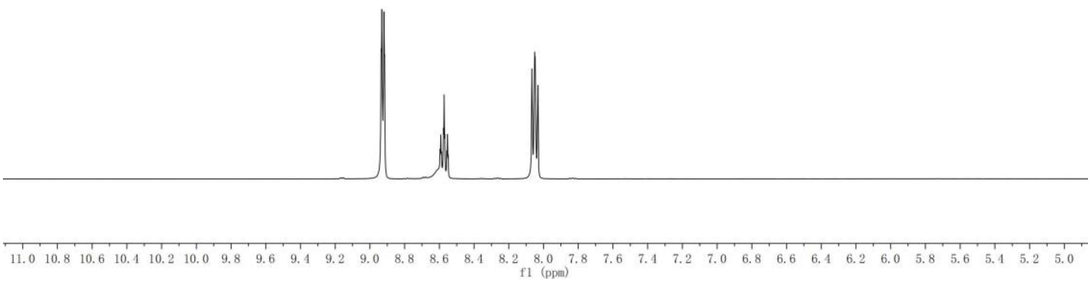

Supplementary Figure 22. The ^{1}H NMR (DMSO-d_6, 400 MHz) spectrum for the reaction of complex **3a** with excess PyHCl. No NH^{4+} was formed in the reaction.

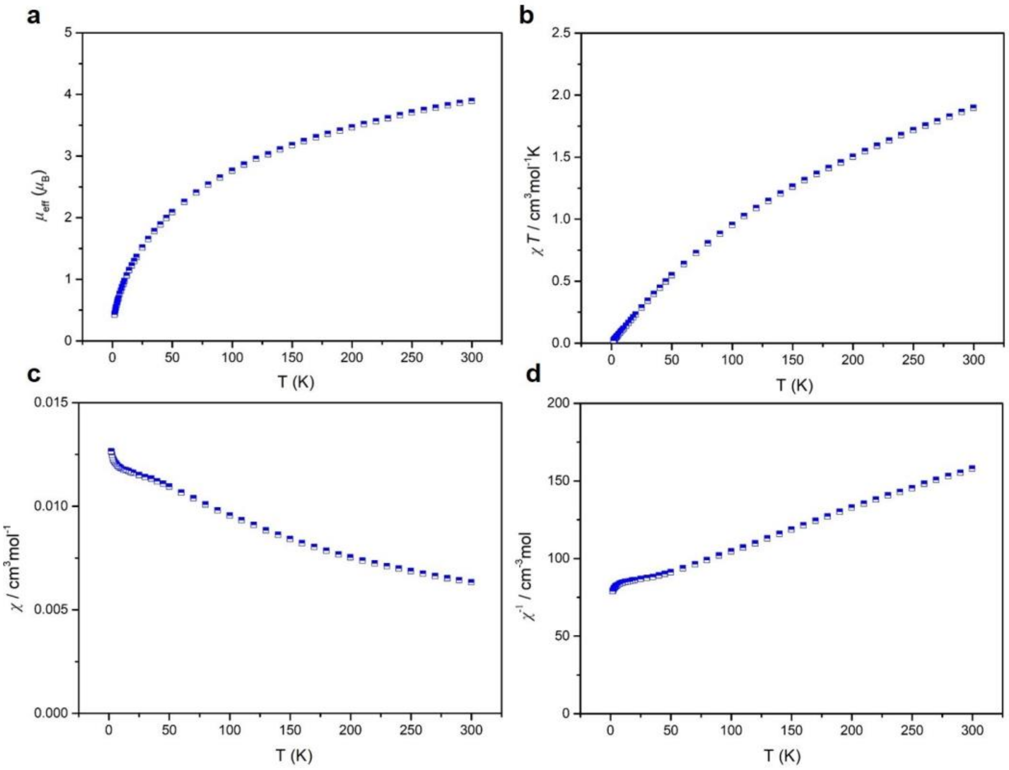

Supplementary Figure 23. Variable-temperature data of **3a**. (a) μ_{eff} vs T, (b) χT vs T, (c) χ vs T, and (d) 1/χ vs T.

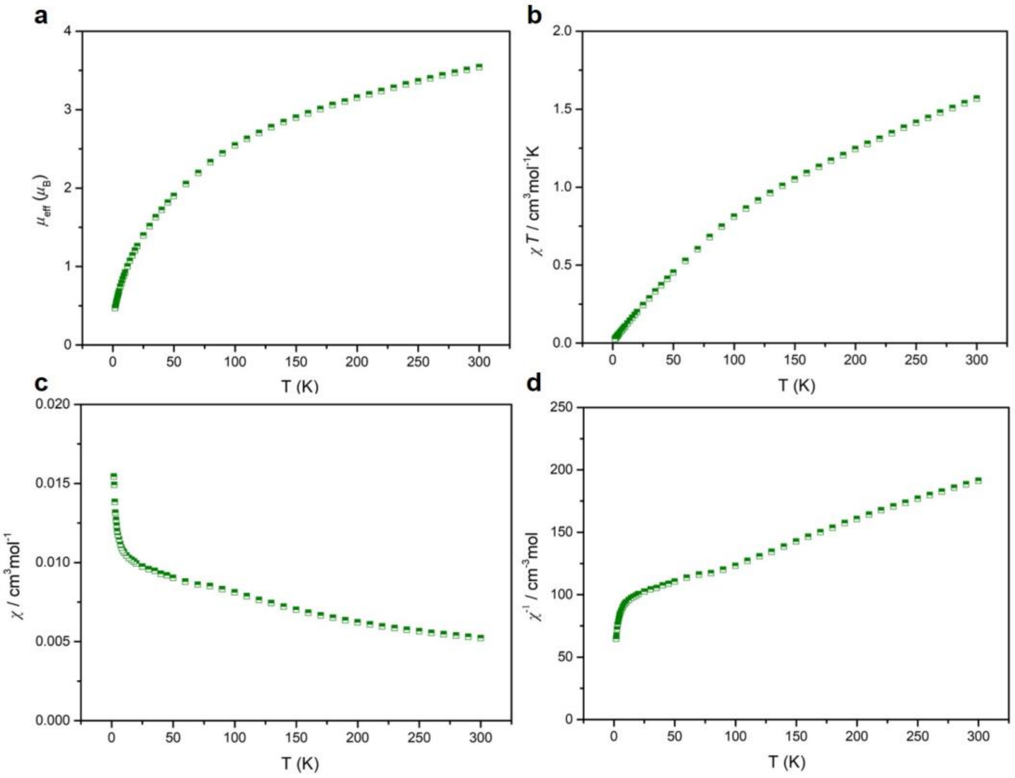

Supplementary Figure 24. Variable-temperature data of **3b**. (a) μ_{eff} vs T, (b) χT vs T, (c) χ vs T, and (d) 1/χ vs T.

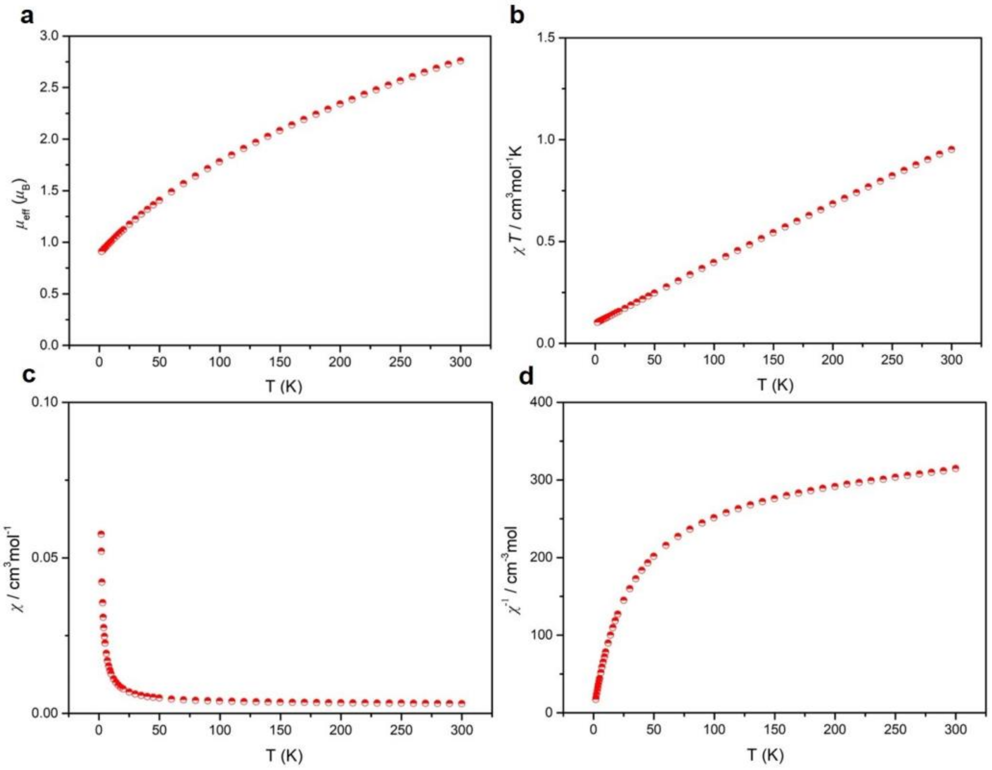

Supplementary Figure 25. Variable-temperature data of **4a**. (a) μ_{eff} vs T, (b) χT vs T, (c) χ vs T, and (d) $1/\chi$ vs T.

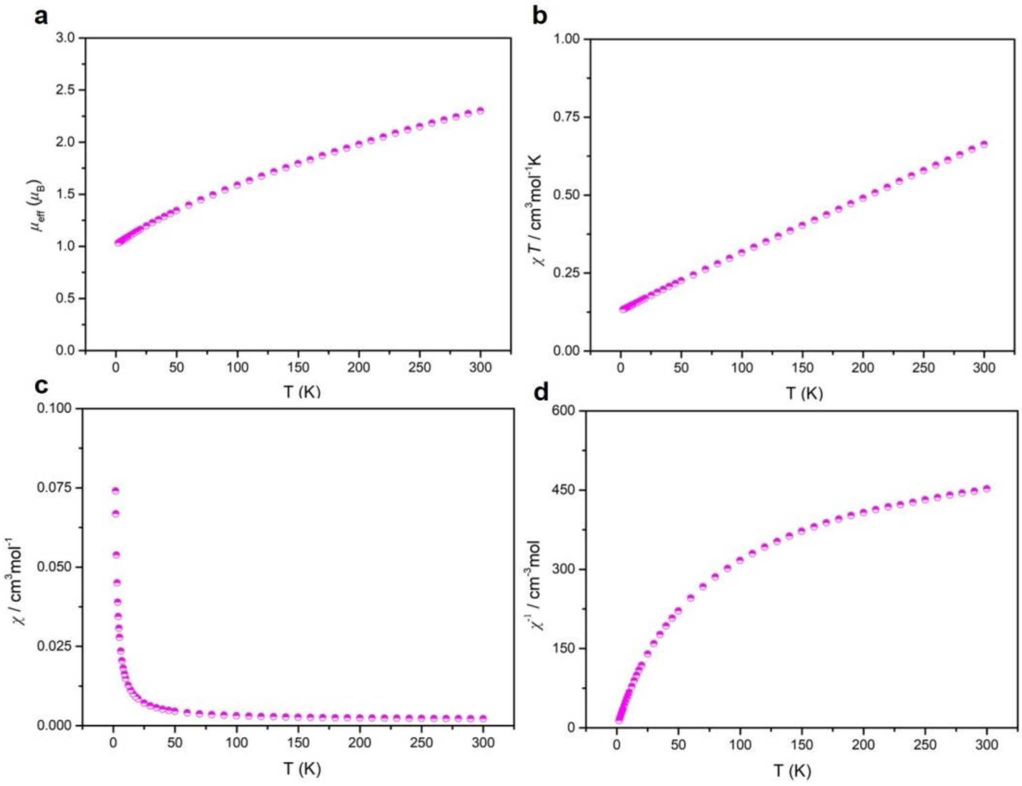

Supplementary Figure 26. Variable-temperature data of **4b**. (a) μ_{eff} vs T, (b) χT vs T, (c) χ vs T, and (d) $1/\chi$ vs T.

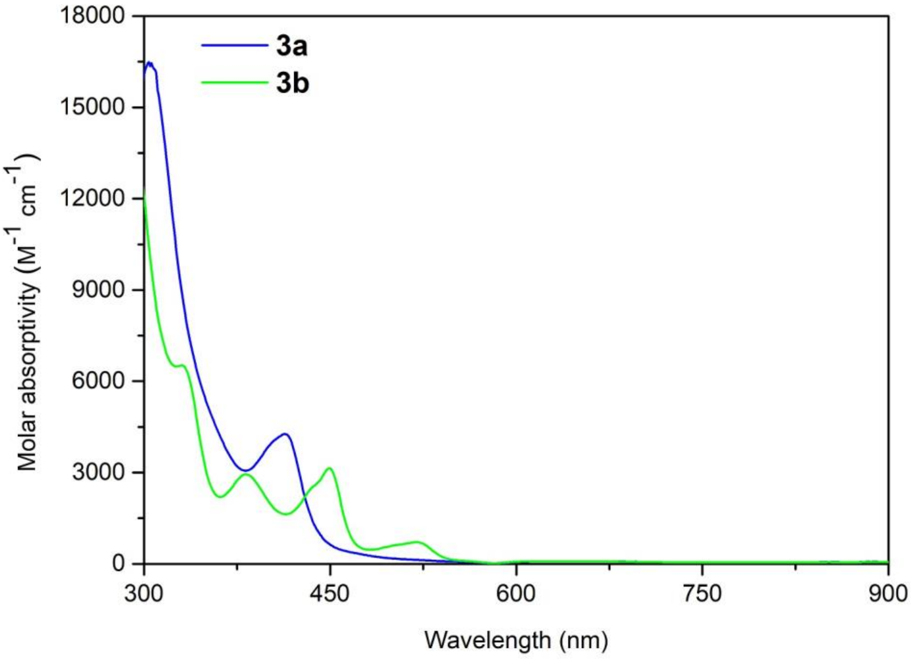

Supplementary Figure 27. UV−visible absorption spectra of complexes **3a** and **3b** measured in THF at RT.

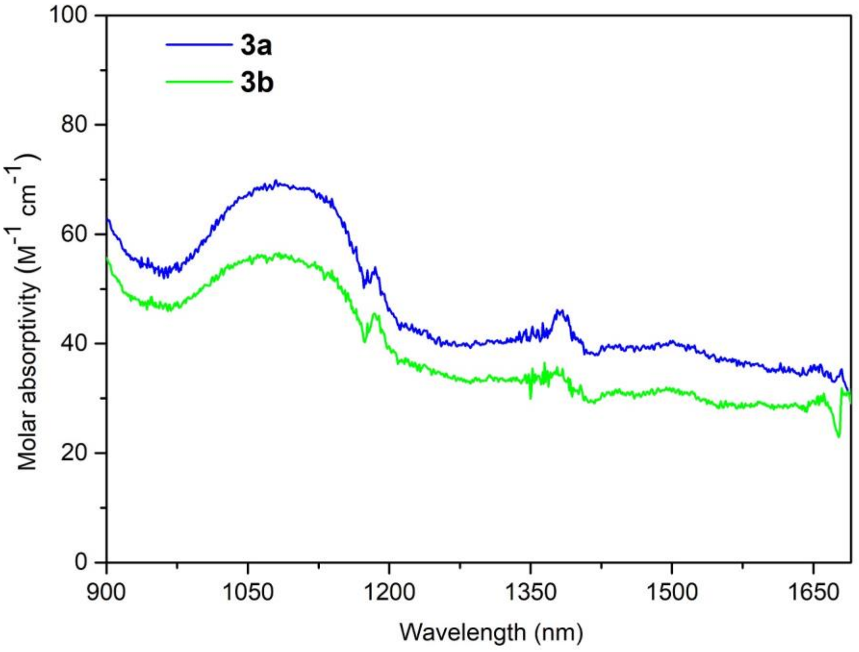

Supplementary Figure 28. Near-infrared absorption spectra of complexes **3a** and **3b** measured in THF at RT.

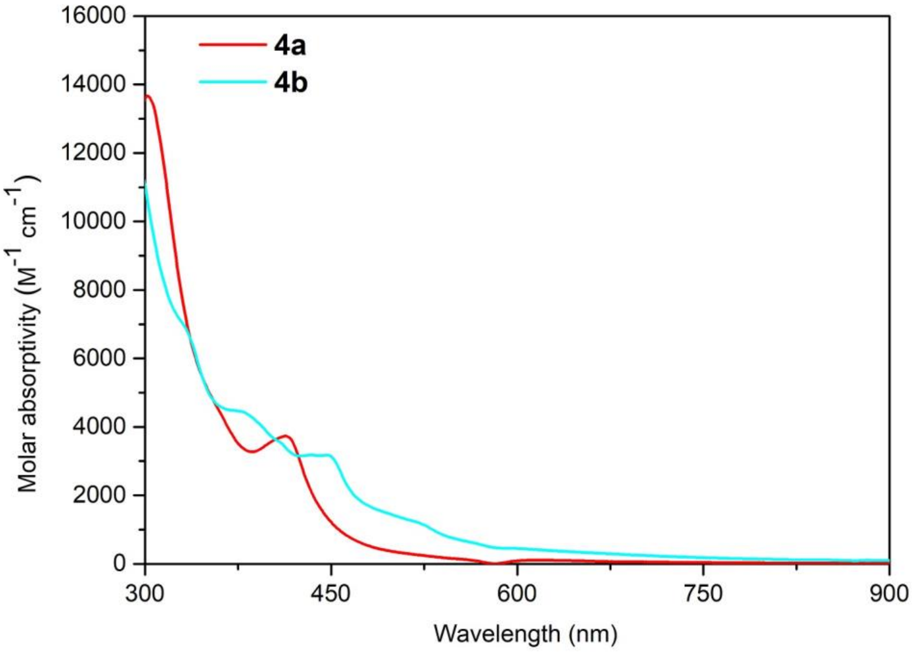

Supplementary Figure 29. UV−visible absorption spectra of complexes **4a** and **4b** measured in THF at RT.

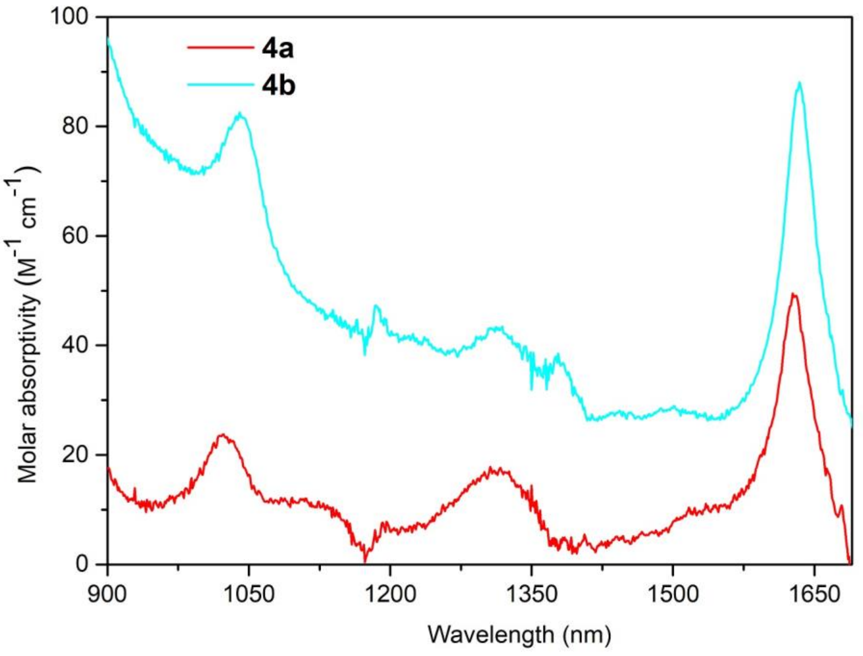

Supplementary Figure 30. Near-infrared absorption spectra of complexes **4a** and **4b** measured in THF at RT.

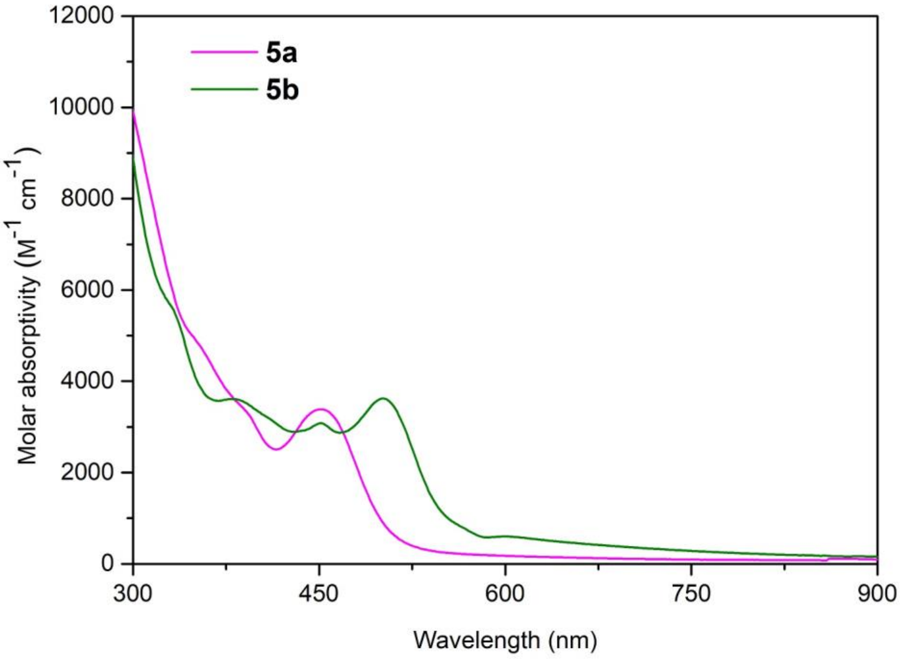

Supplementary Figure 31. UV−visible absorption spectra of complexes **5a** and **5b** measured in THF at RT.

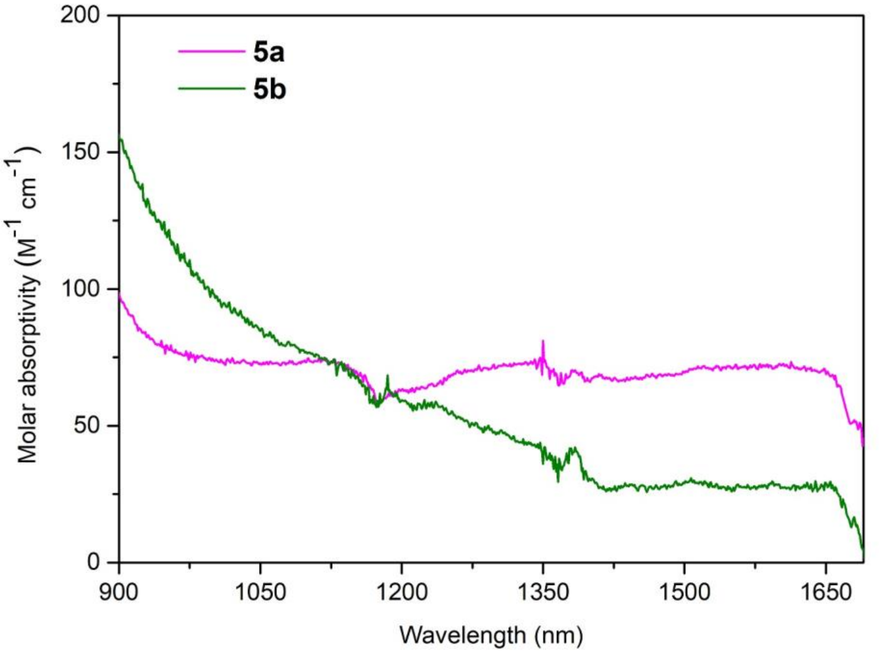

Supplementary Figure 32. Near-infrared absorption spectra of complexes **5a** and **5b** measured in THF at RT.

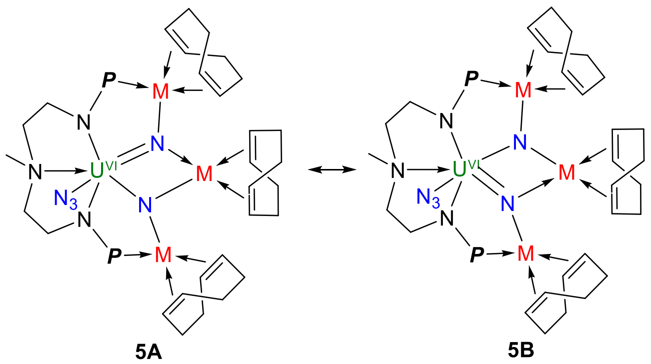

Supplementary Figure 33. The two major resonance structures of complexes **5**.

Supplementary Table 1. Crystal data and structural refinements for **2-5**.

	**2**		**3**	
	**2a**·toluene	**2b**·toluene	**3a**	**3b**
empirical formula	$C_{40}H_{71}Cl_4N_3P_2Rh_2U$	$C_{40}H_{71}Cl_4Ir_2N_3P_2U$	$C_{33}H_{63}N_{15}P_2Rh_2U$	$C_{33}H_{63}Ir_2N_{15}P_2U$
formula weight	1241.58	1420.16	1175.77	1354.35
temperature, K	193(2)	193(2)	173(2)	193(2)
wavelength, Å	0.71073	0.71073	0.71073	0.71073
crystal system	Monoclinic	Monoclinic	Monoclinic	Monoclinic
space group	P21/n	P21/n	P21/n	P21/n
a, Å	13.6450(3)	13.6338(10)	17.2026(6)	17.2027(6)
b, Å	23.4982(6)	23.4552(18)	14.8535(5)	14.9033(5)
c, Å	14.2779(4)	14.2487(10)	17.5846(5)	17.5252(5)
α, °	90	90	90	90
β, °	93.5780(10)	93.489(3)	107.3340(10)	107.3940(10)
γ, °	90	90	90	90
V, Å^3	4569.0(2)	4548.1(6)	4289.1(2)	4287.6(2)
Z	4	4	4	4
d_{calcd}, g cm^{-3}	1.805	2.074	1.821	2.098
μ, mm^{-1}	4.585	9.727	4.645	10.079
F(000)	2448.0	2704.0	2312.0	2568.0
crystal size, mm	0.1 × 0.1 × 0.1	0.1 × 0.1 × 0.1	0.13 × 0.12 × 0.1	0.13 × 0.12 × 0.1
θ_{max},°	27.502	27.402	25.343	25.349
reflns collected	42158	41852	52012	32209
indep reflns	10479 [R_{int} = 0.0388, R_{sigma} = 0.0333]	10427 [R_{int} = 0.0550, R_{sigma} = 0.0508]	7834 [R_{int} = 0.0548, R_{sigma} = 0.0330]	7846 [R_{int} = 0.0813, R_{sigma} = 0.0675]

data/restraints/params	10479/0/479	10427/0/479	7834/96/487	7846/48/487
goodness-of-fit on F^2	1.044	1.037	1.039	1.043
final R ($I > 2\sigma(I)$)	R_1 = 0.0228, wR_2 = 0.0509	R_1 = 0.0315, wR_2 = 0.0537	R_1 = 0.0310, wR_2 = 0.0769	R_1 = 0.0404, wR_2 = 0.0947
R indices (all data)	R_1 = 0.0272, wR_2 = 0.0523	R_1 = 0.0473, wR_2 = 0.0583	R_1 = 0.0360, wR_2 = 0.0794	R_1 = 0.0513, wR_2 = 0.1014
Residual electron density (e. Å^{-3}) max/min	1.40/-0.90	1.25/-1.44	1.18/-1.09	1.99/-1.64
CCDC	2104709	2104710	2104711	2104712
	**4**		**5**	
	**4a**·1.5 toluene	**4b**·1.5 toluene	**5a**	**5b**
empirical formula	$C_{51.5}H_{87}N_{13}P_2Rh_3U$	$C_{51.5}H_{87}N_{13}P_2Ir_3U$	$C_{41}H_{75}N_8P_2Rh_3U$	$C_{41}H_{75}Ir_3N_8P_2U$
formula weight	1497.04	1764.91	1288.79	1556.66
temperature, K	193(2)	193(2)	193(2)	193(2)
wavelength, Å	0.71073	0.71073	1.34139	1.34139
crystal system	Triclinic	Triclinic	Orthorhombic	Orthorhombic
space group	P-1	P-1	Pnma	Pnma
a, Å	10.4530(4)	10.4461(3)	20.762(9)	20.757(7)
b, Å	15.7083(6)	15.7690(5)	19.974(9)	20.007(6)
c, Å	18.5359(8)	18.4599(7)	10.945(5)	10.946(4)
α, °	84.196(2)	84.4400(10)	90	90
β, °	74.207(2)	73.9370(10)	90	90
γ, °	78.8360(10)	78.7250(10)	90	90
V, Å^3	2869.5(2)	2862.73(16)	4539(4)	4546(3)

Z	2	2	4	4
d_{calcd}, g cm^{-3}	1.733	2.047	1.886	2.275
μ, mm^{-1}	3.763	9.873	13.980	19.108
F(000)	1488.0	1680.0	2536.0	2920.0
crystal size, mm	0.1 × 0.1 × 0.1	0.1 × 0.1 × 0.1	0.1 × 0.1 × 0.1	0.1 × 0.1 × 0.1
θ_{max},°	27.508	27.520	54.185	53.878
reflns collected	26679	26915	41885	47269
indep reflns	12981 [R_{int} = 0.0382, R_{sigma} = 0.0542]	13033 [R_{int} = 0.0306, R_{sigma} = 0.0454]	4309 [R_{int} = 0.0867, R_{sigma} = 0.0389]	4265 [R_{int} = 0.0966, R_{sigma} = 0.0429]
data/restraints/params	12981/168/667	13033/168/666	4309/48/263	4265/48/263
goodness-of-fit on F^2	1.140	1.051	1.071	1.041
final R ($I > 2\sigma(I)$)	R_1 = 0.0408, wR_2 = 0.1046	R_1 = 0.0270, wR_2 = 0.0601	R_1 = 0.0323, wR_2 = 0.0788	R_1 = 0.0336, wR_2 = 0.0839
R indices (all data)	R_1 = 0.0449, wR_2 = 0.1070	R_1 = 0.0329, wR_2 = 0.0625	R_1 = 0.0365, wR_2 = 0.0810	R_1 = 0.0385, wR_2 = 0.0865
Residual electron density (e. Å^{-3}) max/min	2.77/-0.97	1.37/-1.89	1.52/-1.46	1.56/-1.42
CCDC	2104713	2104714	2104715	2104716

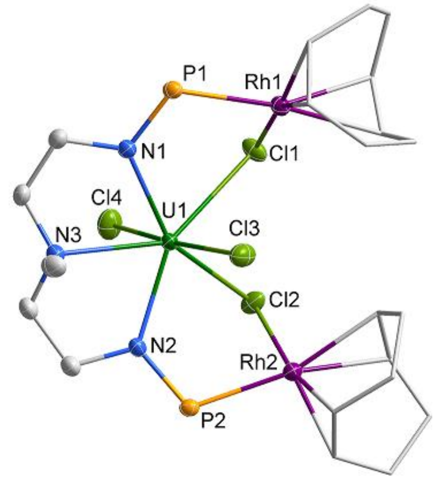

Supplementary Figure 34 X-ray molecular structure of complex **2a** drawn with 50% probability. Hydrogen atoms, solvents and isopropyl moieties in P^iPr_2 are omitted for clarity.

Supplementary Table 2. Selected bond distances (Å) and angles (˚) for **2a**.

U1-N1	2.349(2)	U1-N2	2.338(2)
U1-N3	2.567(2)	U1-Cl1	2.8325(7)
U1-Cl2	2.8150(8)	U1-Cl3	2.6452(8)
U1-Cl4	2.6483(8)	Rh1-P1	2.3301(8)
Rh1-Cl1	2.3779(8)	Rh2-P2	2.3293(8)
Rh2-Cl2	2.3652(8)		
Cl2-U1-Cl1	71.75(2)	N1-U1-Cl1	79.31(6)
Cl3-U1-Cl1	89.45(2)	N1-U1-Cl2	150.58(6)
Cl3-U1-Cl2	95.28(2)	N1-U1-Cl3	89.50(6)
Cl3-U1-Cl4	172.59(3)	N1-U1-Cl4	92.73(6)
Cl4-U1-Cl1	84.03(2)	N1-U1-N3	66.13(8)
Cl4-U1-Cl2	79.36(3)	N2-U1-Cl1	150.18(6)
N2-U1-Cl3	89.78(6)	N3-U1-Cl1	141.56(6)
N2-U1-Cl4	94.13(6)	N3-U1-Cl2	138.65(6)
N2-U1-N1	130.49(8)	N3-U1-Cl3	105.90(6)

N2-U1-N3	66.60(8)	N3-U1-Cl4	81.45(6)
Rh1-Cl1-U1	95.39(2)	P1-Rh1-Cl1	88.76(3)
Rh2-Cl2-U1	93.37(2)		

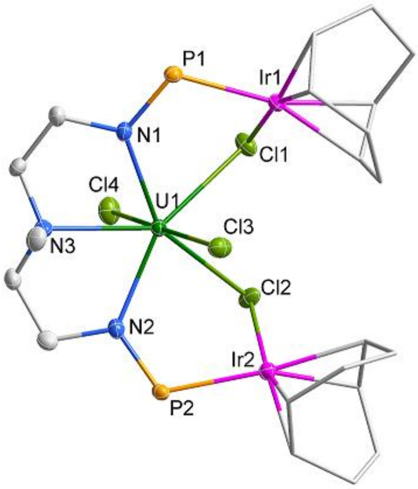

Supplementary Figure 35. X-ray molecular structure of complex **2b** drawn with 50% probability. Hydrogen atoms, solvents and isopropyl moieties in $P^{i}Pr_2$ are omitted for clarity.

Supplementary Table 3. Selected bond distances (Å) and angles (˚) for **2b**.

U1-N1	2.330(4)	U1-N2	2.345(4)
U1-N3	2.556(4)	U1-Cl1	2.8492(14)
U1-Cl3	2.6358(13)	U1-Cl2	2.8612(14)
U1-Cl4	2.6439(14)	Ir2-Cl2	2.3634(13)
Ir1-Cl1	2.3557(14)	Ir2-P2	2.3319(14)
Ir1-P1	2.3335(14)		
Cl1-U1-Cl2	70.29(4)	N3-U1-Cl1	138.47(11)
Cl3-U1-Cl1	97.66(4)	N3-U1-Cl2	141.99(11)
Cl3-U1-Cl2	90.88(4)	N3-U1-Cl3	104.81(11)
Cl3-U1-Cl4	173.47(5)	N3-U1-Cl4	81.52(11)

Cl4-U1-Cl1	78.15(4)	N2-U1-Cl1	149.03(11)
Cl4-U1-Cl2	83.00(4)	N2-U1-Cl2	79.48(11)
N1-U1-Cl1	78.63(11)	N2-U1-Cl3	89.22(11)
N1-U1-Cl2	148.78(11)	N2-U1-Cl4	91.91(11)
N1-U1-Cl3	90.25(11)	N2-U1-N3	66.62(15)
N1-U1-Cl4	93.79(11)	Ir1-Cl1-U1	92.95(4)
N1-U1-N3	66.97(15)	Ir2-Cl2-U1	94.66(4)
N1-U1-N2	131.74(15)		

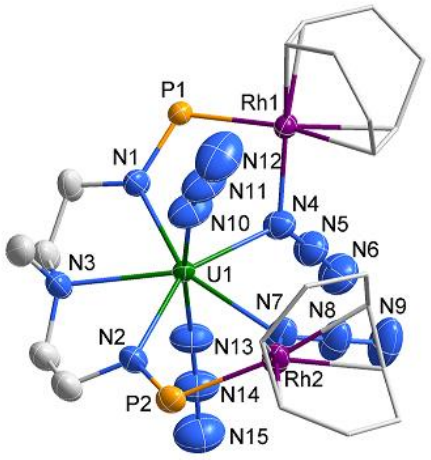

Supplementary Figure 36. X-ray molecular structure of complex **3a** drawn with 50% probability. Hydrogen atoms and isopropyl moieties in P^iPr_2 are omitted for clarity.

Supplementary Table 4. Selected bond distances (Å) and angles (˚) for **3a**.

U1-N1	2.355(4)	U1-N2	2.360(4)
U1-N3	2.588(4)	U1-N4	2.453(5)
U1-N7	2.515(5)	U1-N10	2.307(5)
U1-N13	2.304(5)	N4-N5	1.197(7)
N5-N6	1.159(7)	N7-N8	1.228(7)
N8-N9	1.144(7)	N10-N11	1.140(7)

N11-N12	1.147(8)	N13-N14	1.179(7)
N14-N15	1.138(8)	Rh2-P2	2.3304(15)
Rh1-P1	2.3142(13)	Rh2-N7	2.103(5)
Rh1-N4	2.091(5)		
N10-U1-Rh1	70.39(17)	N10-U1-N7	87.0(2)
N10-U1-N4	98.8(2)	N10-U1-N2	90.7(2)
N10-U1-N3	106.2(2)	N10-U1-N1	92.64(19)
N7-U1-Rh1	91.59(13)	N7-U1-N3	140.80(16)
N4-U1-Rh1	33.59(11)	N4-U1-N7	75.20(16)
N4-U1-N3	135.70(16)	N13-U1-Rh1	107.90(14)
N13-U1-N10	172.0(2)	N13-U1-N7	85.2(2)
N13-U1-N4	77.39(18)	N13-U1-N2	89.46(18)
N13-U1-N3	81.17(19)	N13-U1-N1	93.39(18)
N2-U1-Rh1	158.61(11)	N2-U1-N7	77.15(16)
N2-U1-N4	150.19(16)	N2-U1-N3	66.20(15)
N3-U1-Rh1	127.56(10)	N1-U1-Rh1	62.43(10)
N1-U1-N7	152.25(16)	N1-U1-N4	77.45(16)
N1-U1-N2	130.59(16)	N1-U1-N3	65.58(14)
N7-Rh2-P2	85.19(16)	P1-Rh1-U1	67.66(3)
N4-Rh1-U1	40.45(13)	N4-Rh1-P1	86.62(14)
N2-P2-Rh2	113.64(16)	N1-P1-Rh1	110.42(16)
N11-N10-U1	168.6(6)	N10-N11-N12	178.7(10)
Rh2-N7-U1	113.8(2)	N8-N7-U1	125.2(4)
N8-N7-Rh2	118.1(5)	N9-N8-N7	178.9(7)
Rh1-N4-U1	105.97(19)	N5-N4-U1	128.7(4)
N5-N4-Rh1	124.4(4)	N6-N5-N4	175.8(7)
N14-N13-U1	160.5(5)	N15-N14-N13	177.2(9)
P2-N2-U1	122.6(2)	P1-N1-U1	119.3(2)

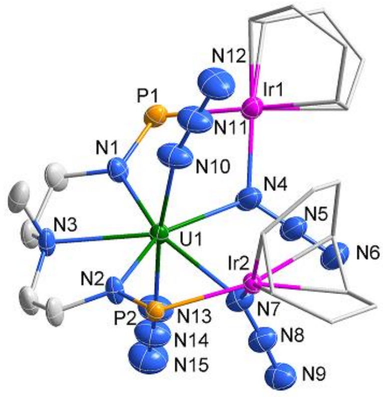

Supplementary Figure 37. X-ray molecular structure of complex **3b** drawn with 50% probability. Hydrogen atoms and isopropyl moieties in $P^{i}Pr_2$ are omitted for clarity.

Supplementary Table 5. Selected bond distances (Å) and angles (˚) for **3b**.

U1-N1	2.369(7)	U1-N2	2.340(6)
U1-N3	2.569(6)	U1-N4	2.533(8)
U1-N7	2.475(7)	U1-N10	2.295(8)
U1-N13	2.311(8)	N4-N5	1.244(11)
N5-N6	1.137(11)	N7-N8	1.229(10)
N8-N9	1.117(10)	N10-N11	1.148(11)
N11-N12	1.157(12)	N13-N14	1.168(11)
N14-N15	1.126(12)	Ir1-N4	2.086(8)
Ir1-P1	2.336(2)	Ir2-N7	2.072(7)
Ir2-P2	2.321(2)		
N1-U1-Ir2	159.09(17)	N1-U1-N3	66.4(2)
N1-U1-N4	77.4(2)	N1-U1-N7	149.4(2)
N2-U1-Ir2	62.85(16)	N2-U1-N1	130.9(2)
N2-U1-N3	65.4(2)	N2-U1-N4	151.7(2)

N2-U1-N7	77.6(2)	N3-U1-Ir2	127.86(15)
N4-U1-Ir2	90.4(2)	N4-U1-N3	141.7(3)
N7-U1-Ir2	33.32(17)	N7-U1-N3	135.8(2)
N7-U1-N4	74.6(2)	N10-U1-Ir2	71.9(3)
N10-U1-N1	90.2(3)	N10-U1-N2	93.2(3)
N10-U1-N3	105.2(3)	N10-U1-N4	86.6(3)
N10-U1-N7	100.1(3)	N10-U1-N13	171.7(3)
N13-U1-Ir2	107.2(2)	N13-U1-N1	89.0(3)
N13-U1-N2	93.6(3)	N13-U1-N3	82.0(3)
N13-U1-N4	85.2(3)	N13-U1-N7	76.7(3)
N4-Ir1-P1	85.7(2)	P2-Ir2-U1	67.50(5)
N7-Ir2-U1	41.0(2)	N7-Ir2-P2	86.7(2)
N1-P1-Ir1	114.3(3)	N2-P2-Ir2	110.3(2)
P1-N1-U1	122.2(4)	P2-N2-U1	119.2(3)
Ir1-N4-U1	113.8(3)	N5-N4-U1	124.6(6)
N5-N4-Ir1	119.4(6)	N6-N5-N4	178.2(12)
Ir2-N7-U1	105.7(3)	N8-N7-U1	128.3(6)
N8-N7-Ir2	125.1(6)	N9-N8-N7	176.1(11)
N11-N10-U1	165.6(9)	N10-N11-N12	178.7(13)
N14-N13-U1	161.4(8)	N15-N14-N13	178.2(14)

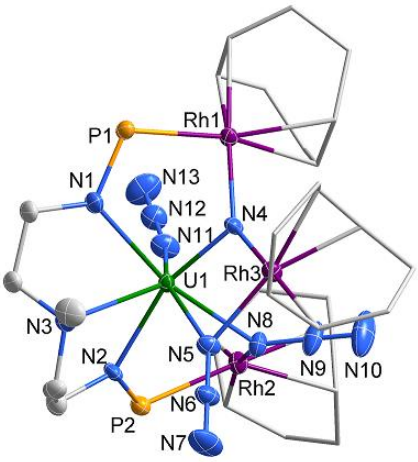

Supplementary Figure 38. X-ray molecular structure of complex **4a** drawn with 50% probability. Hydrogen atoms, solvents and isopropyl moieties in $P^{i}Pr_2$ are omitted for clarity.

Supplementary Table 6. Selected bond distances (Å) and angles (˚) for **4a**.

U1-N1	2.334(5)	U1-N2	2.328(5)
U1-N3	2.740(5)	U1-N4	1.963(5)
U1-N5	2.431(5)	U1-N8	2.507(5)
U1-N11	2.373(5)	N5-N6	1.201(7)
N6-N7	1.147(8)	N8-N9	1.226(8)
N9-N10	1.133(9)	N11-N12	1.170(8)
N12-N13	1.150(8)	Rh1-N4	2.087(5)
Rh2-N8	2.095(5)	Rh3-N4	2.081(5)
Rh3-N5	2.099(6)	Rh1-P1	2.2976(16)
Rh2-P2	2.3515(17)		
Rh3-U1-Rh1	73.860(12)	N1-U1-N8	164.41(18)
N4-U1-Rh1	36.12(14)	N1-U1-N5	115.36(18)
N4-U1-Rh3	38.14(14)	N1-U1-N11	90.0(2)
N4-U1-N8	84.48(19)	N1-U1-N3	64.18(17)
N4-U1-N5	78.52(19)	N11-U1-Rh1	68.70(15)
N4-U1-N2	162.40(19)	N11-U1-Rh3	132.41(15)
N4-U1-N1	86.7(2)	N11-U1-N8	78.62(19)
N4-U1-N11	98.3(2)	N11-U1-N5	154.0(2)
N4-U1-N3	121.92(19)	N11-U1-N3	128.37(19)
N8-U1-Rh1	98.26(13)	N3-U1-Rh1	127.68(11)
N8-U1-Rh3	78.81(12)	N3-U1-Rh3	97.64(12)
N8-U1-N3	131.38(17)	N4-Rh1-U1	33.67(14)
N5-U1-Rh1	114.26(13)	N4-Rh3-U1	35.62(14)

N5-U1-Rh3	40.44(13)	N4-Rh3-N5	84.2(2)
N5-U1-N8	75.37(17)	N5-Rh3-U1	48.69(14)
N5-U1-N3	71.96(17)	U1-N4-Rh1	110.2(2)
N2-U1-Rh1	153.47(13)	U1-N4-Rh3	106.2(2)
N2-U1-Rh3	130.31(13)	Rh3-N4-Rh1	142.0(3)
N2-U1-N8	79.22(18)	Rh2-N8-U1	108.8(2)
N2-U1-N5	90.86(18)	N9-N8-U1	122.4(4)
N2-U1-N1	110.67(19)	N9-N8-Rh2	121.3(4)
N2-U1-N11	85.0(2)	Rh3-N5-U1	90.87(19)
N2-U1-N3	66.44(17)	N6-N5-U1	146.6(5)
N1-U1-Rh1	67.39(13)	N6-N5-Rh3	122.5(5)
N1-U1-Rh3	101.84(14)	N10-N9-N8	177.8(9)
N7-N6-N5	179.1(9)	N13-N12-N11	179.6(9)
N12-N11-U1	163.4(5)		

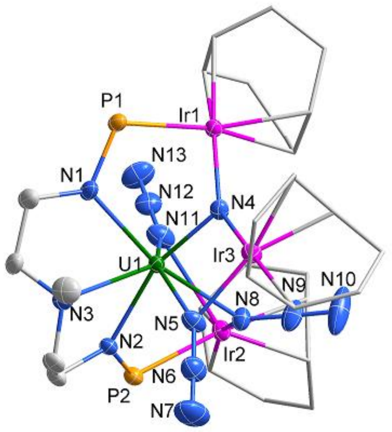

Supplementary Figure 39. X-ray molecular structure of complex **4b** drawn with 50% probability. Hydrogen atoms, solvents and isopropyl moieties in P^iPr_2 are omitted for clarity.

Supplementary Table 7. Selected bond distances (Å) and angles (˚) for **4b**.

U1-N1	2.326(4)	Ir2-P2	2.3595(11)
U1-N2	2.313(4)	Ir3-N4	2.055(4)
U1-N3	2.726(3)	Ir3-N5	2.089(4)
U1-N4	2.011(4)	N5-N6	1.231(6)
U1-N5	2.384(4)	N6-N7	1.142(7)
U1-N8	2.498(4)	N8-N9	1.223(5)
U1-N11	2.386(4)	N9-N10	1.142(6)
Ir1-P1	2.3012(11)	N11-N12	1.205(6)
Ir1-N4	2.064(4)	N12-N13	1.142(6)
Ir2-N8	2.094(4)		
N1-U1-Ir1	67.08(9)	N5-U1-Ir1	114.44(10)
N1-U1-Ir3	102.34(10)	N5-U1-Ir3	40.88(10)
N1-U1-N8	162.91(13)	N5-U1-N8	76.07(14)
N1-U1-N11	93.70(16)	N5-U1-N11	149.57(16)
N1-U1-N5	116.26(14)	N5-U1-N3	71.96(13)
N1-U1-N3	64.61(13)	N8-U1-Ir1	97.49(9)
N2-U1-Ir1	153.72(9)	N8-U1-Ir3	79.03(9)
N2-U1-Ir3	130.18(9)	N8-U1-N3	132.38(13)
N2-U1-N8	79.40(13)	N11-U1-Ir1	70.92(11)
N2-U1-N11	83.29(14)	N11-U1-Ir3	131.11(10)
N2-U1-N5	90.34(14)	N11-U1-N8	73.51(15)
N2-U1-N3	66.63(12)	N11-U1-N3	130.47(13)
N2-U1-N1	110.92(13)	N7-N6-N5	178.2(6)
N3-U1-Ir1	127.56(9)	N10-N9-N8	177.8(6)
N3-U1-Ir3	97.84(9)	N13-N12-N11	176.8(6)
N4-U1-Ir1	35.31(11)	N4-Ir1-U1	34.28(10)

N4-U1-Ir3	38.77(10)	N4-Ir3-U1	37.80(10)
N4-U1-N1	86.75(15)	N4-Ir3-N5	86.04(15)
N4-U1-N2	162.22(15)	N5-Ir3-U1	48.32(11)
N4-U1-N3	122.61(13)	U1-N4-Ir1	110.41(16)
N4-U1-N5	79.58(14)	U1-N4-Ir3	103.42(17)
N4-U1-N8	83.95(14)	Ir3-N4-Ir1	144.1(2)
N4-U1-N11	97.97(14)	Ir2-N8-U1	106.32(15)
Ir3-U1-Ir1	73.603(6)	N9-N8-U1	124.3(3)
N9-N8-Ir2	122.0(3)	N12-N11-U1	152.3(4)
Ir3-N5-U1	90.80(15)	N6-N5-U1	147.0(3)
N6-N5-Ir3	122.0(3)		

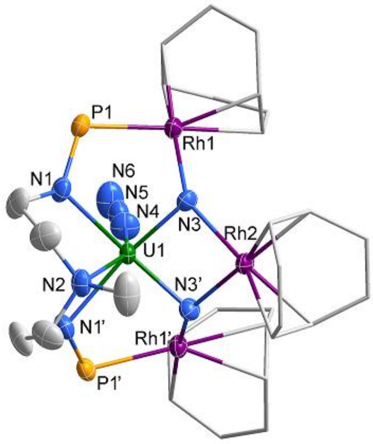

Supplementary Figure 40. X-ray molecular structure of complex **5a** drawn with 50% probability. Hydrogen atoms and isopropyl moieties in P^iPr_2 are omitted for clarity.

Supplementary Table 8. Selected bond distances (Å) and angles (˚) for **5a**.

U1-N1	2.298(5)	U1-N1’	2.298(5)
U1-N2	2.703(6)	U1-N3’	1.975(5)
U1-N3	1.975(5)	U1-N4	2.334(7)
Rh1-N3	2.082(4)	Rh1-P1	2.3257(16)

U1-P1	3.2239(18)	U1-P1’	3.2238(18)
Rh2-N3	2.067(4)	Rh2-N3’	2.067(4)
N4-N5	1.172(10)	N5-N6	1.181(10)
Rh1’-U1-Rh1	114.11(3)	Rh2-U1-Rh1’	79.492(15)
Rh2-U1-Rh1	79.492(15)	N1-U1-Rh1’	155.14(12)
N1’-U1-Rh1	155.14(12)	N1’-U1-Rh1’	71.05(12)
N1-U1-Rh1	71.06(12)	N1’-U1-Rh2	124.95(12)
N1-U1-Rh2	124.95(12)	N1-U1-N1’	94.5(2)
N1’-U1-N2	67.15(14)	N1-U1-N2	67.15(14)
N1-U1-N4	96.53(17)	N1’-U1-N4	96.53(17)
N2-U1-Rh1’	121.30(3)	N2-U1-Rh1	121.30(3)
N2-U1-Rh2	92.39(14)	N3’-U1-Rh1	111.19(13)
N3-U1-Rh1	39.18(12)	N3-U1-Rh1’	111.19(13)
N3’-U1-Rh1’	39.18(12)	N3-U1-Rh2	43.87(13)
N3’-U1-Rh2	43.87(13)	N3’-U1-N1	164.52(17)
N3’-U1-N1’	88.26(18)	N3-U1-N1’	164.52(17)
N3-U1-N1	88.26(18)	N3’-U1-N2	100.20(16)
N3-U1-N2	100.20(16)	N3’-U1-N3	85.2(3)
N3-U1-N4	98.30(19)	N3’-U1-N4	98.30(19)
N4-U1-Rh1	66.34(8)	N4-U1-Rh1’	66.34(8)
N4-U1-Rh2	112.85(19)	N4-U1-N2	154.8(2)
N3-Rh1-U1	36.82(12)	N3’-Rh2-U1	41.46(13)
N3-Rh2-U1	41.46(13)	N3’-Rh2-N3	80.6(3)
U1-N3-Rh1	104.00(19)	U1-N3-Rh2	94.68(19)
Rh2-N3-Rh1	144.3(2)	N5-N4-U1	173.4(7)
N4-N5-N6	178.5(9)		

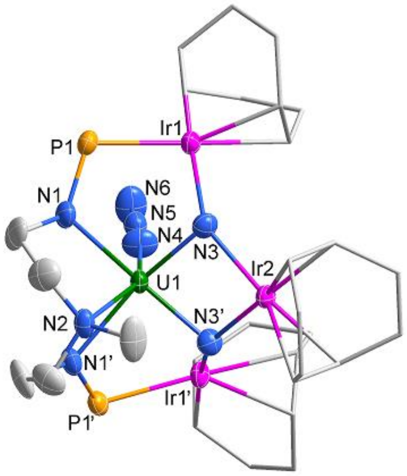

Supplementary Figure 41. X-ray molecular structure of complex **5b** drawn with 50% probability. Hydrogen atoms and isopropyl moieties in $P^{i}Pr_2$ are omitted for clarity.

Supplementary Table 9. Selected bond distances (Å) and angles (˚) for **5b**.

U1-N1	2.292(6)	U1-N1’	2.292(6)
U1-N2	2.720(7)	U1-N3’	2.005(6)
U1-N3	2.005(6)	U1-N4	2.329(8)
Ir1-P1	2.3278(18)	Ir1-N3	2.059(6)
Ir2-N3	2.047(6)	Ir2-N3’	2.047(6)
N4-N5	1.173(11)	N5-N6	1.170(12)
U1-P1	3.2156(19)	U1-P1’	3.2156(19)
Ir1'-U1-Ir1	113.66(3)	Ir2-U1-Ir1	79.160(13)
Ir2-U1-Ir1’	79.161(13)	N1-U1-Ir1’	155.94(16)
N1-U1-Ir1	71.36(14)	N1’-U1-Ir1’	71.36(14)
N1’-U1-Ir1	155.94(16)	N1-U1-Ir2	124.49(16)
N1’-U1-Ir2	124.49(15)	N1-U1-N1’	94.8(3)
N1-U1-N2	66.96(17)	N1’-U1-N2	66.96(17)
N1-U1-N4	96.6(2)	N1’-U1-N4	96.6(2)
N2-U1-Ir1	121.36(4)	N2-U1-Ir1’	121.36(4)
N2-U1-Ir2	92.15(17)	N3’-U1-Ir1	109.97(17)

N3-U1-Ir1’	109.97(17)	N3-U1-Ir1	39.01(16)
N3’-U1-Ir1’	39.01(16)	N3-U1-Ir2	43.36(17)
N3’-U1-Ir2	43.35(17)	N3-U1-N1	88.7(2)
N3-U1-N1’	164.2(2)	N3’-U1-N1	164.2(2)
N3’-U1-N1’	88.7(2)	N3’-U1-N2	100.6(2)
N3-U1-N2	100.6(2)	N3’-U1-N3	83.9(3)
N3-U1-N4	98.3(2)	N3’-U1-N4	98.3(2)
N4-U1-Ir1	66.62(11)	N4-U1-Ir1’	66.62(11)
N4-U1-Ir2	113.4(3)	N4-U1-N2	154.4(3)
N3-Ir2-U1	42.24(17)	N3-Ir1-U1	37.79(17)
N3’-Ir2-U1	42.24(17)	N3’-Ir2-N3	81.7(3)
U1-N3-Ir1	103.2(3)	U1-N3-Ir2	94.4(3)
Ir2-N3-Ir1	146.0(3)	N5-N4-U1	174.5(9)
N6-N5-N4	179.2(11)		

Supplementary Table 10. Calculated NBO compositions of the U-N(nitride) natural orbitals in the complexes **4** and **5**.

	$WBI_{U\text{-}N}$	N%	U%	U 6d:5f	N%	U%	U 6d:5f
		σ-component			π-component		
U1-N4							
**4a**	1.96	68.06	31.94	19.61:78.23	72.48	27.52	38.87:59.04
**4b**	1.83	72.25	27.75	32.70:59.24	74.55	25.45	33.05:57.37
U1-N3							
**5a**	1.91	71.28	28.72	28.69:68.04	72.40	27.60	38.54:58.34
**5b**	1.80	71.78	28.22	37.69:58.81	74.49	25.51	39.33:49.55
U1-N3'							
**5a**	1.92	86.57	13.43	44.50:33.28	72.35	27.65	38.80:58.46
**5b**	1.80	71.87	28.13	36.89:59.30	74.74	25.26	40.64:48.11

<u>Complex Rh 3a:</u>

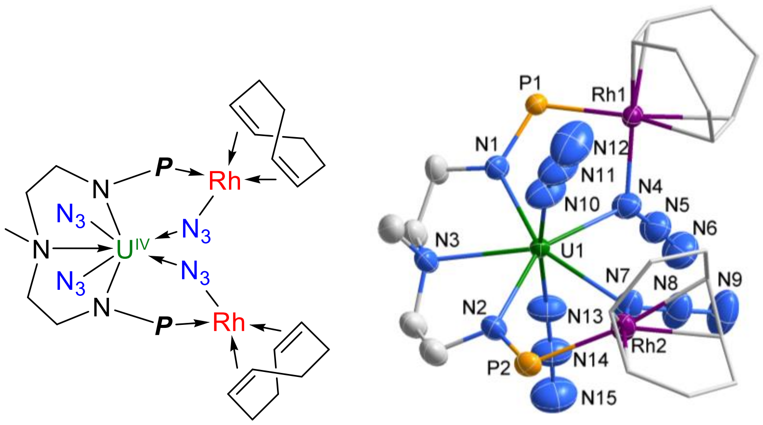

Supplementary Figure 42. Structures of complex **3a**. Unpaired spin density on U(IV) = 2.155959.

LUMO	SOMO	SOMO-1
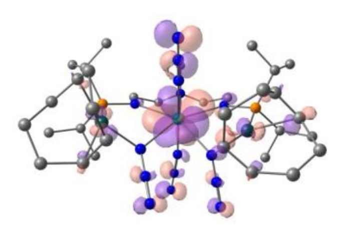	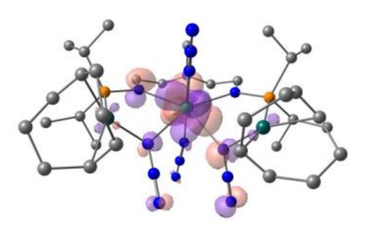	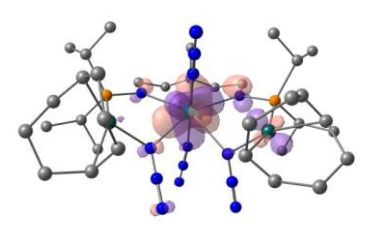
-1.488202404 eV	-4.90353032 eV	-4.929925572 eV
0.375: BD*(1) U1-N3*<br>-0.363: BD*(1) U1-N4*<br>-0.354: BD(1) U1-N7<br>0.275: BD*(1) U1-N1*<br>-0.228: BD*(3) N8-N9*		

Supplementary Figure 43. Frontier orbitals and associated energies of complex **3a**.

<u>Complex Rh 4a:</u>

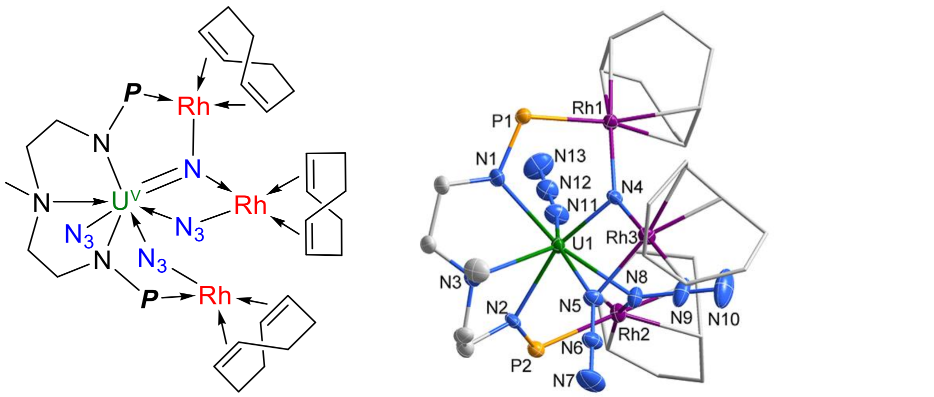

Supplementary Figure 44. Structures of complex **4a**. Unpaired spin density on U(V) = 1.235121

Supplementary Table 11. Comparison of selected bond distances (computed and experimental) for complex **4a**.

Distances	EXP	computational	Wiberg Bond Index
U1-N4	1.963(5)	1.93200	1.9569
U1-N5	2.431(5)	2.42466	0.5673
U1-N8	2.507(5)	2.52730	0.7667
U1-N11	2.373(5)	2.33398	0.7126
Rh1-N4	2.087(5)	2.12066	0.4538
Rh3-N4	2.081(5)	2.12596	0.4497
Rh3-N5	2.099(6)	2.11456	0.3955
Rh2-N8	2.095(5)	2.12485	0.4187

LUMO	SOMO
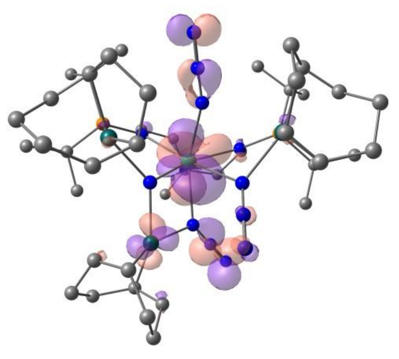	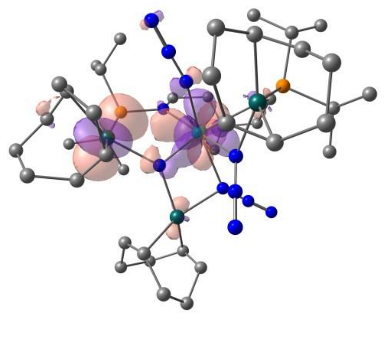
-1.476501416 eV	-4.954416012 eV
-0.421: 3C(1) U1-Rh2-N5<br>0.346: LV (3) U1(lv)<br>-0.316: BD*(3) N6- N7*<br>-0.224: LV (5) U1(lv)	

Supplementary Figure 45. Frontier orbitals and associated energies of complex **4a**.

Supplementary Table 12. Atomic orbital composition of the main bonding orbitals from NBO analysis for complex **4a**

**1. U 1 - N 4**	(31.94%) U s(0.98%) p (1.15%) d(19.61%) f(78.23%)<br>(68.06%) N s(34.73%) p (65.11%) d (0.16%)
**2. U 1 - N 4**	(27.52%) U s(0.77%) p(1.30%) d (38.87%) f (59.04%)<br>(72.48%) N s(7.40%) p(92.46%) d(0.15%)
**3. U 1 - N 5**	(21.55%)U s(0.02%) p(2.79%) d(45.55%) f(51.62%)<br>(78.45%) N s(0.13%) p(99.83%) d(0.04%)
**4. U 1 - N 8**	(10.93%) U s(11.09%)p (21.35%)d (39.57%)f (27.99%)<br>(89.07%) N s(57.71%)p (42.24%)d (0.05%)
**5. Rh 3 - N 5**	(25.98%) Rh s(4.95%)p (2.28%)d(92.77%)<br>(74.02%) N s(7.29%)p(92.65%)d (0.06%)
**6. Rh 2 - P 2**	(36.04%) Rh s(12.26%)p (14.58%)d (73.17%)<br>(63.96%) P s(35.24%)p (64.66%)d (0.10%)
**7. Rh 1 - P 1**	(39.94%) Rh s(10.68%)p (9.48%)d (79.84%)<br>(60.06%) P s(34.93%)p (64.94%)d (0.12%)

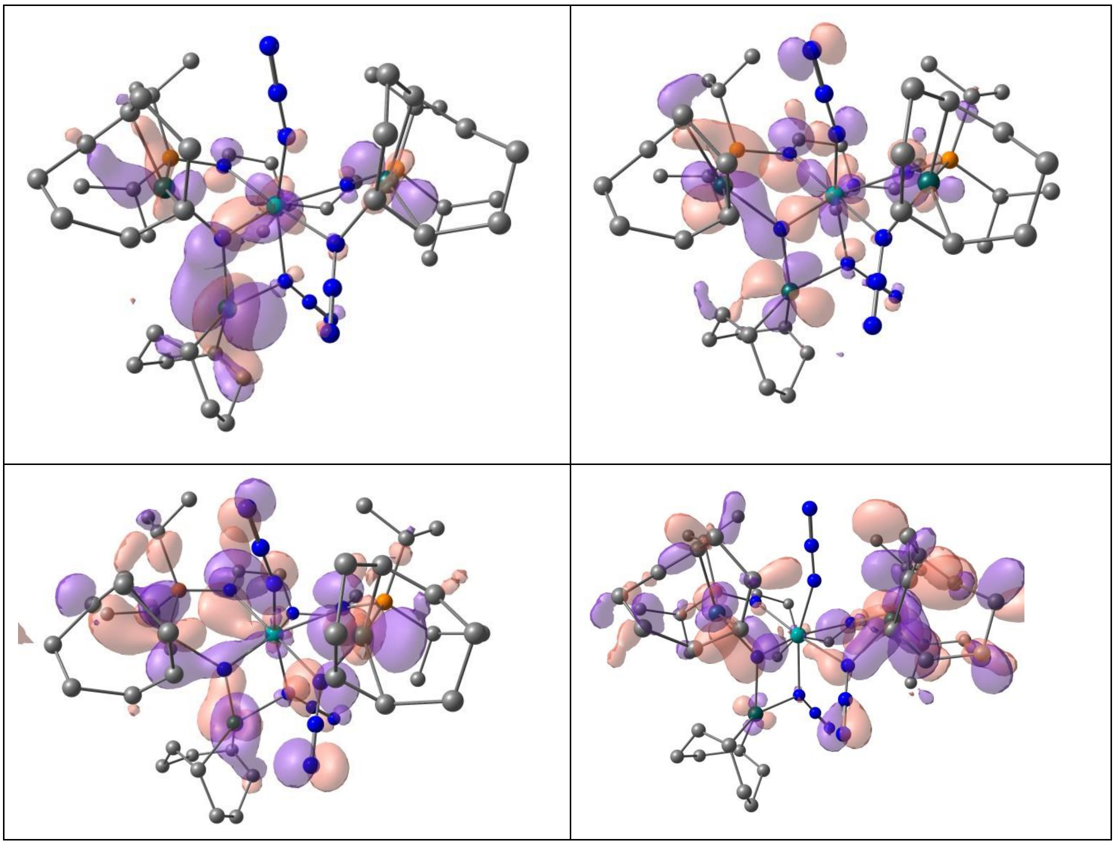

Supplementary Figure 46. Bonding U-N-Rh Molecular orbitals of complex **4a**.

Supplementary Table 13 Composition of the NLMO associated with the U1-N4 bonding NBO of complex **4a**.

111. 89.2871% U1-N4	24.150% U s(1.44%)p (0.34%)d(20.79%) f(77.40%)g(0.03%)<br>6.324% Rh1 s(74.41%)p (3.83%)d (21.76%)<br>2.196% Rh3 s(49.83%)p (6.66%)d (43.51%)<br>66.417% N4 s(53.88%)p (45.99%)d (0.13%)
112. 88.1415% U1-N4	23.373% U s(0.31%)p (0.07%)d(36.76%) f(62.83%)g(0.02%)<br>2.322% Rh1 s(1.17%)p (6.23%)d(92.60%)<br>6.178% Rh3 s(13.77%)p(11.10%)d(75.13%)<br>65.419% N4 s(4.11%)p(95.75%)d(0.14%)

Complex Rh 5a:

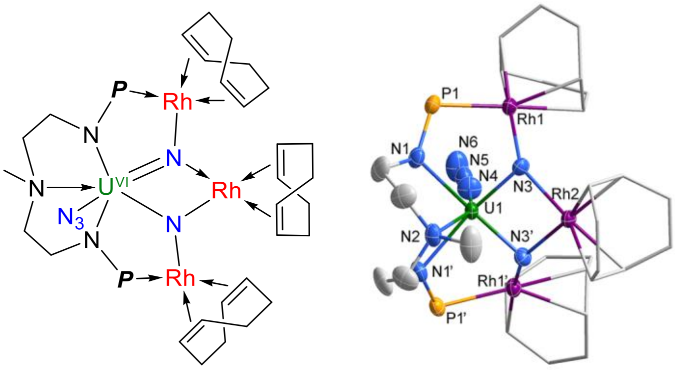

Supplementary Figure 47. Structures of complex **5a**.

Supplementary Table 14. Comparison of selected bond distances (computed and experimental) for complex **5a.**

Distances	EXP	computational	Wiberg Bond Index
U1-N3	1.975(5)	1.94775	1.9128
U1-N3’	1.975(5)	1.95004	1.9208
U1-N4	2.334(7)	2.30051	0.7622
Rh1-N3	2.082(4)	2.08718	0.5116
Rh2-N3	2.067(4)	2.09437	0.4873
Rh2-N3’	2.067(4)	2.09940	0.4898
Rh1’-N3’	2.082(4)	2.10116	0.5076

LUMO	HOMO
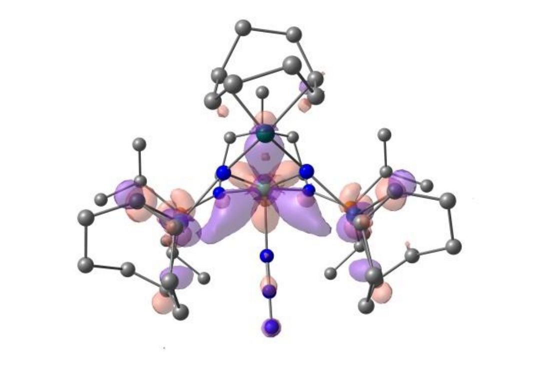	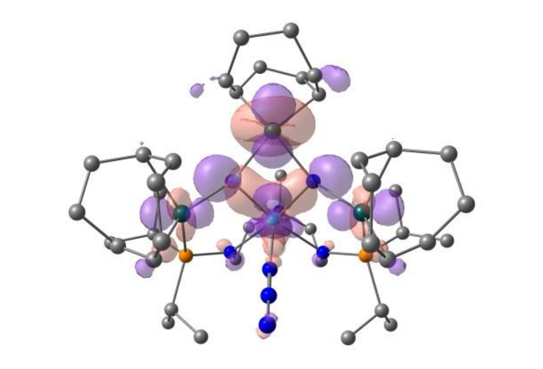
-1.34425304 eV	-4.429504248 eV
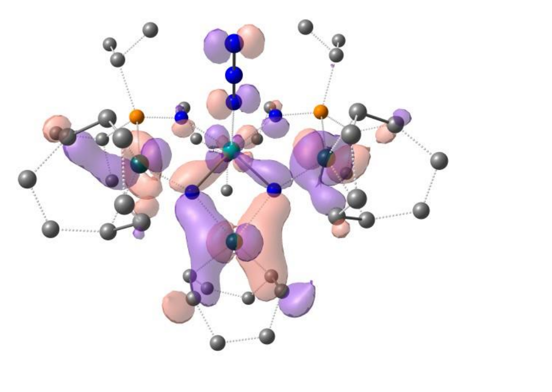	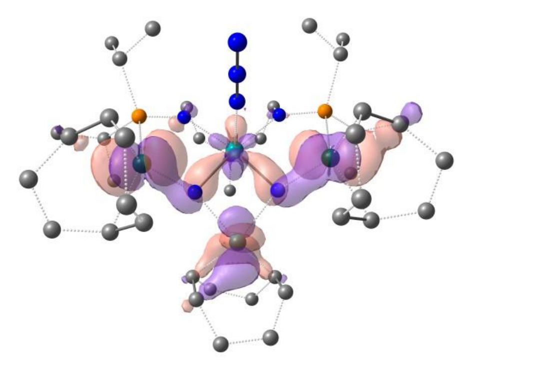

Supplementary Figure 48. Bonding U-N-Rh Molecular orbitals of complex **5a**.

Supplementary Table 15. Atomic orbital composition of the main bonding orbital from NBO analysis for complex **5a**

**1. U 1 - N 3**	(28.72%) U s(1.23%)p (2.00%)d(28.69%)f(68.04%)<br>(71.28%) N s(36.52%)p (63.36%)d(0.11%)
**2. U 1 - N 3**	(27.60%) U s(0.30%)p(2.80%)d(38.54%)f(58.34%)<br>(72.40%) N s(1.75%)p(98.16%)d (0.10%)
**3. U 1 - N 4**	(14.91%) U s(5.25%)p (9.75%)d (28.70%)f(56.25%)<br>(85.09%) N s(27.81%)p(72.13%)d (0.05%)
**4. U 1 - N 3’**	(13.43%) U s(6.69%)p (15.52%)d (44.50%)f (33.28%)<br>(86.57%) N s(33.89%)p (66.07%)d (0.04%)
**6. U 1 - N 3’**	(27.65%) U s(0.18%)p(2.55%)d(38.80%)f(58.46%)<br>(72.35%) N s(0.98%)p(98.92%)d (0.10%)
**9. Rh 1 - P 1**	(37.75%) Rh s(10.52%)p (13.44%)d (76.04%)<br>(62.25%) P s(36.77%)p (63.14%)d (0.10%)
**10.Rh 1’ - P 1’**	(38.02%) Rh s(10.76%)p (12.71%)d (76.53%)<br>(61.98%) P s(36.62%)p (63.28%)d (0.10%)

Supplementary Table 16. Composition of the NLMO associated with the U1-N3 and U1-N3' bonding NBO of complex **5a**.

98. 88.9543% U1- N3	20.110% U s(0.91%)p(0.35%)d(25.70%) f(72.99%)g(0.05%)<br>2.235% Rh1 s(49.04%)p(5.35%)d(45.62%)<br>6.367% Rh2 s(54.39%)p (2.93%)d (42.68%)<br>70.158% N3 s(43.38%)p(56.52%)d(0.10%)
99. 85.5065% U1- N3	16.447% U s(0.01%)p(0.09%)d(47.96%) f(51.92%)g (0.03%)<br>6.459% Rh1 s(8.39%)p(5.77%)d(85.84%)<br>2.216% Rh2 s(1.34%)p (7.16%)d(91.51%)<br>71.248% N3 s(0.66%)p(99.25%)d(0.09%)
103. 88.8520% U1- N3'	20.889% U s(0.81%)p (0.35%)d(24.78%) f(74.00%)g(0.05%)<br>6.388% Rh2 s(40.82%)p(3.28%)d (55.90%)<br>2.224% Rh1' s(42.33%)p(6.07%)d (51.59%)<br>69.341% N3' s(41.77%)p(58.12%)d(0.11%)
104. 85.3308% U1- N3'	17.550% U s(0.01%)p (0.08%)d(47.29%) f(52.59%)g (0.03%)<br>2.280% Rh2 s(1.85%)p(6.31%)d(91.84%)<br>6.472% Rh 1' s(6.86%)p(5.96%)d(87.18%)<br>69.794% N3' s(0.21%)p(99.69%)d (0.10%)

<u>Complex Ir 3b:</u>

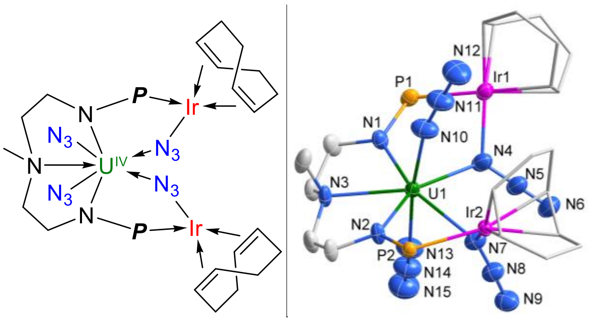

Supplementary Figure 49. Structures of complex **3b**. Unpaired spin density on U(IV) = 2.157306

LUMO	SOMO	SOMO-1
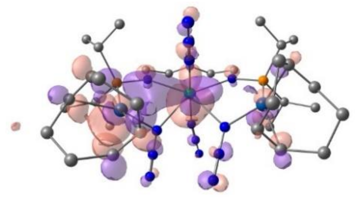	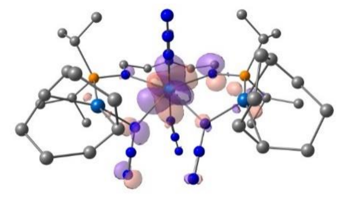	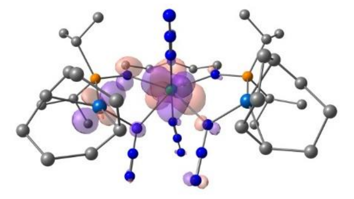
-1.603851704 eV	-4.988430512 eV	-5.010199792 eV
0.393: BD(1) U1-N7<br>-0.325: BD(1) U1-N3*<br>-0.294: BD(1) U1-N4<br>0.243: BD*(3) N8-N9*<br>-0.240: BD*(2) N5-N6*		

Supplementary Figure 50. Frontier orbitals and associated energies for complex **3b**.

Complex Ir 4b:

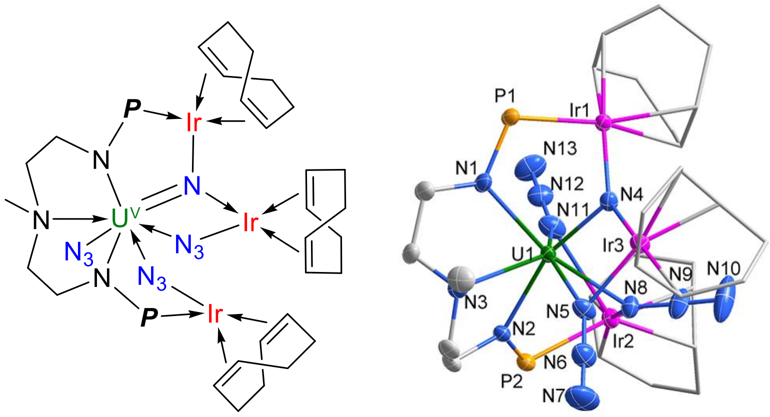

Supplementary Figure 51. Structures of complex **4b**. Unpaired spin density on U(V) = 1.241344

Supplementary Table 17. Comparison of selected bond distances (computed and experimental) for complex **4b**.

Distances	EXP	computational	Wiberg Bond Index
U1-N4	2.011(4)	1.95753	1.8346
U1-N5	2.384(4)	2.41684	0.5639
U1-N8	2.498(4)	2.50375	0.4828
U1-N11	2.386(4)	2.37141	0.6438
Ir1-N4	2.064(4)	2.10941	0.4878
Ir3-N4	2.055(4)	2.11079	0.4970
Ir3-N5	2.089(4)	2.11702	0.4310
Ir2-N8	2.094(4)	2.13794	0.4662

LUMO	SOMO
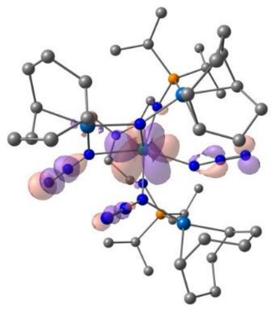	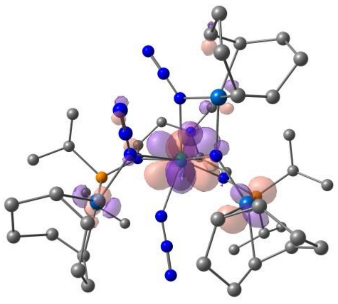
-1.717324076 eV	-5.183265568 eV
-0.304: BD(1) U1- N8<br>0.294: BD*(1) U1-N5*<br>0.288*[256]: LV (3) U1(lv)<br>-0.237*: BD*(3) N9-N10	

Supplementary Figure 52. Frontier orbitals and associated energies of complex **4b**.

Supplementary Table 18. Atomic orbital composition of the main bonding orbital from NBO analysis for complex **4b**.

**4. U 1 - N 5**	(10.97%)U s(13.90%)p(18.35%)d(34.02%)f (33.72%)<br>(89.03%)N s(39.80%)p(60.17%)d(0.03%)
**5. U 1 - N 4**	(12.76%)U s(12.17%)p(18.31%)d(38.13%)f (31.37%)<br>(87.24%)N s(70.19%)p (29.80%)d (0.00%)
**8. U 1 - N 8**	(11.03%)U s(14.85%)p(20.72%)d(32.56%)f (31.85%)<br>(88.97%)N s(40.51%)p (59.46%)d (0.03%)
**9. U 1 - N 4**	(27.75%)U s(7.50%)p(0.49%)d(32.70%)f (59.24%)<br>(72.25%)N s(32.86%)p(67.01%)d(0.13%)
**10.U 1 - N 4**	(25.45%)U s(0.72%)p(8.83%)d(33.05%)f(57.37%)<br>(74.55%)N s(3.80%)p(96.07%)d(0.13%)
**11.Ir 3 - N 5**	(23.49%) Ir s(15.03%)p(16.22%)d(68.75%)<br>(76.51%) N s(33.25%)p(66.71%)d(0.03%)
**15.Ir 2 - N 8**	(23.90%)Ir s(22.59%)p (17.58%)d (59.84%)<br>(76.10%)N s(34.12%)p (65.84%)d (0.04%)
**19.Ir 1 - N 4**	(24.63%)Ir s(31.49%)p (11.65%)d (56.86%)<br>(75.37%) N s(41.80%)p (58.17%)d (0.03%)

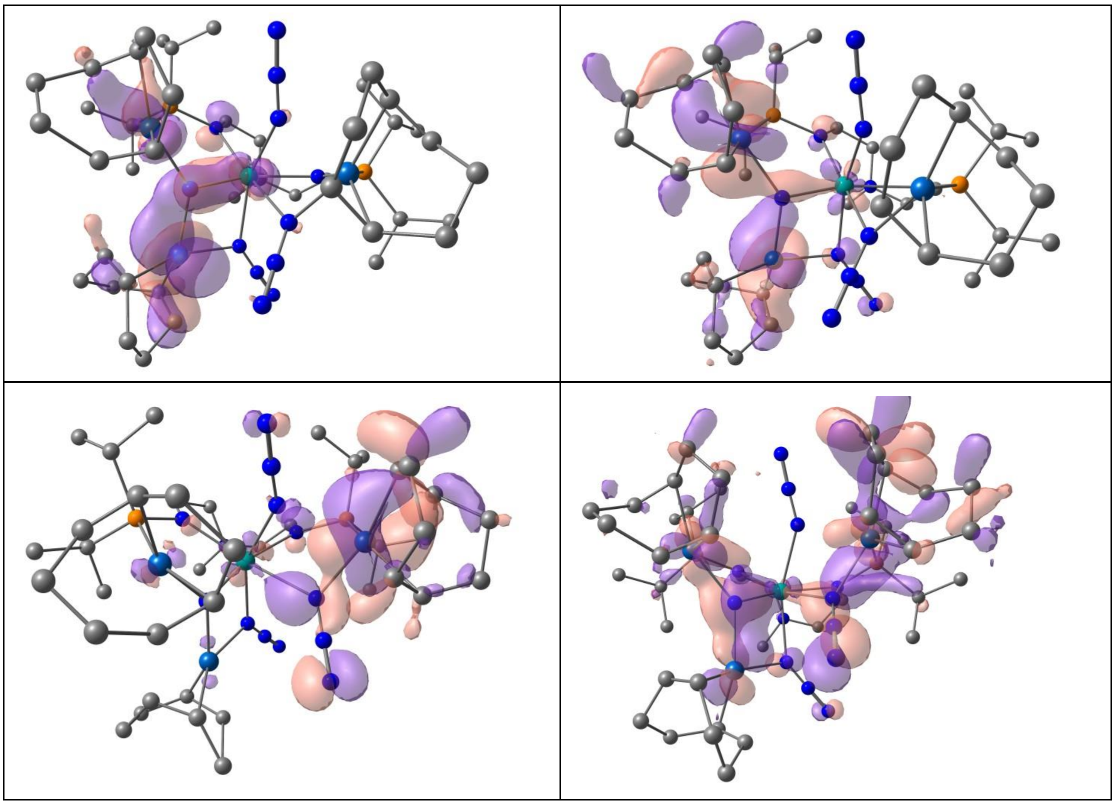

Supplementary Figure 53. Bonding U-N-Ir Molecular orbitals of complex **4b**.

Supplementary Table 19. Composition of the NLMO associated with the U1-N4 bonding NBO of complex **4b**.

108. 87.6687% U1-N4	25.266% U s(0.99%)p (0.35%)d(15.47%) f(83.16%)g(0.03%)<br>6.252% Ir 1 s(15.78%)p (2. 88%)d(81.34%)<br>2.172% Ir3 s(2.55%)p(6.36%)d(91.09%)<br>64.948% N4 s(33.59%)p (66.30%)d (0.11%)
109. 86.4537% U1-N4	22.203% U s(0.31%)p (0.08%)d(37.35%) f(62.24%)g (0.02%)<br>2.667% Ir1 s(0.11%)p(5.08%)d(94.81%)<br>6.234% Ir3 s(2.05%)p (8.97%)d(88.98%)<br>65.137% N4 s(2.93%)p(96.97%)d (0.10%)

Complex Ir 5b:

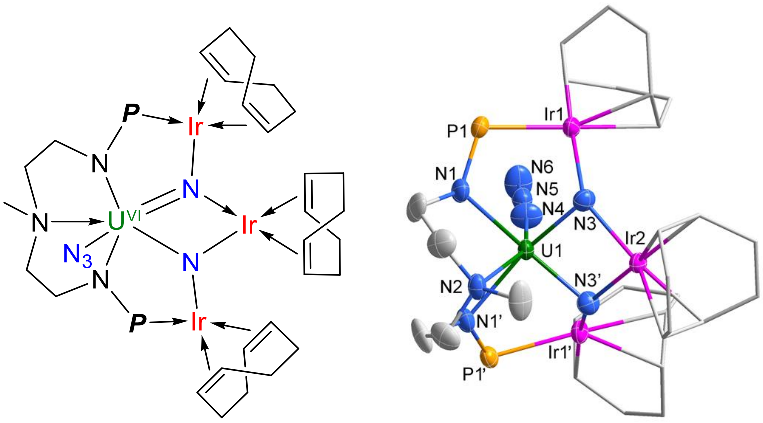

Supplementary Figure 54. Structures of complex **5b**.

Supplementary Table 20. Comparison of selected bond distances (computed and experimental) for complex **5b.**

Distances	EXP	computational	Wiberg Bond Index
U1-N3	2.005(6)	1.97696	1.8000
U1-N3’	2.005(6)	1.97514	1.8006
U1-N4	2.329(8)	2.28623	0.7938
Ir1-N3	2.059(6)	2.08279	0.5596
Ir2-N3	2.047(6)	2.08505	0.5289
Ir2-N3’	2.047(6)	2.08578	0.5255
Ir1’-N3’	2.059(6)	2.07683	0.5595

LUMO	HOMO
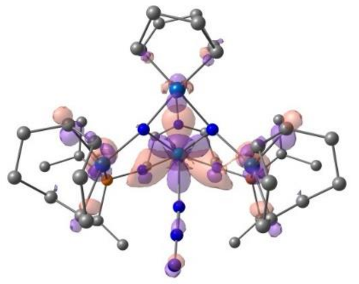	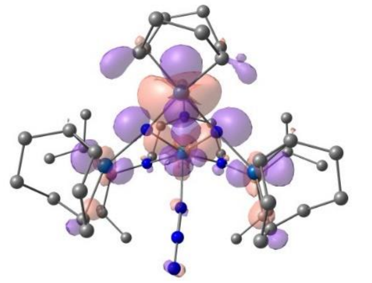
-1.631879652 eV	-4.444198512 eV

Supplementary Figure 55. Frontier orbitals and associated energies of complex **5b.**

Supplementary Table 21. Atomic orbital composition of the main bonding orbital from NBO analysis for complex **5b**.

**4. U 1 - N 3**	(28.22%)U s(3.12%)p (0.32%)d(37.69%)f(58.81%)<br>(71.78%) N s(30.79%)p(69.11%)d(0.10%)
**5.U 1 - N 3**	(25.51%)U s(0.17%)p(10.91%)d(39.33%)f(49.55%)<br>(74.49%) N s(0.20%)p(99.71%)d(0.09%)
**6. U 1 - N 4**	(14.59%)U s(6.74%)p(18.47%)d(45.96%)f (28.80%)<br>(85.41%)N s(70.49%)p(29.51%)d (0.00%)
**9. U 1 - N 3'**	(28.13%)U s(3.26%)p (0.49%)d(36.89%)f(59.30%)<br>(71.87%)N s(31.76%)p(68.14%)d (0.10%)
**10.U 1 - N 3'**	(25.26%)U s(0.10%)p(11.12%)d(40.64%)f(48.11%)<br>(74.74%)N s(0.22%)p(99.69%)d (0.09%)
**12.Ir 1 - N 3**	(28.18%)Ir s(25.03%)p(11.12%)d(63.85%)<br>(71.82%) N s(35.46%)p(64.50%)d(0.04%)
**15.Ir 2 - N 3**	(26.01%)Ir s(31.53%)p(13.52%)d(54.96%)<br>(73.99%)N s(33.49%)p(66.47%)d (0.05%)
**16.Ir 2 - N 3'**	(24.80%) Ir s(34.21%)p(15.68%)d (50.11%)<br>(75.20%) N s(32.78%)p(67.17%)d(0.05%)
**20.Ir 1' - N 4**	(28.11%) Ir s(25.82%)p(10.63%)d (63.55%)<br>(71.89%) N s(35.18%)p(64.78%)d (0.04%)

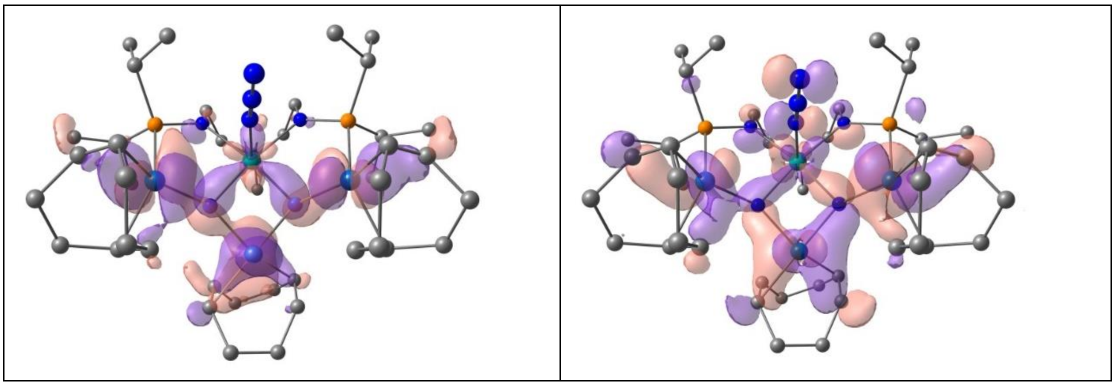

Supplementary Figure 56. Bonding U-N-Ir Molecular orbitals of complex **5b**.

Supplementary Table 22. Composition of the NLMO associated with the U1-N3 and U1-N3' bonding NBO of complex **5b**.

93. 88.1743% U1- N3	24.766% U s(0.59%)p (0.17%)d(20.00%) f(79.20%)g (0.03%)<br>2.146% Ir1 s(17.93%)p (6.54%)d (75.53%)<br>6.314% Ir2 s(22.73%)p (3.42%)d (73.85%)<br>65.392% N3 s(33.85%)p(66.06%)d(0.09%)
94. 85.6659% U1- N3	21.326% U s(0.04%)p (0.10%)d(40.63%) f(59.22%)g (0.02%)<br>6.710% Ir1 s(9.16%)p (4.92%)d(85.92%)<br>2.393% Ir2 s(5.09%)p(5.25%)d(89.66%)<br>64.996% N3 s(0.18%)p(99.74%)d(0.09%)
98. 88.5702% U1- N3'	24.575% U s(0.65%)p(0.19%)d(21.39%)f(77.74%)g (0.03%)<br>6.303% Ir2 s(22.67%)p (3.91%)d(73.42%)<br>2.137% Ir1' s(14.86%)p (6.93%)d(78.21%)<br>65.711% N3' s(34.62%)p(65.29%)d(0.09%)
99. 85.5759% U1- N3'	21.094% U s(0.05%)p(0.10%)d(40.09%)f(59.74%)g(0.02%)<br>2.382% Ir2 s(5.89%)p(4.76%)d(89.35%)<br>6.691% Ir1' s(8.08%)p(5.39%)d(86.52%)<br>65.220% N3' s(0.15%)p(99.76%)d (0.09%)

Supplementary References

1. Kahn, O. Molecular Magnetism, VCH Publishers, Inc., New York, 1993.

2. Xin, X., Douair, I., Zhao, Y., Wang, S., Maron, L. & Zhu, C. Dinitrogen Cleavage by a Multimetallic Cluster Featuring Uranium–Rhodium Bond. *J. Am. Chem. Soc.* **142**, 15004–15011 (2020).

3. Sheldrick, G. M. Crystal structure refinement with SHELXL. *Acta Crystallogr. Sect. C* **71**, 3–8 (2015)

4. Dolomanov, O. V., Bourhis, L. J., Gildea, R. J., Howard, J. A. K. & Puschmann, H. OLEX2: A complete structure solution, refinement and analysis program. *J. Appl. Crystallogr.* **42**, 339–341 (2009).

5. Gaussian 09, Revision D.01, Frisch, M. J., Trucks, G. W., Schlegel, H. B., Scuseria, G. E., Robb, M. A., Cheeseman, J. R., Scalmani, G., Barone, V., Petersson, G. A., Nakatsuji, H., Li, X., Caricato, M., Marenich, A., Bloino, J., Janesko, B. G., Gomperts, R., Mennucci, B., Hratchian, H. P., Ortiz, J. V., Izmaylov, A. F., Sonnenberg, J. L., Williams-Young, D., Ding, F., Lipparini, F., Egidi, F., Goings, J., Peng, B., Petrone, A., Henderson, T., Ranasinghe, D., Zakrzewski, V. G., Gao, J., Rega, N., Zheng, G., Liang, W., Hada, M., Ehara, M., Toyota, K., Fukuda, R., Hasegawa, J., Ishida, M., Nakajima, T., Honda, Y., Kitao, O., Nakai, H., Vreven, T., Throssell, K., Montgomery, J. A., Peralta, Jr., J. E., Ogliaro, F., Bearpark, M., Heyd, J. J., Brothers, E., Kudin, K. N., Staroverov, V. N., Keith, T., Kobayashi, R., Normand, J., Raghavachari, K., Rendell, A., Burant, J. C., Iyengar, S. S., Tomasi, J., Cossi, M., Millam, J. M., Klene, M., Adamo, C., Cammi, R., Ochterski, J. W., Martin, R. L., Morokuma, K., Farkas, O., Foresman, J. B. & Fox, D. J. Gaussian, Inc., Wallingford CT (2016).

6. Becke, A.D. Density-functional thermochemistry. III. The role of exact Exchange. *J. Chem. Phys.* **98**, 5648–5652 (1993).

7. Burke, K., Perdew, J. P. & Wang, Y. In Electronic Density Functional Theory: Recent Progress and New Directions. Dobson, J. F., Vignale, G. & Das, M. P. Eds., Plenum: New York, 1998.

8. Küchle, W., Dolg, M., Stoll, H. & Preuss, H. Energy-adjusted pseudopotentials for the actinides. Parameter sets and test calculations for thorium and thorium monoxide. *J. Chem. Phys.* **100**, 7535–7542 (1994).

9. Cao, X., Dolg, M. & Stoll, H. Valence basis sets for relativistic energyconsistent small-core actinide pseudopotentials. *J. Chem. Phys.* **118**, 487–496 (2003).

10. Cao, X. & Dolg, M. Segmented contraction scheme for small-core actinide pseudopotential basis set. *J. Mol. Struct: Theochem* **673**, 203–209 (2004).

11. Höllwarth, A., Böhme, M., Dapprich, S., Ehlers, A.W., Gobbi, A., Jonas, V., Köhler, K. F., Stegmann, R., Veldkamp, A. & Frenking, G. A set of d-polarization functions for pseudo-potential basis sets of the main group elements Al-Bi and f-type polarization functions for Zn, Cd, Hg. *Chem. Phys. Lett.* **208**, 237–240 (1993).

12. McLean, A. D. & Chandler, G. S. Contracted Gaussian basis sets for molecular calculations. I. Second row atoms, Z=11–18. *J. Chem. Phys.* **72**, 5639–5648 (1980).

13. Hehre, W. J., Ditchfield, R. & Pople, J. A. Self-consistent molecular orbital methods. XII. Further extensions of Gaussian-type basis sets for use in molecular orbital studies of organic molecules. *J. Chem. Phys.* **56**, 2257–2261 (1972).
