# Supplementary material for: Photochemical Synthesis of Transition Metal-Stabilized Uranium(VI) Nitride Complexes
Source: Nat Commun. 2022 Jul 1;13:3809. doi: 10.1038/s41467-022-31582-z (PMC9249861; doi:10.1038/s41467-022-31582-z)
Supplement: Supplementary file 2 — Description of Additional Supplementary Files [file 41467_2022_31582_MOESM2_ESM.pdf]

Supplementary Data File 1: Cartesian coordinates of the optimized structures
