# Supplementary material for: Photochemical Synthesis of Transition Metal-Stabilized Uranium(VI) Nitride Complexes
Source: Nat Commun. 2022 Jul 1;13:3809. doi: 10.1038/s41467-022-31582-z (PMC9249861; doi:10.1038/s41467-022-31582-z)
Supplement: Supplementary file 3 — Supplementary Data 1 [file 41467_2022_31582_MOESM3_ESM.pdf]

Cartesian coordinates of all optimized structures

116

Complex 3a Entalpy (a.u) = -2826.491813

U 10.314443 9.864864 9.703125
Rh 11.408005 12.004927 6.587664
Rh 6.607920 10.557996 9.821893
P 13.171387 11.219235 7.981856
P 7.482385 9.732243 11.877464
N 9.270109 14.384659 9.971273
N 11.033857 5.506463 8.387655
N 7.255592 7.297455 7.865813
N 8.964175 9.140371 5.676588
N 10.833014 6.563254 8.793875
N 7.618388 8.260297 8.370502
N 9.661477 9.677051 6.412640
N 9.564256 13.267257 9.957411
N 10.633315 7.667519 9.235939
N 11.880234 9.269187 11.734198
N 9.868280 12.108344 9.950479
N 8.025171 9.277773 8.907252
N 9.154297 9.388627 11.737001
N 12.632185 10.298662 9.322645
N 10.413552 10.236694 7.193432
C 6.229225 13.085212 8.178450
C 11.395725 12.603828 3.611676
C 3.912146 10.975557 8.320628
C 9.617023 14.337004 5.539555
C 12.294641 13.718929 4.167706
C 10.616449 14.908131 6.561193
C 15.814996 12.180029 8.866482
C 11.162070 8.248325 12.507972
C 5.429295 13.391798 9.456879
C 13.567250 8.767277 6.644175
C 3.615368 10.837888 9.824661
C 14.840461 10.733358 5.710448
C 13.692452 9.711694 10.140147
C 13.103087 8.721615 11.133532
C 9.644044 12.822146 5.503340
C 13.748874 13.272226 9.830186
C 12.165193 10.456920 12.546764
C 14.298251 10.067786 6.975333
C 7.021564 6.977058 11.449892
C 8.202353 12.039627 13.319532

C	5.144650	8.225738	12.575504
C	11.869268	14.074389	6.700136
C	12.615848	13.515322	5.642240
C	7.179391	10.237293	14.762668
C	4.783728	11.217364	10.708119
C	14.357715	12.576892	8.608945
C	10.422889	12.063431	4.633272
C	5.622776	12.333776	10.532939
C	5.357421	10.657686	7.990024
C	9.781085	8.754589	12.893464
C	6.368580	11.607893	7.903616
C	7.203299	10.881975	13.373086
C	6.659838	8.106173	12.413736
H	5.778345	13.594351	7.312683
H	7.235328	13.500580	8.289620
H	10.854507	12.947529	2.716888
H	12.022721	11.767658	3.283367
H	3.261080	10.295259	7.761714
H	3.667159	11.983226	7.970498
H	8.607342	14.669194	5.802160
H	9.812205	14.733678	4.537952
H	13.229257	13.744784	3.597086
H	11.831073	14.700593	4.023900
H	10.879757	15.946300	6.304275
H	10.138791	14.951852	7.545704
H	16.307968	11.779949	7.976775
H	16.382784	13.065254	9.179347
H	15.910021	11.437823	9.663452
H	11.057094	7.372448	11.860967
H	11.733282	7.952426	13.404975
H	5.752889	14.360261	9.850915
H	4.362126	13.496761	9.233185
H	12.734852	8.946492	5.956494
H	14.256071	8.062401	6.163833
H	13.154467	8.292285	7.537000
H	2.724842	11.425886	10.096523
H	3.364988	9.793126	10.041962
H	15.395771	11.654622	5.915965
H	15.523141	10.049691	5.191600
H	14.024375	10.977899	5.022941
H	14.441777	9.163780	9.548151
H	14.256278	10.479401	10.693932
H	13.846901	8.462812	11.907108
H	12.817824	7.804792	10.610842

H 8.800698 12.320526 5.973937
H 13.852184 12.653878 10.726682
H 14.266645 14.218860 10.024944
H 12.683588 13.484217 9.704811
H 11.232551 10.880401 12.921075
H 12.819934 10.214743 13.399281
H 12.644708 11.217826 11.930492
H 15.144526 9.835203 7.634643
H 8.093836 6.941291 11.239842
H 6.719765 6.011444 11.872587
H 6.503040 7.091419 10.493442
H 8.257371 12.495737 12.328122
H 7.922759 12.816664 14.040721
H 9.210489 11.699268 13.573795
H 4.665422 8.418525 11.610784
H 4.735946 7.284806 12.963166
H 4.847010 9.019468 13.268625
H 12.398082 14.249011 7.636130
H 13.663357 13.301334 5.841427
H 8.145407 9.801667 15.033643
H 6.948474 11.002029 15.514706
H 6.421536 9.455200 14.856446
H 4.705315 10.809980 11.714937
H 14.362648 13.298501 7.781810
H 10.109119 11.036226 4.449561
H 6.170497 12.676784 11.408398
H 5.535384 9.694061 7.514495
H 9.871375 9.445217 13.747842
H 9.217117 7.888080 13.271201
H 7.267814 11.315298 7.364795
H 6.201748 11.292681 13.184581
H 7.092637 7.879357 13.396282

116
Complex 3b Enthalpy (a.u) = -2814.142918
U 10.340545 5.005564 9.670484
Ir 11.422442 2.856502 6.565016
Ir 6.622561 4.322550 9.803675
P 13.193530 3.628919 7.966305
P 7.510772 5.152499 11.856812
N 11.128666 9.320273 8.272439
N 9.284856 0.501188 9.940287
N 8.950929 5.714274 5.640670
N 10.889877 8.281968 8.704668

N 7.311101 7.577210 7.797087
N 9.658909 5.180067 6.365251
N 9.592726 1.613923 9.933098
N 11.903693 5.626842 11.690557
N 7.644964 6.611820 8.313696
N 10.647766 7.197642 9.174535
N 10.430755 4.632947 7.140615
N 9.913807 2.769100 9.935773
N 12.655752 4.575647 9.289323
N 9.177770 5.507227 11.698558
N 8.033960 5.594819 8.869145
C 11.419535 2.237816 3.581156
C 9.618330 0.533145 5.497065
C 10.612015 -0.064999 6.508400
C 6.235091 1.779840 8.151337
C 12.315894 1.126694 4.149323
C 3.921405 3.884561 8.315391
C 15.800697 2.622268 8.892096
C 11.853091 0.783174 6.684188
C 11.186271 6.658290 12.451352
C 13.717031 5.161962 10.105571
C 13.638034 6.051380 6.598444
C 5.454267 1.469470 9.439585
C 13.701899 1.572621 9.831010
C 14.904813 4.059549 5.711715
C 13.129415 6.164939 11.086405
C 3.619699 3.989883 9.821381
C 7.014629 7.896244 11.408153
C 12.185957 4.448750 12.518598
C 10.469267 2.806769 4.610410
C 9.806528 6.156048 12.845894
C 12.624633 1.346455 5.627037
C 9.668886 2.050524 5.485296
C 8.244009 2.845309 13.284373
C 14.339682 2.252500 8.616023
C 4.810288 3.649918 10.694395
C 14.349387 4.747583 6.958735
C 7.230427 4.644785 14.739548
C 5.368441 4.219472 7.994533
C 5.162587 6.634860 12.561837
C 6.389169 3.260930 7.901241
C 7.244776 4.001836 13.349190
C 6.674767 6.771793 12.385626
C 5.668359 2.532311 10.510734

H	12.047085	3.062357	3.225223
H	10.857835	1.880459	2.704888
H	8.603820	0.212316	5.754674
H	9.806962	0.149171	4.488936
H	10.126782	-0.141327	7.487293
H	10.889311	-1.091004	6.220885
H	7.238330	1.352003	8.238200
H	5.761760	1.292975	7.285055
H	11.853472	0.143609	4.010418
H	13.254507	1.096247	3.585720
H	3.267611	4.570219	7.766619
H	3.685728	2.881861	7.944518
H	15.899223	3.356385	9.696125
H	16.348739	1.724874	9.205107
H	16.311335	3.020178	8.011519
H	12.382418	0.566376	7.612222
H	11.758786	6.966117	13.343324
H	11.080051	7.525273	11.792657
H	14.272267	4.395072	10.668926
H	14.473019	5.698613	9.511921
H	13.221250	6.546217	7.478615
H	14.340590	6.740045	6.114739
H	12.811867	5.868968	5.904083
H	4.383552	1.365361	9.232285
H	5.782964	0.501115	9.829393
H	12.636383	1.373944	9.688918
H	14.204201	0.620403	10.038158
H	13.799925	2.194282	10.725945
H	14.097345	3.815236	5.014645
H	15.603570	4.730153	5.197294
H	15.446625	3.135238	5.937469
H	12.848405	7.077144	10.553338
H	13.871912	6.429987	11.858929
H	2.756997	3.360252	10.087620
H	3.327165	5.020397	10.053857
H	6.487205	7.765512	10.458564
H	6.705221	8.861938	11.824918
H	8.084350	7.943059	11.187418
H	12.665214	3.679554	11.912691
H	12.839775	4.701081	13.368722
H	11.252367	4.030968	12.896573
H	10.139951	3.822284	4.387589
H	9.242552	7.026650	13.214072
H	9.899881	5.476128	13.708529

H 13.681385 1.509678 5.829630
H 8.799532 2.551173 5.908463
H 9.252930 3.185353 13.536244
H 7.968274 2.063868 14.002121
H 8.293389 2.396567 12.289452
H 14.339253 1.530211 7.789842
H 4.704174 4.027290 11.711326
H 15.186939 4.979261 7.629409
H 6.474524 5.428045 14.839278
H 7.002851 3.879159 15.491542
H 8.198865 5.078127 15.005488
H 5.526592 5.163853 7.474205
H 4.881679 5.843157 13.263917
H 4.746389 7.574016 12.945668
H 4.678397 6.426825 11.602921
H 7.265561 3.541382 7.318815
H 6.241984 3.593378 13.164321
H 7.114714 7.011289 13.362006
H 6.189806 2.168664 11.394731

135

Complex 4a Enthalpy (a.u) = -3139.506968

92	10.343275494	11.034896561	4.359234451
45	8.815400000	8.385327000	3.278965000
45	8.356138000	12.704884000	7.189172000
45	8.500183000	11.976158000	1.632423000
15	10.803771000	12.049190000	1.146851000
15	10.691142000	12.342988000	7.620095000
7	6.743356000	9.288649000	6.154408000
7	11.032298000	7.012620000	6.289280000
7	9.321059494	15.525914561	4.044927451
7	10.655631000	7.810271000	5.552682000
7	7.587573000	10.067517000	6.124795000
7	12.987591176	10.118617588	4.404527213
7	9.493478494	14.405661561	4.290793451
7	9.671517494	13.259431561	4.577467451
7	11.688092000	11.660893000	2.555437000
7	11.500214000	11.486499000	6.352288000
7	10.284382000	8.662113000	4.774577000
7	8.492596000	10.877621000	6.113306000
7	9.000926330	10.495764851	3.072078336
6	6.075570000	7.133289000	3.382241000
6	6.815761000	14.568942000	1.663602000
6	6.978316000	5.917725000	3.638502000

6	12.745945000	13.584954000	-0.445967000
6	12.955820000	13.705992000	8.921021000
6	7.877417000	6.798924000	0.828149000
6	13.717152000	11.255256000	3.812607000
6	10.224620000	11.874962000	10.396477000
6	11.696891000	14.713546000	1.548995000
6	6.937087000	14.061428000	9.590669000
6	6.754656000	12.567980000	-0.795905000
6	13.324435176	8.889146588	3.686426213
6	9.129443000	6.088193000	1.376775000
6	10.895045000	11.258232000	9.168703000
6	8.249258000	14.806285000	7.489458000
6	10.365625000	9.849163000	8.909911000
6	8.036550000	13.969205000	0.980008000
6	7.062675000	15.395530000	6.760519000
6	6.203333000	12.736801000	9.334783000
6	13.357445000	9.948043000	5.822080000
6	11.097359000	9.362249000	0.300333000
6	12.083837000	14.647133000	6.747425000
6	12.887728000	11.130953000	6.648563000
6	6.307407000	13.709431000	2.836891000
6	10.548957000	11.021343000	-1.502024000
6	5.684894000	12.136376000	0.217991000
6	6.380988000	12.234314000	7.919966000
6	11.722536000	13.897312000	8.031673000
6	5.833817000	14.468556000	6.755900000
6	11.493532000	13.685243000	0.433990000
6	8.002980000	13.103341000	-0.132614000
6	11.299578000	10.791628000	-0.189576000
6	8.202522000	14.182625000	8.752316000
6	8.418179000	6.347874000	3.862036000
6	9.386209000	6.399646000	2.837158000
6	6.804973000	8.255248000	2.676435000
6	7.623520000	8.124743000	1.532770000
6	6.302561000	11.538284000	1.465696000
6	13.136558000	11.596378000	2.451925000
6	6.226032000	13.001829000	6.756893000
6	6.543620000	12.232562000	2.640918000
1	5.174577000	6.847305000	2.816714000
1	5.731003000	7.529224000	4.342156000
1	7.085017000	15.557609000	2.046930000
1	6.021476000	14.731469000	0.926694000
1	6.621161000	5.375813000	4.520524000
1	6.926242000	5.206648000	2.806481000

1	12.606070000	12.945712000	-1.321068000
1	13.004848000	14.584705000	-0.816167000
1	13.615090000	13.212703000	0.103928000
1	13.732245000	13.112456000	8.430898000
1	13.394165000	14.686354000	9.145256000
1	12.723683000	13.231990000	9.878222000
1	6.995418000	6.155691000	0.922609000
1	8.002994000	6.976650000	-0.245576000
1	13.562316000	12.116641000	4.468307000
1	14.805789000	11.077436000	3.741792000
1	9.142110000	11.939480000	10.252325000
1	10.410770000	11.245766000	11.275213000
1	10.596622000	12.878094000	10.629587000
1	12.484115000	14.405894000	2.243293000
1	11.993065000	15.676428000	1.115499000
1	10.794193000	14.883981000	2.137991000
1	7.200446000	14.132682000	10.651684000
1	6.281292000	14.915110000	9.388209000
1	6.342167000	13.305281000	-1.502912000
1	7.043867000	11.699367000	-1.400475000
1	13.005329176	8.953444588	2.646228213
1	14.411031176	8.697812588	3.708244213
1	12.815876176	8.039544588	4.144420213
1	10.005231000	6.424859000	0.811346000
1	9.062043000	5.000463000	1.218117000
1	11.972369000	11.197143000	9.365630000
1	9.206467000	15.223923000	7.175596000
1	10.857081000	9.375679000	8.057530000
1	10.533091000	9.218786000	9.791339000
1	9.290926000	9.866411000	8.704487000
1	8.939692000	14.560342000	1.110781000
1	6.808734000	16.377993000	7.188995000
1	7.377542000	15.592477000	5.730805000
1	5.132552000	12.828685000	9.574233000
1	6.599271000	11.968994000	10.008711000
1	12.864049000	9.038789000	6.178091000
1	14.444952000	9.806410000	5.967501000
1	10.037810000	9.144473000	0.455111000
1	11.495267000	8.651856000	-0.435489000
1	11.593724000	9.185116000	1.256337000
1	11.206978000	14.883370000	6.142285000
1	12.587618000	15.589126000	6.994544000
1	12.761199000	14.060007000	6.120082000
1	13.567481000	11.983348000	6.490636000

1	13.028835000	10.839403000	7.698605000
1	5.238463000	13.904720000	3.017023000
1	6.832805000	14.010215000	3.747287000
1	10.676653000	12.032833000	-1.900450000
1	10.907090000	10.321534000	-2.267224000
1	9.476925000	10.847064000	-1.365044000
1	5.024695000	11.392335000	-0.241830000
1	5.044093000	12.981539000	0.486469000
1	6.271790000	11.154448000	7.821235000
1	11.011021000	14.519721000	8.590926000
1	5.230418000	14.677276000	5.866422000
1	5.183609000	14.677921000	7.612530000
1	10.676030000	14.044009000	-0.205677000
1	8.859081000	13.136759000	-0.805441000
1	12.371065000	10.955013000	-0.361486000
1	9.121306000	14.181193000	9.332568000
1	8.779843000	6.227149000	4.882436000
1	10.431122000	6.294339000	3.137062000
1	6.385029000	9.236347000	2.886781000
1	7.770356000	9.019046000	0.927821000
1	6.282265000	10.455446000	1.511736000
1	13.469643000	10.855661000	1.704845000
1	13.587909000	12.551400000	2.144600000
1	6.042086000	12.460058000	5.830514000
1	6.698969000	11.650678000	3.547157000

135

Complex 4b Enthalpy (a.u) = -3120.985761

92	12.962539295	11.409637060	13.216194169
77	11.404991000	8.801898000	12.112508000
77	11.032365000	13.169949000	15.913298000
77	11.093205000	12.392032000	10.484007000
15	13.401174000	12.502184000	10.007978000
15	13.351149000	12.722796000	16.441239000
7	13.528503000	7.414136000	15.197047000
7	9.369374000	9.674172000	15.102328000
7	13.195905000	8.196259000	14.427314000
7	11.927124295	15.844013060	12.899845169
7	15.560650705	10.472531940	13.197476831
7	10.202177000	10.457533000	15.015583000
7	14.277578000	12.097801000	11.421578000
7	12.061712295	14.745332060	13.232309169
7	12.875158000	9.043434000	13.616516000
7	12.205158295	13.619180060	13.625925169
7	14.127894000	11.817484000	15.176504000

7	11.107690000	11.267179000	14.941394000
7	11.584967000	10.892521000	11.883739000
6	9.543406000	6.336288000	12.458395000
6	16.312289000	11.619379000	12.648609000
6	11.706205000	6.475966000	10.212128000
6	8.645220000	7.549653000	12.180892000
6	15.679485000	14.034069000	17.688722000
6	15.726816000	12.027645000	11.309060000
6	15.336513000	14.073511000	8.454301000
6	15.939755000	10.240161000	14.606288000
6	15.878518705	9.265653940	12.433334831
6	12.896928000	12.356142000	19.216265000
6	13.155022000	11.537788000	7.339934000
6	10.981057000	6.775076000	12.698329000
6	9.739560000	14.565420000	18.391928000
6	9.373999000	14.975297000	10.570470000
6	14.820250000	14.953410000	15.499644000
6	13.539028000	11.671158000	18.009312000
6	14.273684000	15.162638000	10.462480000
6	9.336030000	13.055344000	8.060362000
6	9.017172000	13.222940000	18.225893000
6	12.946638000	10.280281000	17.794098000
6	13.897766000	11.270586000	8.650473000
6	10.472176000	7.206516000	9.649931000
6	15.497693000	11.403014000	15.475312000
6	13.678836000	9.830911000	9.102492000
6	11.965834000	6.814636000	11.667853000
6	8.281947000	12.564581000	9.063349000
6	14.446449000	14.246004000	16.803813000
6	10.940837000	14.672231000	17.457561000
6	14.079516000	14.152884000	9.329794000
6	9.397648000	8.674981000	11.500991000
6	8.424498000	14.829972000	15.626968000
6	8.862254000	14.083169000	11.717029000
6	10.235672000	8.539375000	10.353772000
6	9.597070000	15.823925000	15.555250000
6	10.589325000	13.555031000	8.744943000
6	8.911092000	13.384480000	15.623996000
6	8.920324000	11.940764000	10.291864000
6	9.190183000	12.660723000	16.830652000
6	10.611683000	14.397044000	9.892960000
6	10.869782000	15.275571000	16.171256000
6	9.144959000	12.617386000	11.495300000
1	9.175185000	5.799649000	13.338979000

1	9.503063000	5.619075000	11.630845000
1	16.177145000	12.453164000	13.342730000
1	17.396782000	11.426995000	12.560986000
1	11.614272000	5.387513000	10.075504000
1	12.591280000	6.782641000	9.643329000
1	7.761541000	7.267657000	11.587491000
1	8.272702000	7.942768000	13.132042000
1	16.430305000	13.398820000	17.211054000
1	16.155182000	15.003446000	17.882650000
1	15.437882000	13.595605000	18.660255000
1	16.048920000	11.319858000	10.527005000
1	16.181966000	12.993075000	11.042821000
1	15.206750000	13.445481000	7.569814000
1	15.588740000	15.080461000	8.099870000
1	16.205873000	13.701477000	9.004110000
1	15.431540000	9.327836000	14.930922000
1	17.024517000	10.071552000	14.739937000
1	15.594067705	9.388091940	11.388538831
1	16.956735705	9.036567940	12.477777831
1	15.324912705	8.415748940	12.835355831
1	11.819976000	12.468155000	19.062796000
1	13.049721000	11.745751000	20.114328000
1	13.315472000	13.347266000	19.419406000
1	13.290457000	12.557177000	6.965549000
1	13.513733000	10.853266000	6.561517000
1	12.081689000	11.366898000	7.468846000
1	11.338532000	6.603082000	13.713666000
1	10.077783000	14.682204000	19.427401000
1	9.057692000	15.402022000	18.203933000
1	9.628144000	15.958789000	10.976613000
1	8.587873000	15.145599000	9.826534000
1	13.943868000	15.202345000	14.899384000
1	15.355374000	15.884708000	15.719760000
1	15.474059000	14.331342000	14.880516000
1	14.614344000	11.572552000	18.202194000
1	15.065113000	14.850375000	11.150245000
1	14.560403000	16.135619000	10.045839000
1	13.368991000	15.312918000	11.053283000
1	8.913802000	13.829560000	7.400945000
1	9.622938000	12.222437000	7.406327000
1	7.948602000	13.313421000	18.473992000
1	9.432918000	12.494298000	18.931235000
1	13.418618000	9.755896000	16.960221000
1	13.080824000	9.671534000	18.696088000

1	11.874663000	10.344115000	17.583934000
1	14.971338000	11.430577000	8.488836000
1	10.610059000	7.382492000	8.577489000
1	9.578008000	6.578894000	9.735878000
1	16.206291000	12.237960000	15.358131000
1	15.612910000	11.067918000	16.515446000
1	12.615511000	9.614902000	9.230766000
1	14.087700000	9.137326000	8.356952000
1	14.153876000	9.624999000	10.063343000
1	13.002950000	6.652475000	11.973500000
1	7.637637000	11.818878000	8.584524000
1	7.621191000	13.384043000	9.362275000
1	13.762688000	14.907480000	17.351845000
1	11.891311000	14.751257000	17.978669000
1	13.260160000	14.514748000	8.694629000
1	8.939155000	9.646392000	11.674511000
1	7.763628000	14.990722000	14.768798000
1	7.805765000	15.017481000	16.512906000
1	7.784250000	14.242001000	11.875903000
1	9.356865000	14.375341000	12.647412000
1	10.346598000	9.423121000	9.724816000
1	9.335785000	16.786188000	16.022993000
1	9.817927000	16.051700000	14.507114000
1	11.430739000	13.647809000	8.057865000
1	8.574551000	12.789043000	14.775086000
1	8.864645000	10.858720000	10.325727000
1	9.039618000	11.580731000	16.794184000
1	11.494381000	15.020524000	10.018767000
1	11.776931000	15.759037000	15.802859000
1	9.249852000	12.017974000	12.398084000

130

Complex 5a Enthalpy (a.u) = -2865.933591

92	7.187553086	4.999910214	5.868884252
45	8.093829719	7.728717073	7.372509446
45	10.159493000	4.984626000	5.473424000
45	8.085342738	2.271896470	7.376827781
15	6.045476000	8.067455000	6.268718000
15	6.046705000	1.937363000	6.278910000
7	5.693711000	6.672556000	5.348205000
7	6.368778457	4.988850242	3.248757520
7	8.621698000	6.330299000	5.954901000
7	6.539269086	5.025695214	8.076013252
7	6.324612086	4.992727214	9.248890252
7	6.094670086	4.965168214	10.386189252

7	5.695505000	3.346621000	5.381970000
7	8.606979000	3.686468000	6.012761000
6	4.803713000	6.667512000	4.199887000
6	5.573629000	6.231279000	2.954091000
6	7.490282457	4.941604242	2.301534520
6	6.278057000	9.485160000	5.023982000
6	7.567026000	9.291957000	4.223697000
6	6.258560000	10.867748000	5.678519000
6	4.453438000	8.550050000	7.188719000
6	3.296905000	8.991445000	6.285592000
6	3.998846000	7.407182000	8.097560000
6	10.035626719	8.673339073	7.425736446
6	10.097134719	7.582606073	8.297351446
6	10.037069719	7.702300073	9.807067446
6	8.593041719	7.598086073	10.333162446
6	7.562997719	8.173645073	9.386963446
6	7.657520719	9.407119073	8.714497446
6	8.822811719	10.371418073	8.876138446
6	9.919693719	10.125927073	7.829056446
6	11.763395000	6.413021000	5.326559000
6	11.146307000	6.268442000	4.070343000
6	11.764398000	5.519918000	2.897534000
6	13.086551000	5.804851000	5.733729000
6	4.760062000	3.387625000	4.271473000
6	5.494470000	3.789541000	2.990950000
6	6.259283000	0.542050000	5.005419000
6	7.534765000	0.754374000	4.188784000
6	6.255498000	-0.854557000	5.629671000
6	4.467549000	1.448476000	7.216650000
6	3.301694000	0.981782000	6.338316000
6	4.013477000	2.598188000	8.117517000
6	11.859717000	3.625185000	5.358150000
6	11.035603000	3.476267000	4.231091000
6	11.345638000	4.036115000	2.859181000
6	13.206328000	4.329305000	5.325269000
6	10.078514738	1.433609470	7.437389781
6	10.061782738	2.486571470	8.354011781
6	9.976833738	2.304595470	9.855838781
6	8.519283738	2.319409470	10.354192781
6	7.537930738	1.730010470	9.365843781
6	7.707592738	0.530430470	8.648160781
6	8.918445738	-0.379740530	8.791933781
6	10.021965738	-0.038484530	7.777647781
1	4.383046000	7.658844000	3.984383000

1	3.935509000	6.016243000	4.368561000
1	4.895602000	6.135656000	2.087053000
1	6.292836000	7.020964000	2.720203000
1	8.078360457	4.038482242	2.477627520
1	7.150576457	4.944754242	1.251828520
1	5.423199000	9.415176000	4.337933000
1	7.682502000	8.266485000	3.865190000
1	8.438743000	9.503809000	4.849755000
1	7.584406000	9.973155000	3.363600000
1	7.095371000	10.977712000	6.374497000
1	5.332754000	11.074549000	6.222983000
1	6.365871000	11.642648000	4.909539000
1	4.748934000	9.400952000	7.817195000
1	2.914754000	8.152279000	5.696422000
1	3.562790000	9.798248000	5.596938000
1	2.465963000	9.352477000	6.903839000
1	4.746576000	7.141358000	8.846175000
1	3.787945000	6.506578000	7.513062000
1	3.082021000	7.693096000	8.626851000
1	10.393717719	8.527553073	6.409648446
1	10.477746719	6.645877073	7.894158446
1	10.499474719	8.642824073	10.127086446
1	10.639307719	6.904995073	10.255865446
1	8.338596719	6.544752073	10.487658446
1	8.503367719	8.076079073	11.321793446
1	6.562271719	7.774276073	9.547745446
1	6.719391719	9.877768073	8.431040446
1	8.457883719	11.400820073	8.784903446
1	9.230481719	10.291411073	9.889952446
1	10.893095719	10.499699073	8.184353446
1	9.685921719	10.701203073	6.925872446
1	11.492887000	7.286623000	5.908235000
1	10.403101000	7.020262000	3.807334000
1	12.855832000	5.607895000	2.940713000
1	11.466298000	6.005649000	1.961101000
1	13.929639000	6.390691000	5.332821000
1	4.289774000	2.414345000	4.076488000
1	3.929130000	4.074725000	4.478793000
1	4.783198000	3.925406000	2.157113000
1	6.155144000	2.961103000	2.721611000
1	8.143529457	5.801915242	2.463559520
1	5.392537000	0.626295000	4.335667000
1	7.635860000	1.784992000	3.840853000
1	8.416920000	0.540927000	4.799899000

1	7.545038000	0.084482000	3.319769000
1	7.119138000	-0.984154000	6.288403000
1	5.351434000	-1.068418000	6.206977000
1	6.326912000	-1.613136000	4.840511000
1	4.783161000	0.609501000	7.851928000
1	2.892297000	1.810814000	5.753128000
1	3.567838000	0.176612000	5.648072000
1	2.489610000	0.609400000	6.974640000
1	4.773039000	2.891200000	8.843909000
1	3.771800000	3.484635000	7.523291000
1	3.114716000	2.304823000	8.672990000
1	11.745352000	2.912311000	6.167918000
1	10.320378000	2.654348000	4.238357000
1	12.118656000	3.432113000	2.357530000
1	10.442672000	3.934595000	2.246996000
1	13.649607000	4.240131000	4.327037000
1	10.447470738	1.645436470	6.436989781
1	10.390952738	3.460430470	7.995799781
1	10.477469738	1.375124470	10.149058781
1	10.531050738	3.111354470	10.347667781
1	8.209729738	3.352375470	10.542257781
1	8.435420738	1.800332470	11.322453781
1	6.514920738	2.070255470	9.523179781
1	6.799775738	0.023915470	8.328780781
1	8.608005738	-1.421378530	8.650975781
1	9.303766738	-0.322897530	9.816025781
1	11.004280738	-0.383561530	8.137485781
1	9.832086738	-0.585008530	6.846713781
1	13.160361000	5.879825000	6.825445000
1	13.898004000	3.816072000	6.003086000

130

Complex 5b Enthalpy (a.u) = 2847.420357

92	13.591874935	4.989675318	5.099521649
77	12.680630000	2.217052000	3.564813000
77	10.622541000	4.998042000	5.510872000
77	12.630636000	7.791798000	3.596381000
15	14.723598000	1.927210000	4.730127000
15	14.731287000	8.051152000	4.649323000
7	15.052609829	3.323095373	5.662391819
7	14.385104671	5.069485628	7.737382889
7	12.128133225	3.664617040	4.959005760
7	14.240390935	4.950676318	2.907541649
7	14.370462935	5.011826318	1.721713649

7	14.527652935	5.067255318	0.575097649
7	15.093723996	6.641537036	5.543461152
7	12.143630344	6.326635605	4.987950732
6	15.909183000	3.325399000	6.833654000
6	15.115707000	3.806019000	8.046351000
6	13.251514671	5.188220628	8.665107889
6	14.498173000	0.506490000	5.970957000
6	14.524489000	-0.873740000	5.311918000
6	13.209808000	0.689671000	6.773857000
6	16.324745000	1.469839000	3.820145000
6	17.477873000	1.040832000	4.732874000
6	16.768875000	2.620410000	2.916530000
6	10.762047000	1.247314000	3.525852000
6	10.702930000	2.322206000	2.609475000
6	10.756930000	2.145192000	1.102099000
6	12.199639000	2.229510000	0.570258000
6	13.227246000	1.728533000	1.564052000
6	13.141112000	0.505021000	2.284223000
6	11.988398000	-0.478625000	2.125246000
6	10.873422000	-0.213945000	3.148053000
6	8.986497000	3.607766000	5.505373000
6	9.570837000	3.638325000	6.799371000
6	8.913894000	4.294107000	8.007959000
6	7.677407000	4.269669000	5.130402000
6	16.034319000	6.591009000	6.645786000
6	15.296581000	6.237974000	7.938871000
6	14.581736000	9.452705000	5.923958000
6	14.611646000	10.847714000	5.296695000
6	13.318851000	9.279072000	6.769056000
6	16.282295000	8.513564000	3.658291000
6	17.488154000	8.927742000	4.508592000
6	16.675545000	7.376373000	2.714854000
6	8.957951000	6.377307000	5.694272000
6	9.755781000	6.401065000	6.868129000
6	9.363408000	5.757479000	8.182274000
6	7.586512000	5.716291000	5.637203000
6	10.677567000	8.703645000	3.626315000
6	10.619406000	7.643570000	2.697577000
6	10.627436000	7.840028000	1.192613000
6	12.056011000	7.795960000	0.619331000
6	13.099350000	8.316402000	1.585810000
6	13.004107000	9.528765000	2.324648000
6	11.820459000	10.482786000	2.218736000
6	10.744748000	10.173283000	3.271379000

1	16.800797000	3.948236000	6.680746000
1	16.294339000	2.326217000	7.076186000
1	14.361423000	3.046666000	8.269535000
1	15.767933000	3.894571000	8.933528000
1	12.703415671	6.108979628	8.456085889
1	13.576574671	5.198013628	9.719172889
1	15.354121000	0.577665000	6.655075000
1	13.682583000	-0.988408000	4.623626000
1	14.431778000	-1.650518000	6.080741000
1	15.447536000	-1.070627000	4.759037000
1	13.095756000	1.708945000	7.149889000
1	13.193285000	-0.003569000	7.624063000
1	12.338932000	0.488364000	6.143660000
1	16.042857000	0.617419000	3.187860000
1	17.831789000	1.878235000	5.342076000
1	18.325715000	0.709418000	4.121246000
1	17.220260000	0.216280000	5.403332000
1	16.007154000	2.893892000	2.185210000
1	17.676984000	2.337589000	2.371010000
1	16.990539000	3.514811000	3.506618000
1	10.334712000	1.405136000	4.513776000
1	10.259731000	3.249521000	2.968396000
1	10.152445000	2.925878000	0.628051000
1	10.292005000	1.194162000	0.817914000
1	12.299287000	1.687250000	-0.383276000
1	12.444446000	3.273560000	0.349792000
1	14.230041000	2.107205000	1.362824000
1	14.086510000	0.036703000	2.548867000
1	11.596960000	-0.430982000	1.102997000
1	12.362400000	-1.501173000	2.250379000
1	11.085842000	-0.776059000	4.064869000
1	9.902962000	-0.582730000	2.780568000
1	9.218760000	2.751074000	4.882372000
1	10.259818000	2.826845000	7.035069000
1	7.823791000	4.235646000	7.913354000
1	9.163152000	3.723878000	8.910323000
1	6.821589000	3.673336000	5.484956000
1	7.613667000	4.269831000	4.035439000
1	16.838692000	5.872712000	6.442452000
1	16.538721000	7.551779000	6.814261000
1	14.672606000	7.095639000	8.202894000
1	16.008400000	6.084109000	8.768479000
1	12.565072671	4.352507628	8.512708889
1	15.460465000	9.344542000	6.574253000

1	13.745436000	10.995412000	4.645645000
1	14.564573000	11.607611000	6.086279000
1	15.515512000	11.040516000	4.711977000
1	13.187318000	8.250080000	7.111485000
1	13.354871000	9.941070000	7.643312000
1	12.431561000	9.529181000	6.180271000
1	15.965609000	9.375808000	3.056112000
1	17.895807000	8.072735000	5.056570000
1	18.286979000	9.297307000	3.854418000
1	17.266088000	9.720035000	5.228740000
1	15.892553000	7.138031000	1.993815000
1	17.574128000	7.654879000	2.151579000
1	16.894852000	6.463016000	3.275866000
1	9.079549000	7.195373000	4.993123000
1	10.456175000	7.231621000	6.968308000
1	8.581388000	6.346686000	8.686571000
1	10.237712000	5.788678000	8.842373000
1	7.111649000	5.751678000	6.624565000
1	6.934287000	6.297203000	4.975399000
1	10.293066000	8.513749000	4.626074000
1	10.222589000	6.696423000	3.060283000
1	10.027364000	7.051544000	0.725854000
1	10.133932000	8.783943000	0.934455000
1	12.112924000	8.346107000	-0.333172000
1	12.321595000	6.759794000	0.385584000
1	14.104450000	7.969564000	1.344436000
1	13.945497000	10.019377000	2.563033000
1	11.397982000	10.441114000	1.208580000
1	12.170352000	11.513132000	2.349438000
1	10.973174000	10.724576000	4.190895000
1	9.755244000	10.526107000	2.940833000
